# Identification of molecular subtypes, risk signature, and immune landscape mediated by necroptosis-related genes in non-small cell lung cancer

**DOI:** 10.3389/fonc.2022.955186

**Published:** 2022-07-28

**Authors:** Jiaqi Zhu, Jinjie Wang, Tianyi Wang, Hao Zhou, Mingming Xu, Jiliang Zha, Chen Feng, Zihao Shen, Yun Jiang, Jianle Chen

**Affiliations:** ^1^ Nantong Key Laboratory of Translational Medicine in Cardiothoracic Diseases, and Research Institution of Translational Medicine in Cardiothoracic Diseases, Affiliated Hospital of Nantong University, Medical School of Nantong University, Nantong, China; ^2^ Department of Thoracic Surgery, Affiliated Hospital of Nantong University, Medical School of Nantong University, Nantong, China; ^3^ Department of Burn and Plastic Surgery, Affiliated Hospital of Nantong University, Medical School of Nantong University, Nantong, China

**Keywords:** necroptosis, non-small cell lung cancer, tumor microenvironment, immune, prognostic biomarker

## Abstract

**Background:**

Non-small cell lung cancer (NSCLC) is a highly heterogeneous malignancy with an extremely high mortality rate. Necroptosis is a programmed cell death mode mediated by three major mediators, RIPK1, RIPK3, and MLKL, and has been shown to play a role in various cancers. To date, the effect of necroptosis on NSCLC remains unclear.

**Methods:**

In The Cancer Genome Atlas (TCGA) and Gene Expression Omnibus (GEO) databases, we downloaded transcriptomes of lung adenocarcinoma (LUAD) patients and their corresponding clinicopathological parameters. We performed multi-omics analysis using consensus clustering based on the expression levels of 40 necroptosis-related genes. We constructed prognostic risk models and used the receiver operating characteristic (ROC) curves, nomograms, and survival analysis to evaluate prognostic models.

**Results:**

With the use of consensus clustering analysis, two distinct subtypes of necroptosis were identified based on different mRNA expression levels, and cluster B was found to have a better survival advantage. Correlation results showed that necroptosis was significantly linked with clinical features, overall survival (OS) rate, and immune infiltration. Kyoto Encyclopedia of Genes and Genomes (KEGG) and Gene Ontology (GO) enrichment analysis confirmed that these differential genes were valuable in various cellular and biological functions and were significantly enriched in various pathways such as the P53 signaling pathway and cell cycle. We further identified three genomic subtypes and found that gene cluster B patients had better prognostic value. Multivariate Cox analysis identified the 14 best prognostic genes for constructing prognostic risk models. The high-risk group was found to have a poor prognosis. The construction of nomograms and ROC curves showed stable validity in prognostic prediction. There were also significant differences in tumor immune microenvironment, tumor mutational burden (TMB), and drug sensitivity between the two risk groups. The results demonstrate that the 14 genes constructed in this prognostic risk model were used as tumor prognostic biomarkers to guide immunotherapy and chemotherapy. Finally, we used qRT-PCR to validate the genes involved in the signature.

**Conclusion:**

This study promotes our new understanding of necroptosis in the tumor microenvironment of NSCLC, mines prognostic biomarkers, and provides a potential value for guiding immunotherapy and chemotherapy.

## Introduction

Lung cancer is one of the most common cancers worldwide and the leading reason of cancer-related deaths worldwide (1). Every year, there are nearly 1.8 million new cases and 1.76 million deaths worldwide (2, 3). Lung cancer is mainly divided into two categories according to pathology: non-small cell lung cancer (NSCLC) and small cell lung cancer (SCLC) (4). Among them, NSCLC accounts for the largest proportion of all patients, about 85%, and has a very high mortality (5). With the continuous improvement of medical level, surgical treatment, immunotherapy, chemotherapy, and other comprehensive tumor treatment methods have changed the prognosis of many patients. However, patients were often diagnosed with advanced cancer, so mortality rates were not reduced (6, 7). The 5-year survival rate for patients is nearly 5%–18%, depending on the patient’s tumor stage (8). Therefore, we urgently need new treatment options to improve the clinical prognosis of NSCLC and improve the prognostic value of patients.

Necroptosis is a regulated form of programmed cell death (9, 10), which is morphologically similar to necroptosis (11), manifested by the co-swelling of organelles and cytoplasm, allowing rapid permeation of the plasma membrane. It is metabolized and ruptured, and all the cell contents are lost to the intercellular space (12, 13). Necroptosis has been proved to play a vital role in the pathophysiology and mechanism of action of a variety of clinical diseases, such as acute kidney injury, cardiac ischemia–reperfusion, and various inflammatory diseases including Crohn’s disease and acute pancreatitis (14–18). We also found that necroptosis is dual-sided in tumors (19). On the one hand, it acts as a safety mechanism to prevent tumor development and promote cancer treatment when the apoptotic process is compromised. For example, ectopic activation of RIPK3, an important mediator of the necroptosis pathway, is associated with enhanced CD8+ leukocyte-mediated antitumor effects, resulting in systemic tumorigenesis and invasion inhibition and increased chemotherapeutic drug sensitivity (20–22). On the other hand, necroptosis accelerates cancer progression and metastatic progression (23, 24). Related studies have shown that necroptosis promotes macrophage-induced adaptive immunosuppression through the co-expression of regulated CXCL1 and Mincle factors, thereby enabling pancreatic tumor progression; DR6-mediated tumor cells induce endothelial cell necroptosis, which in turn leads to tumor cell extravasation and metastasis (25, 26). So far, the specific mechanism by which necroptosis is involved in NSCLC tumor microenvironment (TME) remains unclear.

In the present study, we used consensus clustering analysis based on the mRNA and protein expression levels of necroptosis-related genes in The Cancer Genome Atlas (TCGA) and Gene Expression Omnibus (GEO) databases and identified two distinct types of necroptosis. Apoptotic subtypes were assessed for their immune responses, pathways, and expression of clinicopathological factors. Next, after the genomic subtypes were established, a model was established based on the best prognostic genes to comprehensively evaluate the value of necroptosis-related genes in clinicopathological features, TME, immune-related responses, drug sensitivity, and prognostic treatment of NSCLC.

## Materials and methods

### Data source

In the text, TCGA database was used to obtain transcriptome data of NSCLC patients and their corresponding clinicopathological data and overall survival (OS) information, including gene expression FPKM (fragments per kilobase of transcript per million mapped reads) values of 1,037 NSCLC patients and 108 normal samples. In the follow-up study, we further converted the FPKM value into the TPM (transcripts per kilobase million) value; in addition, we eliminated the data with incomplete survival information to ensure the accuracy of the data. Four cohorts (GSE50081, GSE68465, GSE31210, and GSE37745) were obtained from the GEO database to further validate prognostic gene signatures and risk assessment capabilities. Especially, we used the combat function in the ‘sva’ package to remove batch effects in different datasets. In addition, we downloaded the copy number variation (CNV) frequency of somatic mutations from TCGA.

### Molecular subtyping based on necroptosis-related genes

Based on previous in-depth research on necroptosis, we obtained 40 necroptosis-related genes (**Supplementary Table S1**) for further analysis. We used unsupervised clustering to identify patterns of modification distinct from necroptosis factor expression based on mRNA expression levels of 40 necroptotic genes in two cohorts, TCGA-NSCLC and GSE50081. Unsupervised clustering is a relatively common way of classifying cancer subtypes. In this process, we used the ‘ConsensusClusterPlus’ R package to define patients into different molecular subtypes based on different expression levels. The consensus matrix heatmap identified the optimal number of clusters for the distribution of NSCLC subtypes.

### Identification and functional analysis of differentially expressed genes between different subtypes

In order to identify necroptosis-related genes, we divided the total sample size into two types based on the expression levels of 40 different necroptosis factors, which were defined as cluster A/B, and we used the ‘limma’ R package to classify NSCLC patients based on the empirical Bayesian approach. Crossover genes between two different types were identified, and significance criteria were defined as follows: adjusted p-value < 0.05 and absolute value of Log2 FC ≥ 1.5. We performed enrichment analysis using the ‘ClusterProfiler’ R package to further explore their potential pathways and functional enrichment pathways. In addition, we re-validated its functional enrichment analysis using Metascape online analysis software and defined a p-value < 0.05 as a critical threshold. Next, based on the ‘glmnet’, ‘survminer’, and ‘survival’ R packages, we screened out 89 prognostic-related genes using univariate Cox analysis to construct genomic subtypes, and then we used multivariate Cox analysis to identify 14 differentially expressed genes (DEGs) again; the prognostic risk assessment construction formula is as follows:


Risk score = coefficient1 * expression of gene1 + ··· + coefficientN * expression of geneN

### Gene set variation analysis

We performed gene set variation analysis (GSVA) enrichment analysis using the ‘GSVA’ R package. We obtained the ‘c2.cp.kegg.v7.4.symbols.gmt’ file on the MSigDB database using the ‘limma’ R package after adjustments. Under the screening conditions of p-value < 0.05 and false discovery rate (FDR) < 0.05, the corresponding heatmap was drawn, which created a prerequisite for us to study its potential biological functions and pathways (27).

### Tumor microenvironment and immune correlation analysis

We used single-sample gene set enrichment analysis (ssGSEA) to correlate different molecular subtypes with the tumor microenvironment to calculate the level of infiltration of 23 immune cell types and assess their immune function and correlations. At the same time, we used the ‘ESTIMATE’ R package to evaluate the immune and stromal scores of each NSCLC patient sample. In addition, for further validation, the CIBERSORT algorithm was used to calculate the scores of 22 selected immune cells in NSCLC patient samples. This algorithm is based on the analysis of LM22 immune gene parameters. It is run against a 1,000 permutation distribution. When the p-value of the deconvolution results is < 0.05, the samples with low accuracy are removed. Spearman’s correlation was used to evaluate the correlation between risk score and immune cell infiltration level.

### Construction of a necroptosis-related prognostic model

We used univariate Cox analysis to screen out DEGs in two molecular subtypes, a total of 89, to study the survival prognosis, enrichment analysis, and potential biological functions of NSCLC patients. We then used unsupervised clustering analysis to classify it into the three best necroptosis genomic subtypes, defined as gene clusters A/B/C, which we used for further analysis. We continued to identify the 14 best differentially expressed genes to construct a prognostic model. This prognostic model uses the corresponding median value as a cutoff point to classify NSCLC patients in the entire dataset into high and low groups. The ‘survival’ and ‘survminer’ R packages were used to assess their differences in survival prognosis. In addition, the receiver operating characteristic (ROC) curve analysis was performed to plot the area under the curve (AUC) based on different years and different clinicopathological data. We used the ‘scatterplot3d’ R package to perform principal component analysis (3DPCA) to further verify the accuracy of the grouping. We used the ‘regplot’ R package to draw nomograms from clinicopathological data. We excluded the M stage due to the lack of data available for analysis. We can use nomograms to look at 1-, 3-, and 5-year OS probabilities for NSCLC to further calculate the accuracy of factual and predicted survival. We also created heatmaps assessing the distribution of different clinicopathological data in different groups.

### Drug sensitivity analysis

To explore the prognostic differences of chemotherapeutic drugs in high- and low-risk groups of NSCLC patients, the chemosensitivity was evaluated. We calculated the half-inhibitory concentration values (IC50) of chemotherapeutic drugs routinely used in the treatment of NSCLC patients based on the ‘ggpubr’, ‘pRRophetic’ R packages. Data on chemotherapy drugs were obtained from the Genomics of Drug Sensitivity in Cancer (GDSC) database. In pRRophetic algorithm, more than 700 cell lines included in the Cancer Genome Project (CGP) database were selected to develop a drug response prediction using the expression matrix, and the reliability of the algorithm was validated in other datasets.

### Collection and validation of clinical samples

NSCLC tissues were obtained from patients who had undergone surgery at the Affiliated Hospital of Nantong University. In our cohort, 10 pairs of tissues were obtained between 2015 and 2020. The study was authorized by the Ethical Committee of Affiliated Hospital of Nantong University (2022-L048). In a previous study, we used a high-capacity reverse transcription kit (Takara, Maebashi, Japan) for the reverse transcription of total RNA to cDNA, and cDNA was frozen in the long term. In this research, we used the previous cDNA to conduct a qRT-PCR assay in Light Cycler 480II (Roche, Basel, Switzerland) using SYBR Green technology (Takara), and primer sequences were obtained from a primer bank. Primer sequences were from previous references (28–39) and primer bank (https://pga.mgh.harvard.edu/primerbank/). In addition, 2^−Δct^ was used to normalize and simplify to generate a riskscore. Riskscore _(qRT-PCR)_ = (Score − Min)/Max.

### Statistical analysis

Spearman’s and distance correlation analyses calculated correlation coefficients. Differences in survival prognosis among subgroups were calculated by the Kaplan–Meier analysis and log-rank test, and correlation maps were drawn. The Wilcoxon test was used for continuous variable analysis between two groups; the Kruskal–Wallis test was also used for more groups. In this paper, all data analyses were performed using R software (version 4.0.4).

## Results

### Genetic changes and expression characteristics of necroptosis-related genes in non-small cell lung cancer

**Figure 1:**
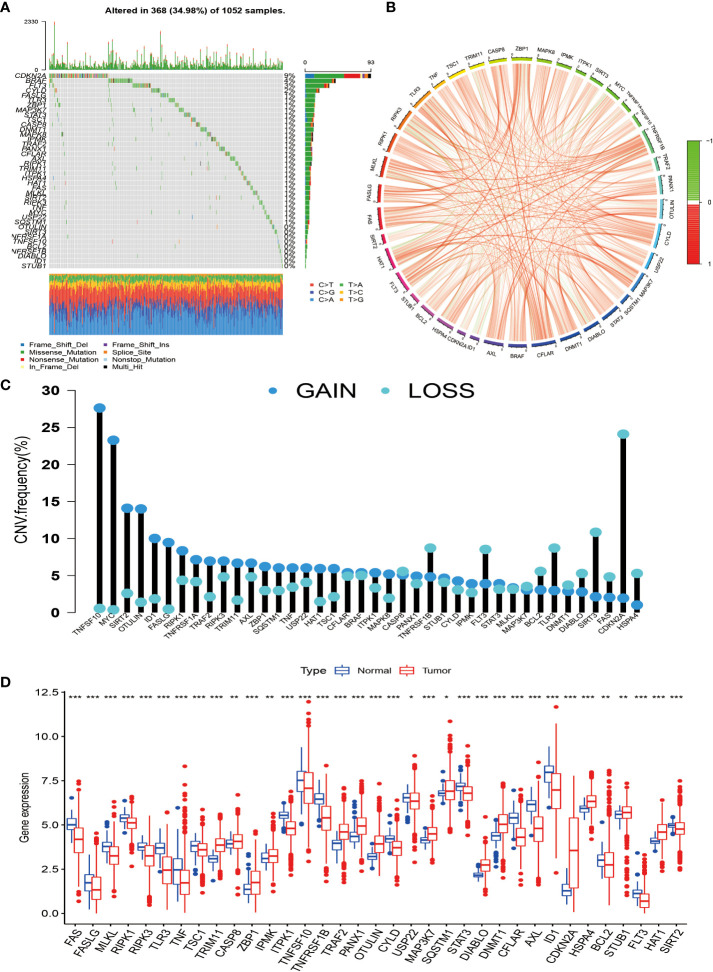
Genetic changes and expression characteristics of necroptosis-related genes in non-small cell lung cancer. **(A)** Mutation frequencies of necroptosis-related genes in NSCLC; each column represents an individual patient, numbers, and bars on the right of the graphs represent mutation frequencies for each regulator, with stacked bars below representing transformations for each sample. **(B)** Circle plot drawn by correlation analysis showing the association of all necroptosis genes. **(C)** The graph depicts the frequency of CNV alterations in 40 necroptosis-related genes in NSCLC. **(D)** Regulation between mRNA expression levels of necroptosis-related genes. Asterisks represent p-values (*p < 0.05; **p < 0.01; ***p < 0.001). NSCLC, non-small cell lung cancer; CNV, copy number variation.

In this study, we selected 40 necroptosis-related genes. In TCGA-NSCLC cohort, the incidence of somatic mutations and CNV of NG in NSCLC patients was analyzed. Among the 1,052 samples, a total of 368 samples had necroptosis-related gene mutations, with a frequency of 34.98%. Among them, CDKN2A had the highest mutation frequency at 9%, followed by BRAF, FLT3, and CYLD. However, OTULIN, SIRT3, NFRSF1A, TNFSF10, BCL2, NFRSF1B, DIABLO, ID1, and STUB1 showed almost no mutation in NSCLC (**Figure 1A**). We also drew a circle plot to represent the association of the 40 necroptosis-related genes with each other (**Figure 1B**). From the frequency of copy number variation, it can be seen that 40 NGs have extensive CNV alterations and are mainly concentrated on the amplification of CNV alterations, of which 28 show copy number amplification and the remaining 12 are lost (**Figure 1C**). We further explored whether changes in copy number variation frequency had a significant effect. It was found that some necroptosis-related genes with amplified CNV alterations had higher expression in NSCLC tissues (such as SIRT2 and OTULIN), so we predicted that CNV alterations were a factor affecting the expression of NG (**Figures 1C, D**). In conclusion, the genetic variation and mRNA expression levels of necroptosis-related genes in NSCLC samples and normal tissues were significantly different, indicating that they play a potential role in the occurrence and development of NSCLC.

### Identification of necroptosis subtypes in non-small cell lung cancer

**Figure 2:**
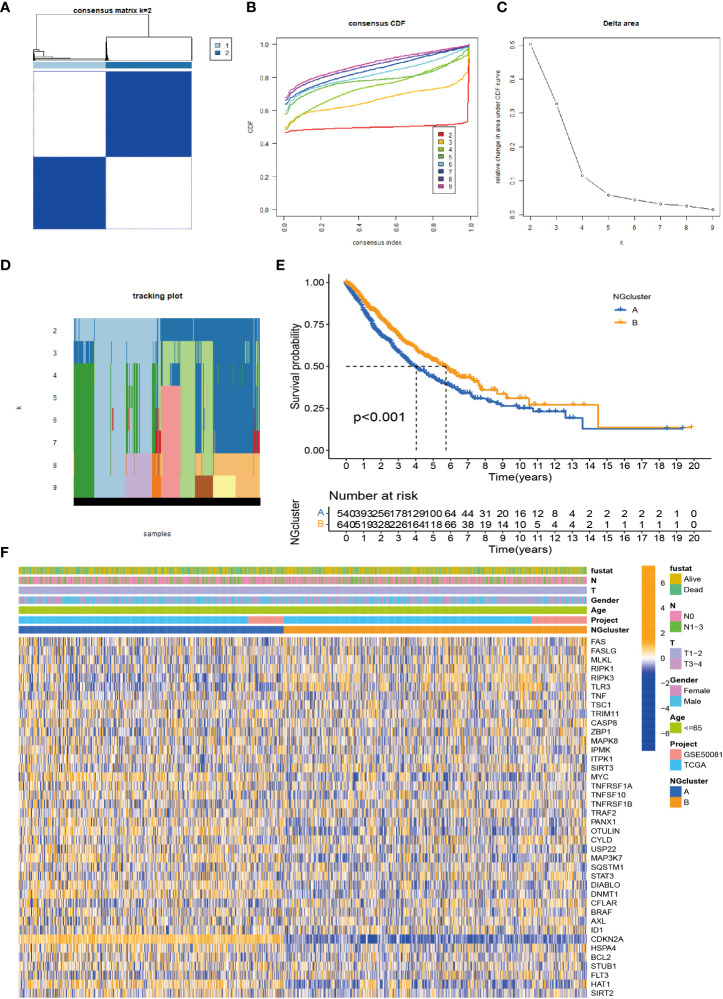
Identification of necroptosis subtypes in NSCLC. **(A)** Consensus clustering of NSCLC patients with k = 2. **(B)** Consensus cluster CDF for k = 2–9. **(C)** CDF curve of consensus clustering. **(D)** Trace plots for k = 2 to 9. **(E)** p-Value < 0.001 for Kaplan–Meier curve, a significant difference in survival between the two subtypes. The survival advantage of group B was better. **(F)** Heatmap showing the relationship between the two clusters on clinicopathological parameters. NSCLC, non-small cell lung cancer; CDF, cumulative distribution function.

To investigate the modification patterns of necroptosis-related genes in NSCLC, we pooled TCGA-NSCLC and GSE50081 patients into a cohort for further analysis for our subtype establishment. First, we used KM analysis to prove that 28 necroptosis genes have prognostic significance for survival (**Supplementary Figure S1**). The screening principle of this method is that when the p-value is less than 0.05, it has an obvious prognostic value. Next, we used a consensus clustering algorithm to study the relationship between these necroptosis genes and NSCLC subtypes. When k = 2, the features with the highest intra-group correlation and the lowest inter-group correlation are the best choices. Therefore, we divided NSCLC patients into two groups with the most reliable and stable results, defined as cluster A/B (**Figures 2A–D**; **Supplementary Figures S2A–**). The Kaplan–Meier curve shows a larger OS value for cluster B, with a better survival advantage (**Figure 2E**). In addition, we also created a heatmap to compare the differences in clinicopathological data such as age, gender, TNM stage, and survival status between the two subtypes (**Figure 2F**).

### Enrichment analysis and immune landscape of distinct necroptosis subtypes in non-small cell lung cancer

**Figure 3:**
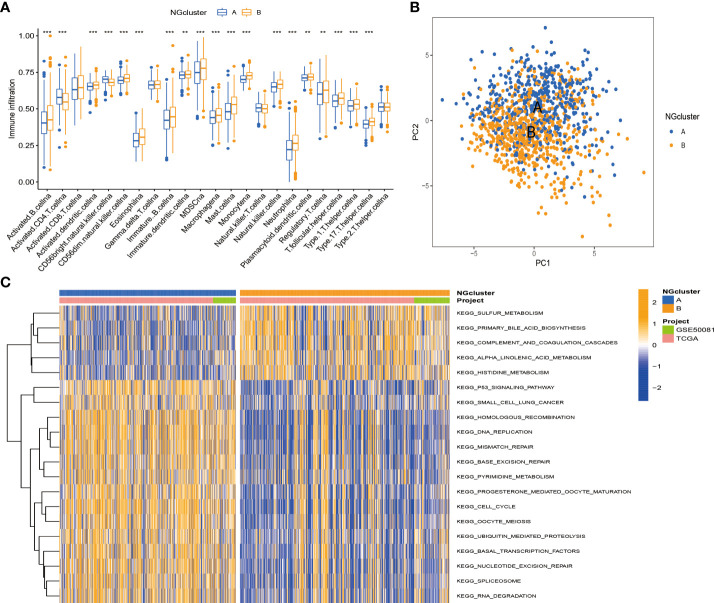
Enrichment analysis and immune landscape of distinct necroptosis subtypes in NSCLC. **(A)** Infiltrating abundance of two clusters in 23 immune cell types. **(B)** PCA classified NSCLC into two distinct clusters. **(C)** GSVA enrichment analysis revealed activation of various pathways under different patterns of necroptosis modification. NSCLC, non-small cell lung cancer; PCA, principal component analysis; GSVA, gene set variation analysis. **P < 0.01; ***P < 0.001

According to previous studies, we found that necroptosis has multiple biological functions and clinical value. We validated the identification of two necroptosis subtypes using principal component analysis (PCA) (**Figure 3B**). To further analyze the underlying biological features of the two different subtypes, we performed a GSVA enrichment analysis. Cluster A is significantly enriched in cell cycle, basal and oncogenic pathways, and nucleotide metabolism pathways, including p53 signaling, small cell lung cancer, DNA replication, and pyrimidine metabolism. Progesterone mediates oocyte maturation, cell cycle, oocyte meiosis, basal transcription factors, nucleotide excision repair, spliceosomes, and RNA interpretation. Cluster B has significant enrichment characteristics in amino acid metabolism pathways, including sulfur metabolism, complement system, and histidine metabolism (**Figure 3C**). In addition, to investigate the immune correlation, we also used ssGSEA to evaluate the differential correlation between two different necroptosis subtypes and 23 human immune cells. It was evident that cluster B has a higher infiltrating abundance compared to cluster A (**Figure 3A**).

**Figure 4:**
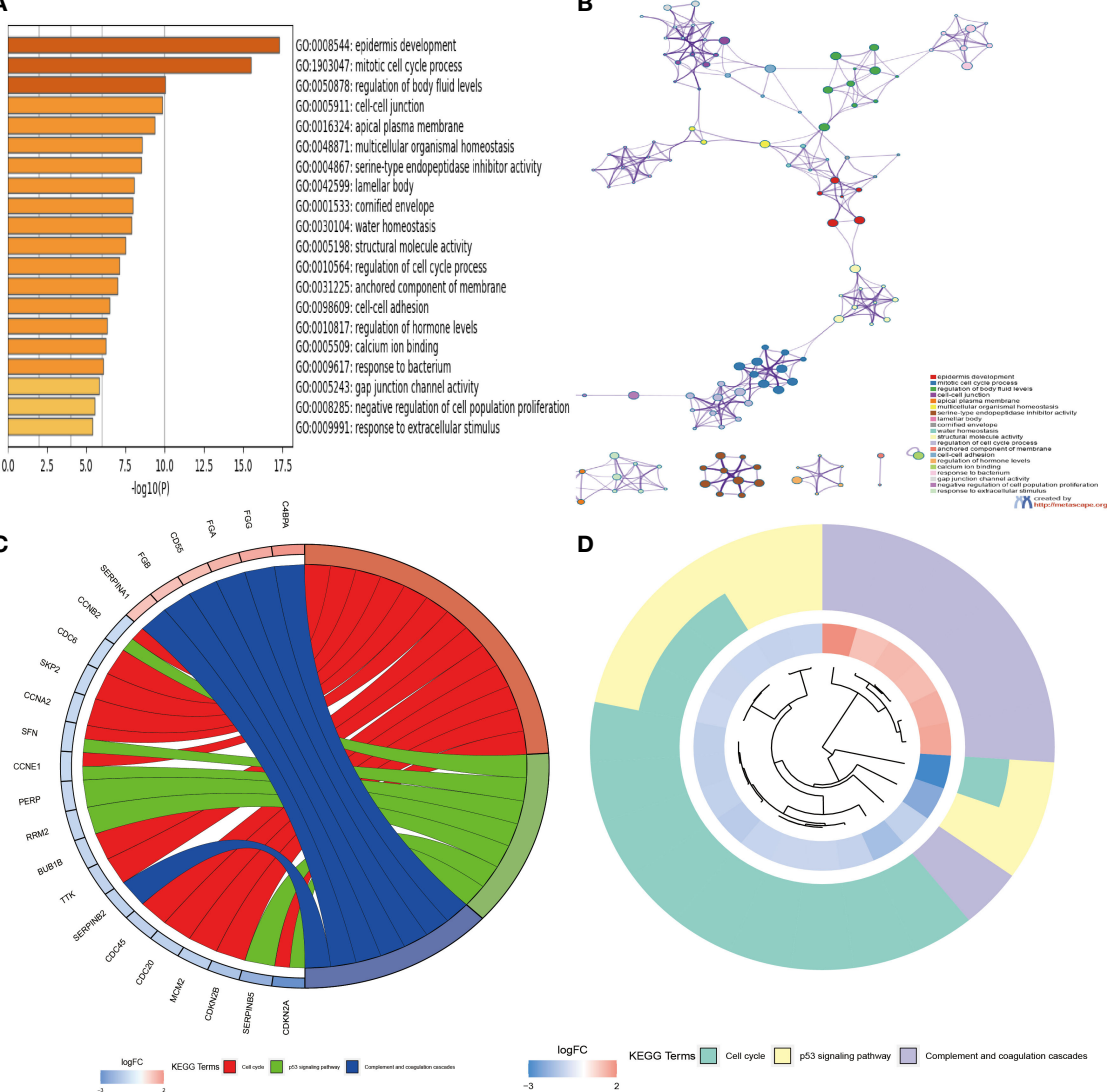
**(A, B)** Functional annotation of GO enrichment analysis by Metascape software. **(C, D)** Functional annotation of KEGG enrichment analysis. GO, Gene Ontology; KEGG, Kyoto Encyclopedia of Genes and Genomes.

To investigate the function and mechanism of action of genes enriched in necroptosis subtypes, we screened 293 intersection genes of two different subtypes for enrichment analysis using the ‘limma’ R package. With Gene Ontology (GO) enrichment analysis by Metascape online analysis software, a variety of cellular and biological functional pathways were found to be involved in mitotic cell cycle process, cell–cell junction, regulation of humoral levels, epidemic infection, cell cycle, multicellular organism significant enrichment in homeostasis, and structural molecular activity (**Figures 4A,B**). Kyoto Encyclopedia of Genes and Genomes (KEGG) enrichment analysis showed significant enrichment trends in cell cycle, P53 signaling, complement, and coagulation cascades (**Figures 4C, D**).

### Construction of differentially expressed gene-based genotypes in non-small cell lung cancer

**Figure 5:**
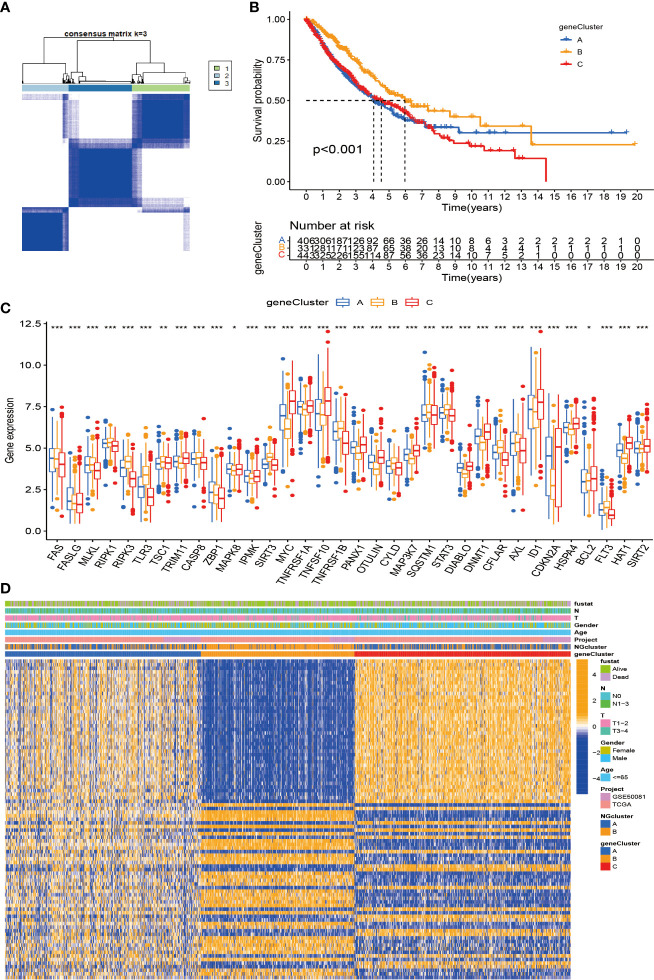
Construction of DEG-based genotypes in NSCLC. **(A)** Consensus clustering of NSCLC patients with k = 3. **(B)** Kaplan–Meier plots show that the significant OS rate of gene cluster B is significantly better than that of gene cluster A and gene cluster C, p-value < 0.001. **(C)** Expression levels of 40 necroptosis-related genes in three gene clusters. **(D)** Patients were grouped into three genomic subtypes, termed gene clusters A–C, using consensus clustering analysis, with survival, age, sex, and tumor stage as reference indicators for heatmaps. DEG, differentially expressed gene; NSCLC, non-small cell lung cancer; OS, overall survival. *p < 0.05; **p < 0.01; ***P < 0.001.

First, we used univariate Cox analysis to identify 89 DEGs for subsequent prognostic analysis (**Supplementary Table S2**). In order to further verify this regulatory mechanism and explore potential gene functions and activation pathways, we used an unsupervised consensus clustering algorithm to divide them into three gene subtypes, called gene clusters A–C (**Figure 5A**; **Supplementary Figures S3A–C**). The Kaplan–Meier curve shows that gene cluster B has the best survival prognosis (**Figure 5B**). Next, we analyzed the three genomic subtypes based on the expression levels of 40 necroptosis-related genes, showing significant differences (**Figure 5C**). At the same time, we analyzed the expression of the three gene subtypes in clinicopathological parameters, including gender, age, survival status, and TNM stage, all of which showed good differences (**Figure 5D**).

### Construction and evaluation of prognostic risk model

**Figure 6:**
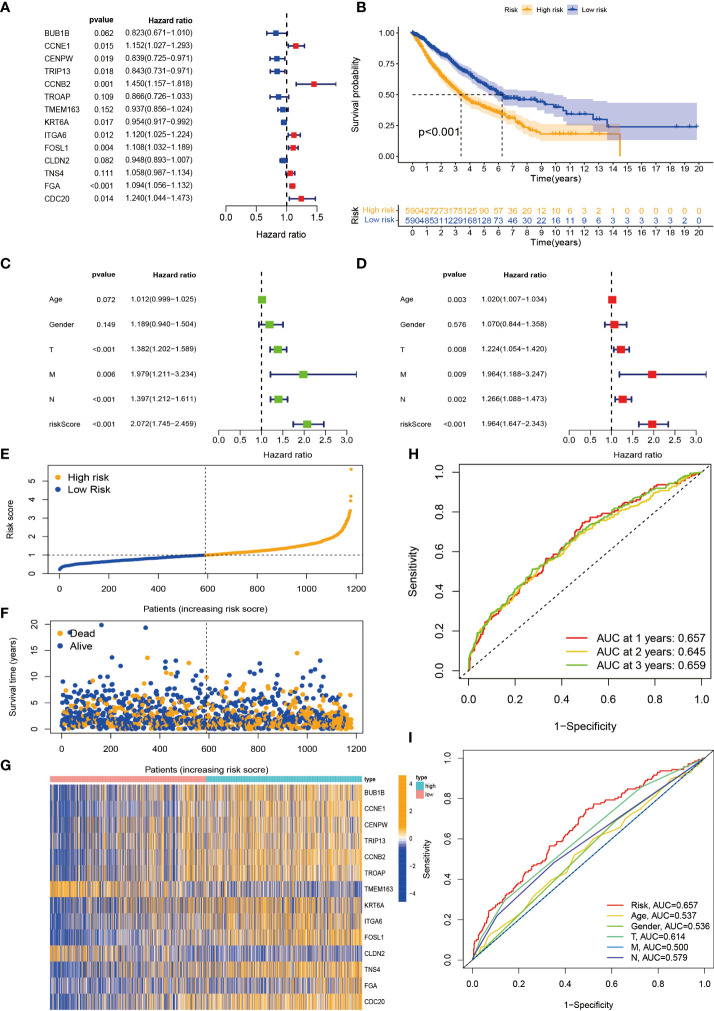
Construction and evaluation of prognostic risk model. **(A)** Forest plot of the best prognostic genes. **(B)** Kaplan–Meier curves for survival analysis of patients in high-risk and low-risk groups. **(C, D)** Univariate and multivariate Cox analyses of clinicopathological features and risk scores. **(E, F)** Risk score distribution and survival status. **(G)** Expression heatmap of the 14 best prognostic genes. **(H)** ROC curves predict 1-, 2-, and 3-year survival. **(I)** ROC curves predict the prognostic efficiency of different clinical features and risk scores. ROC, receiver operating characteristic.

**Figure 7:**
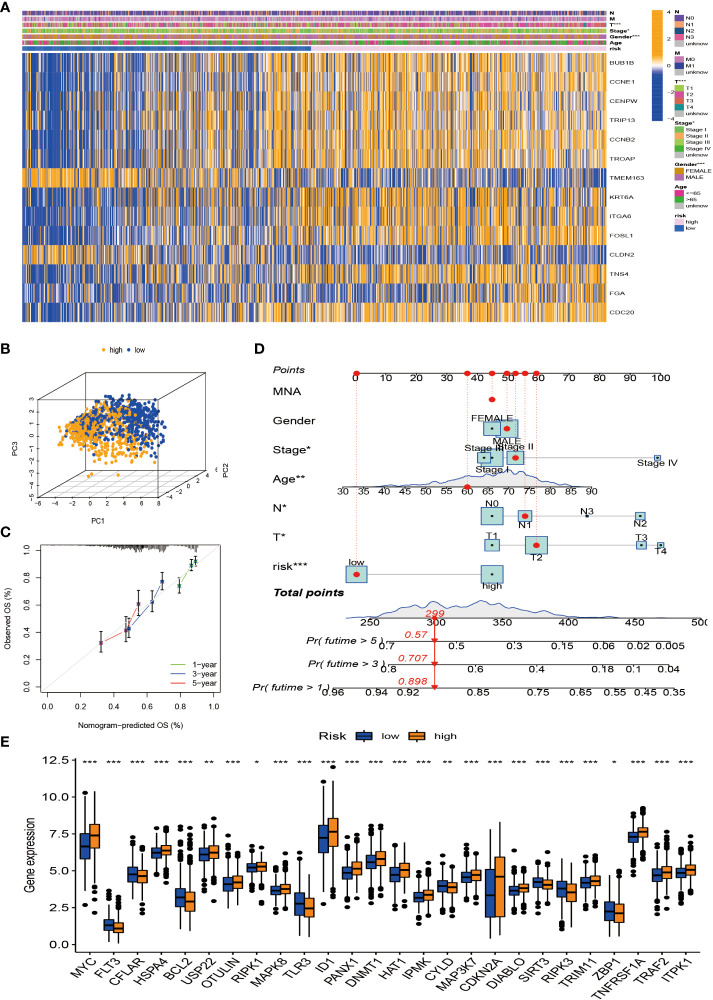
Tumor microenvironment (TME) and tumor mutational burden (TBM) analysis among patients in different risk groups. **(A)** Heatmap is used to show correlations between clinical characteristics and risk groups. **(B)** PCA of high-risk and low-risk pairs of prognostic risk models including 14 necroptosis-related genes. **(C)** Calibration curve predicted by nomogram. **(D)** Combined nomogram of risk score and other clinicopathological factors. **(E)** Expression levels of necroptosis-related genes in high-risk and low-risk groups. ROC, receiver operating characteristic; PCA, principal component analysis. *p < 0.05; **p < 0.01; ***P < 0.001.

Considering that the molecular subtypes are population-based, we performed a risk score for each individual through the above DEGs. Through multivariate Cox analysis, 14 DEG-based best prognosis-related genes were identified for constructing prognostic risk models, including seven risk genes with hazard ratios (HRs) > 1 (*CCNE1*, *CCNB2*, *ITGA6*, *FOSL1*, *TNS4*, *FGA*, and *CDC20*) and seven protected genes (*BUB1B*, *CENPW*, *TRIP13*, *TROAP*, *TMEM163*, *KRT6A*, and *CLDN2*) with HRs < 1 (**Figure 6A**; **Supplementary Table S3**). From the results of the multivariate Cox analysis, we calculated a risk score for each patient, and then we divided the patients into two risk groups based on the median. Survival prognostic analysis revealed significant differences, with patients in the high-risk group having a worse prognosis as compared to the low-risk group (**Figure 6B**). We performed univariate Cox analysis and multivariate Cox analysis to verify the stability of the prognostic risk model. Univariate Cox analysis showed that the risk score was p < 0.001, HR = 2.072, CI = 1.745–2.459, which was significantly associated with the survival difference of NSCLC patients; in multivariate Cox analysis, the risk score was p < 0.001, HR = 1.964, CI = 1.647–2.343, which can be used as an independent prognostic factor and has strong predictability, indicating that the prognostic risk model we constructed is an independent prognostic indicator (**Figures 6C, D**). From the patient survival status, risk score distribution, and gene expression heatmap, it can be seen that the high-risk group had higher mortality and higher gene expression distribution (**Figures 6E–G**). When ROC curve analysis was performed on the 1-, 2-, and 3-year survival rates, the AUC values were 0.657, 0.645, and 0.659, respectively. In addition, we also performed ROC analysis for different clinicopathological parameters, and the value was 0.657. It is sufficient to demonstrate that this prognostic risk model has good predictability for the survival rate of NSCLC patients (**Figures 6H, I**). The drawn heatmap showed significant differences in tumor stage, age, gender, and TNM stage between the high-risk and low-risk groups (**Figure 7A**). PCA again verified that prognostic risk genes were able to differentiate the two risk groups, high-risk and low-risk groups (**Figure 7B**). The nomogram transforms the complex regression equation into a simple and visualized graph, which makes the results of the prediction model more readable. In order to quantitatively evaluate the overall OS value of NSCLC patients based on the risk score and clinicopathological data, we drew a nomogram, and the AUC values that effectively predicted the 1-year, 3-year, and 5-year overall survival rates were 0.898, 0.707, and 0.57, respectively (**Figure 7C**). The calibration plots showed good performance and accuracy for the nomogram predictions (**Figure 7D**). The Sankey diagram showed distributional associations between two necroptosis subtypes, three genotypes, two risk groups, and survival status (**Figure S3D**). The Kruskal–Wallis test showed significant differences between necroptosis subtypes and genotypes (**Supplementary Figures S3E,F**). In addition, we also explored differences in necroptosis-related genes between the two risk groups, showing significant differences overall (**Figure 7E**).

### Tumor microenvironment and tumor mutational burden analysis among patients in different risk groups

**Figure 8:**
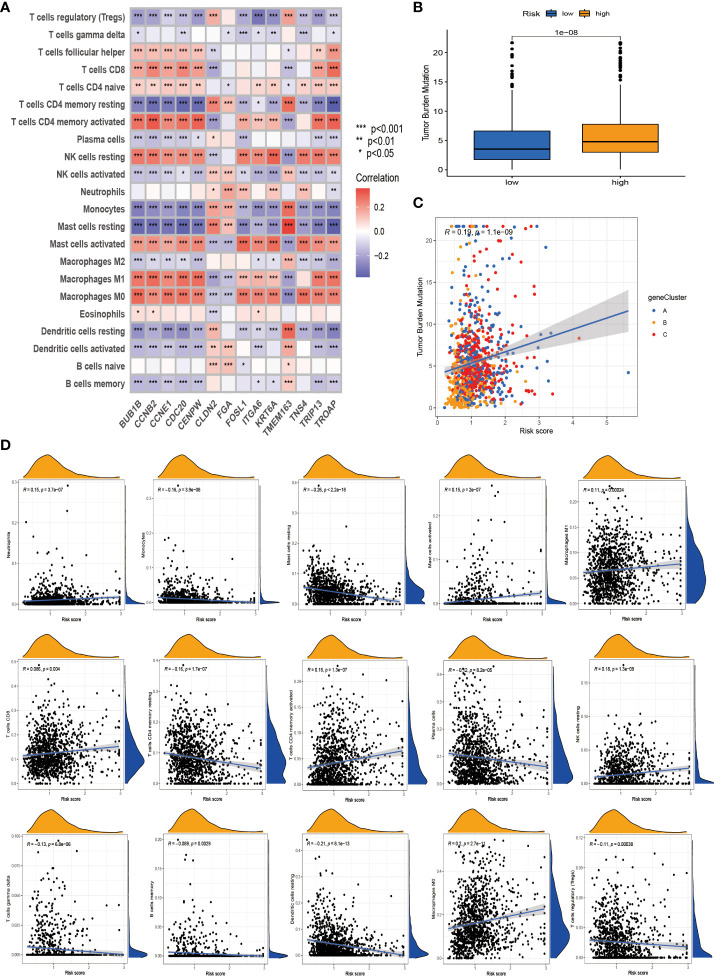
Tumor microenvironment (TME) and tumor mutational burden (TBM) analysis among patients in different risk groups. **(A)** Association of the 14 best genes used to construct prognostic risk models with immune cell infiltration. **(B)** Differential levels of TMB between high-risk and low-risk groups. **(C)** Correlation of TMB and risk score. **(D)** Correlation analysis. *p < 0.05; **p < 0.01; ***P < 0.001.

We first used the CIBERSORT algorithm to assess immune cell abundance for 14 prognostic risk genes and found generally high correlations (**Figure 8A**). It can be seen from the scatter plot that neutrophils, mast cells activated, macrophages M1, NK cells resting, T cells CD8, T cells CD4 memory activated, and macrophages M0 are positively correlated with the risk score and negatively correlated with the risk score, including monocytes, mast cells resting, T cells CD4 memory resting, plasma cells, T cells gamma delta, B cells memory, dendritic cells resting, and T cells regulatory (Tregs) (**Figure 8D**). It is not difficult to find that these 14 prognosis-related genes play a crucial role in the TME of NSCLC. In addition, we performed ESTIMATE procedures on two different risk groups and simultaneously assessed their stromal score, immune score, and ESTIMATE score. We found that the low-risk group had a higher TME score and more aggregation of immune cells and stromal cells (**Figure S3G**). According to previous clinical research studies, it was found that somatic tumor mutational burden (TMB) has a strong sensitivity to immunotherapy and is closely related to the intensity of treatment and survival rate. Next, we assessed the relationship between TMB and risk score and found that the high-risk group had higher TMB values, suggesting that it may have a better immunotherapy effect (**Figure 8B**). The TMB values of different genotypes were significantly positively correlated with the risk score (**Figure 8C**). These results suggest that TMB has a certain impact on the immune prediction, immunotherapy, and clinical prognosis of patients.

### Drug sensitivity analysis

**Figure 9:**
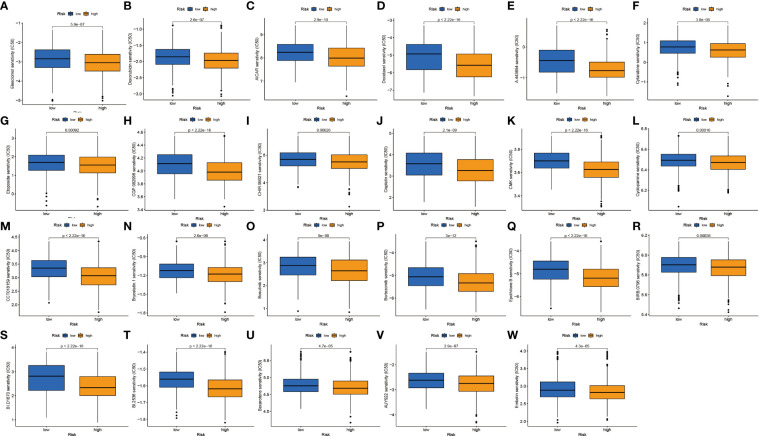
Drug sensitivity analysis. **(A–W)** IC50 values of 23 chemotherapeutic drugs between high-risk and low-risk groups.

We continued to investigate the association between risk scores and chemotherapeutic sensitivity. We obtained chemotherapeutic drugs currently used to treat patients with NSCLC in the GDSC database to assess their susceptibility to them in high- and low-risk groups. It can be found that patients in the low-risk group have a higher sensitivity to chemotherapy drugs such as cisplatin, irisimo, doxorubicin, acadesine, docetaxel, cytarabine, cyclopamine, and bosutinib (**Figures 9A–W**). These results also suggest that patients in the high-risk group lack sensitivity to chemotherapeutic drugs and have a worse prognosis.

### Three gene expression omnibus databases validate prognostic risk signatures

**Figure 10:**
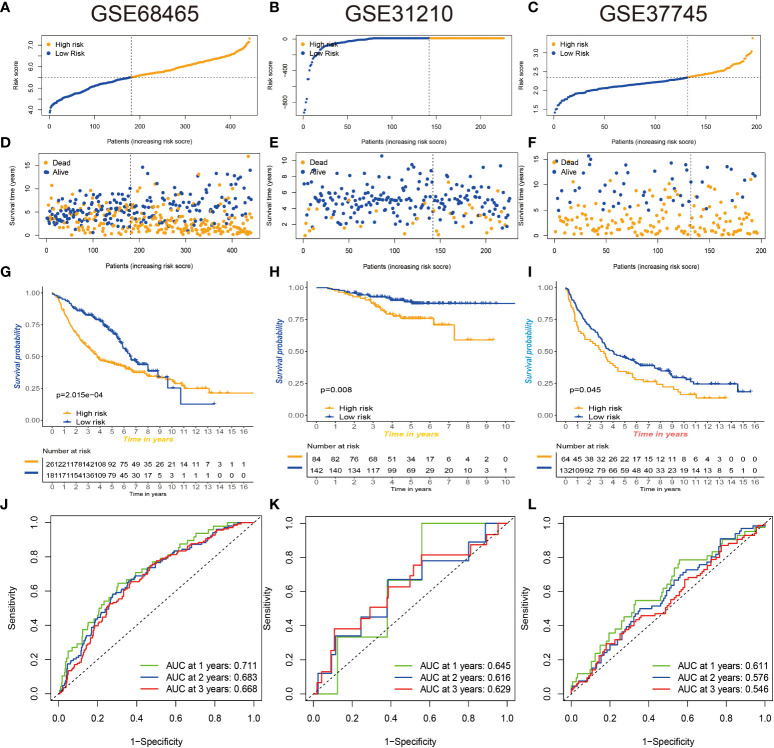
Three GEO databases validate prognostic risk signatures. **(A–F)** Distribution and scatterplot of risk scores in the GSE68465, GSE31210, and GSE37745 datasets. **(G–I)** Kaplan–Meier curves showing the prognostic survival value of the three datasets. **(J–L)** Sensitivity and specificity of ROC curves for predicting 1-, 3-, and 5-year survival in three datasets. GEO, Gene Expression Omnibus; ROC, receiver operating characteristic.

To further verify the stability of our constructed prognostic risk model, three GEO datasets (GSE68465, GSE31210, and GSE37745) were used to construct an independent external validation cohort. We used the same calculation to calculate the risk score power for each patient and found that patients in the low-risk group had better survival values (**Figures 10A–L**). Excellent validation of the prognostic risk model with extremely better accuracy and prognostic value.

### Validation of signature-related genes in clinical samples

We used the public database (TCGA and Genotype-Tissue Expression (GTEx)) and qRT-PCR assays to explore the expression levels of the 13 hub signature-associated genes. As shown in **Supplementary Figures S4A, B**, tumor tissues showed obviously higher expression levels of CCNE1, CCNB2, TNS4, FGA, CDC20, BUB1B, CENPW, TRIP13, TROAP, TMEM163, KRT6A, and CLDN2 than did the normal tissues. Conversely, there was a higher expression of ITGA6 and FOSL1 in normal samples. The abovementioned gene expression results in clinical tissue samples that almost conformed to the public database (**Supplementary Figure S4C**).

## Discussion

Lung cancer is currently the main cause of cancer-related death in the world, with a very high mortality rate (40). However, the clinical symptoms of NSCLC appear late, related examinations have not been popularized, and the prognosis of NSCLC patients is still poor (41). Therefore, we urgently need to analyze the clinical features, survival prognosis, tumor microenvironment, and drug sensitivity of NSCLC to further explore immune prediction efficacy and therapeutic drugs to improve the diagnosis and treatment of NSCLC (42, 43).

Necroptosis is a form of programmed cell death regulated by three major mediators, RIPK1, RIPK3, and MLKL (20), is a key process in cancer biology, including tumor initiation and progression, invasion and migration, and tumor immunosuppression (44, 45). The necroptosis process is initiated by RIPK1, which then combines with RIPK3 to form an oligomeric complex called ‘necrosome’ (46–48). Necrosomes in turn subsequently induce phosphorylation of MLKL for rapid membrane permeabilization, leading to cell rupture and subsequent release of contents, causing damage-associated molecular patterns (DAMPs) of immune responses (49, 50). Throughout the process, the kinase activities of RIPK1 and RIPK3 are critical in the necroptosis pathway (51, 52).

According to scientific research over the years, necroptosis-related genes play a huge role in tumor occurrence and progression, immune prediction, drug sensitivity, and therapeutic value, either alone or by interaction. On the one hand, they can act as a protective mechanism, eliciting specific adaptive immune responses, preventing the occurrence and progression of cancer, and playing a crucial role in eliminating cancer cells. According to related studies, the expression of RIPK3 is absent or significantly reduced in many cancer cell lines (53, 54); for example, compared with normal colorectal mucosal cells, the expression of RIPK3 is significantly reduced in colorectal cancer. High expression of RIPK3 significantly inhibited the expansion of colorectal cancer cells *in vitro*. In addition, several studies have shown that RIPK3 knockout mice have a higher risk of colorectal cancer and produce more pro-inflammatory or tumor-promoting factors (55). Knockout of the RIPK1, RIPK3, or MLKL genes in breast cancer cells significantly reduces the oncogenic activity of these cells and sensitizes the breast cancer cells to radiation therapy. On the other hand, it also promotes the rapid migration and invasion of cancer. For example, colon and esophageal cancer patients have a poorer prognosis and reduced prognostic survival when MLKL phosphorylation levels are elevated, suggesting that necroptosis promotes tumor progression and migration (19). At the same time, low expression of MLKL was also found to be associated with poor prognosis in various gynecological malignancies (56–58). RIPK3/RIPK1 regulation of CXCL1 in pancreatic cancer restricts infiltration of highly immunogenic T or B cells to promote tumor migration and invasion (25). The mechanisms and potential biological functions of necroptosis-related genes in NSCLC have not been specifically explored and elucidated.

In this study, we used consensus clustering analysis to divide the total sample into two clusters based on the mRNA and protein expression levels of 40 necroptosis-related genes. These two distinct clusters have distinct clinical characteristics, survival values, immune landscapes, and pathways. We found that cluster B has a better survival value than cluster A. Clinical features and prognostic analysis revealed that the modification pattern of necroptosis-related genes was significantly associated with the progression of NSCLC. We continued to use ssGSEA to analyze their infiltration abundance in 23 types of immune cells and found that cluster B had a higher level of immune cell infiltration. We performed GO and KEGG enrichment analysis on these necroptosis genes to explore their biological functions and mechanisms of action, and we found that they were highly enriched in cell cycle and p53 signaling pathways. p53 is an important tumor suppressor gene, most frequently altered in human cancers; mutations in this gene are present in 50% of aggressive tumors, and mutated p53 is often used as a target for anticancer therapy (59, 60). Next, we further divided the screened pre-differential genes into three genomic subtypes. Compared with the other two groups, gene cluster B had the best survival advantage. The clinical characteristics and expression levels in necroptosis-related genes showed that the three genomic subtypes were significantly different and played a crucial part in the survival assessment and prognostic value of NSCLC patients. To more accurately quantify and assess the role of necroptosis-related genes in individual NSCLC, we constructed a prognostic risk model. We identified the 14 best prognostic genes for risk assessment and divided patients by median into two risk groups, and we found that the low-risk group had a better survival advantage. We used heatmaps and forest plots to describe their clinical correlation analysis and also constructed ROC curve nomograms to predict survival prognosis in NSCLC. We found that the high-risk group had higher TMB values, and the TMB values of different genotypes were significantly positively correlated with risk scores. In this paper, we evaluated the relationship between tumor microenvironment and risk score and found that they were significantly correlated; these results could suggest that risk score is closely related to the tumor microenvironment and tumor mutational burden. It can be used as a prognostic marker for NSCLC to guide further immune prediction and immunotherapy. We continue to use IC50 analysis to assess drug sensitivity and explore appropriate chemotherapeutic drugs to guide the treatment of NSCLC.

Collectively, we divided necroptosis-related genes into two distinct modification patterns based on the mRNA expression levels of necroptosis-related genes in NSCLC and assessed their clinical features, survival prognosis, immune correlates, and pathways. The differential genes were divided into three genomic subtypes, and the prognostic value, tumor microenvironment, and drug sensitivity to treatment were further analyzed by constructing a prognostic risk model. In addition, the research in this paper has certain limitations, and the potential value of necroptosis needs to be clarified by further research in the future (61, 62).

## Conclusion

Necroptosis-related genes play a crucial part in cancer initiation and progression. In the text, we construct a prognostic risk model. The clinical features, copy number variation, tumor microenvironment, immune cell infiltration, immune prediction, and drug sensitivity were comprehensively assessed. It was found that the necroptosis modification pattern was related to the tumor microenvironment and drug sensitivity. New immunotherapy modalities and chemotherapeutics need to be explored to guide NSCLC treatment. This study opens up a new research avenue for the role of necroptosis in NSCLC and also explores novel potential markers to guide the diagnosis and treatment of NSCLC.

## Data availability statement

The original contributions presented in the study are included in the article/**Supplementary material**. Further inquiries can be directed to the corresponding authors.

## Ethics statement

The studies involving human participants were reviewed and approved by Ethical Committee of Affiliated Hospital of Nantong University (2022-L048). The patients/participants provided their written informed consent to participate in this study. Written informed consent was obtained from the individual(s) for the publication of any potentially identifiable images or data included in this article. The patients/participants provided their written informed consent to participate in this study.

## Author contributions

JiaZ and JW contributed equally to this article. JiaZ and JW conducted a formal analysis and drafted the manuscript. HZ, JilZ, and CF are responsible for project management. TW, MX, and ZS participated in software and data analysis. This article was written, edited, and reviewed by JiaZ and JW. YJ and JC revised the manuscript. All authors read and approved the final manuscript.

## Funding

This project was supported by grants from the National Natural Science Foundation of China (81770266), Department of Human Resources and Social Security of Jiangsu Province (BRA2019232), Basic Science Research of Nantong Science and Technology Plan Project (JC2020063), and Basic Science Research of Nantong Science and Technology Plan Project (JC2021184).

## Conflict of interest

The authors declare that the research was conducted in the absence of any commercial or financial relationships that could be construed as a potential conflict of interest.

## Publisher’s note

All claims expressed in this article are solely those of the authors and do not necessarily represent those of their affiliated organizations, or those of the publisher, the editors and the reviewers. Any product that may be evaluated in this article, or claim that may be made by its manufacturer, is not guaranteed or endorsed by the publisher.

## Supplementary material

Supplementary Figure 1
**(A-AB)** Kaplan-Meier curve analysis of OS rates for necroptosis-related genes.

Supplementary Figure 2
**(A-D)** Consensus clustering of NSCLC patients with k = 2-5.

Supplementary Figure 3
**(A)** Consensus clustering CDF for k = 2-9. **(B)** CDF curve of consensus clustering. **(C)** Trace plots for k = 2 to 9. **(D, E)** Differences in risk scores between genomic subtypes. **(F)** Differences in risk scores between necroptosis subtypes. **(G)** Relationship between the two risk groups and TME scores.

Supplementary Figure 4 Validation of signature-related genes expression. **(A)** Expression of 13 genes in clinical samples from TCGA and GTEx database. **(B)** Expression of 13 genes in clinical samples from our hospital.

## Abbreviations

NSCLC, non-small cell lung cancer; TME, tumor microenvironment; CNV, copy number variation; HR, hazard ratio; GSVA, gene set variation analysis; PCA, principal component analysis; ssGSEA, single-sample gene set enrichment analysis; GO, Gene Ontology; DEGs, differentially expressed genes.
