# Supplementary figures and images for: Identification of molecular subtypes, risk signature, and immune landscape mediated by necroptosis-related genes in non-small cell lung cancer
Source: Front Oncol. 2022 Jul 28;12:955186. doi: 10.3389/fonc.2022.955186 (PMC9367639; doi:10.3389/fonc.2022.955186)

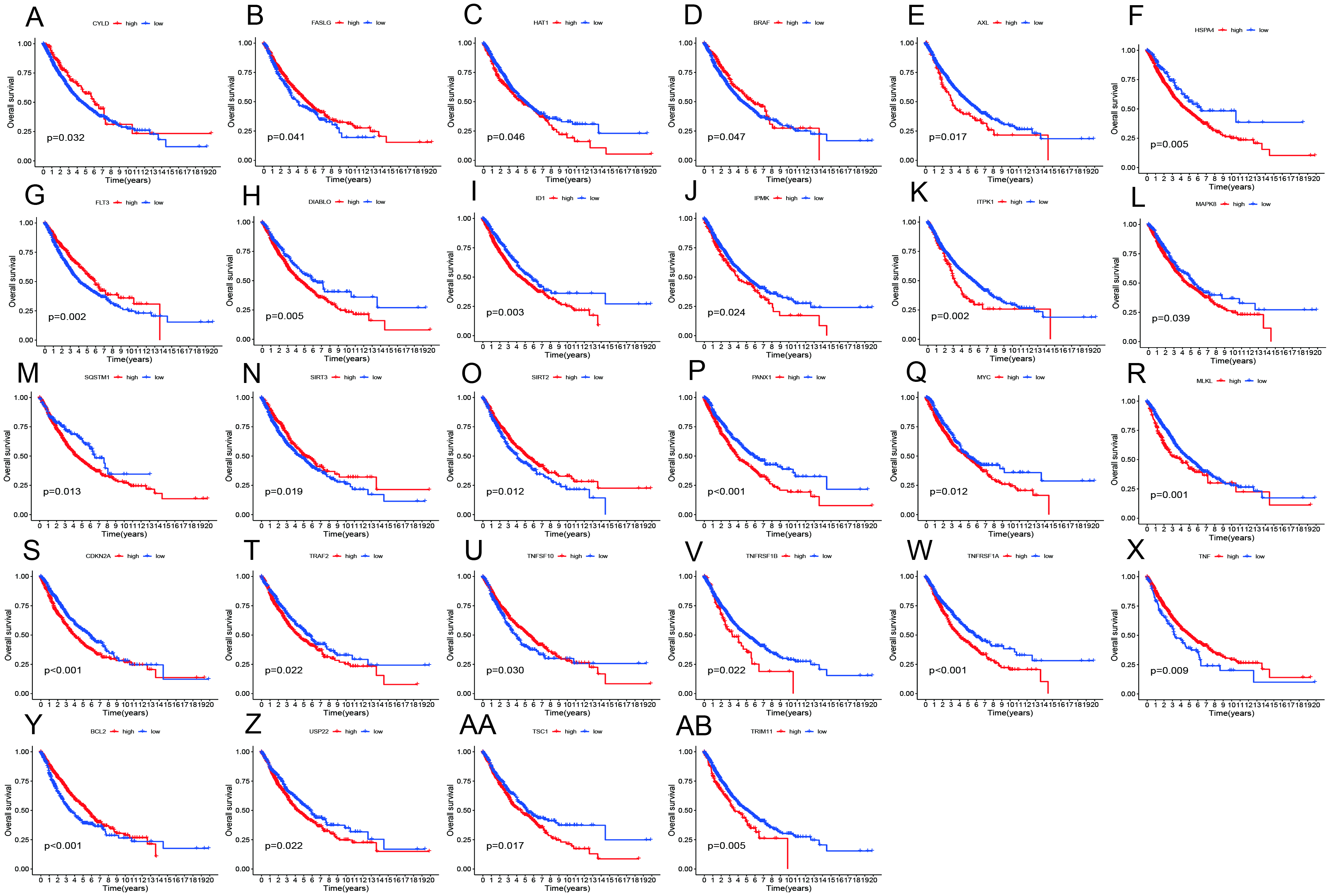

Supplement: Supplementary Figure 1 — (A-AB) Kaplan-Meier curve analysis of OS rates for necroptosis-related genes. [file Image_1.jpeg]

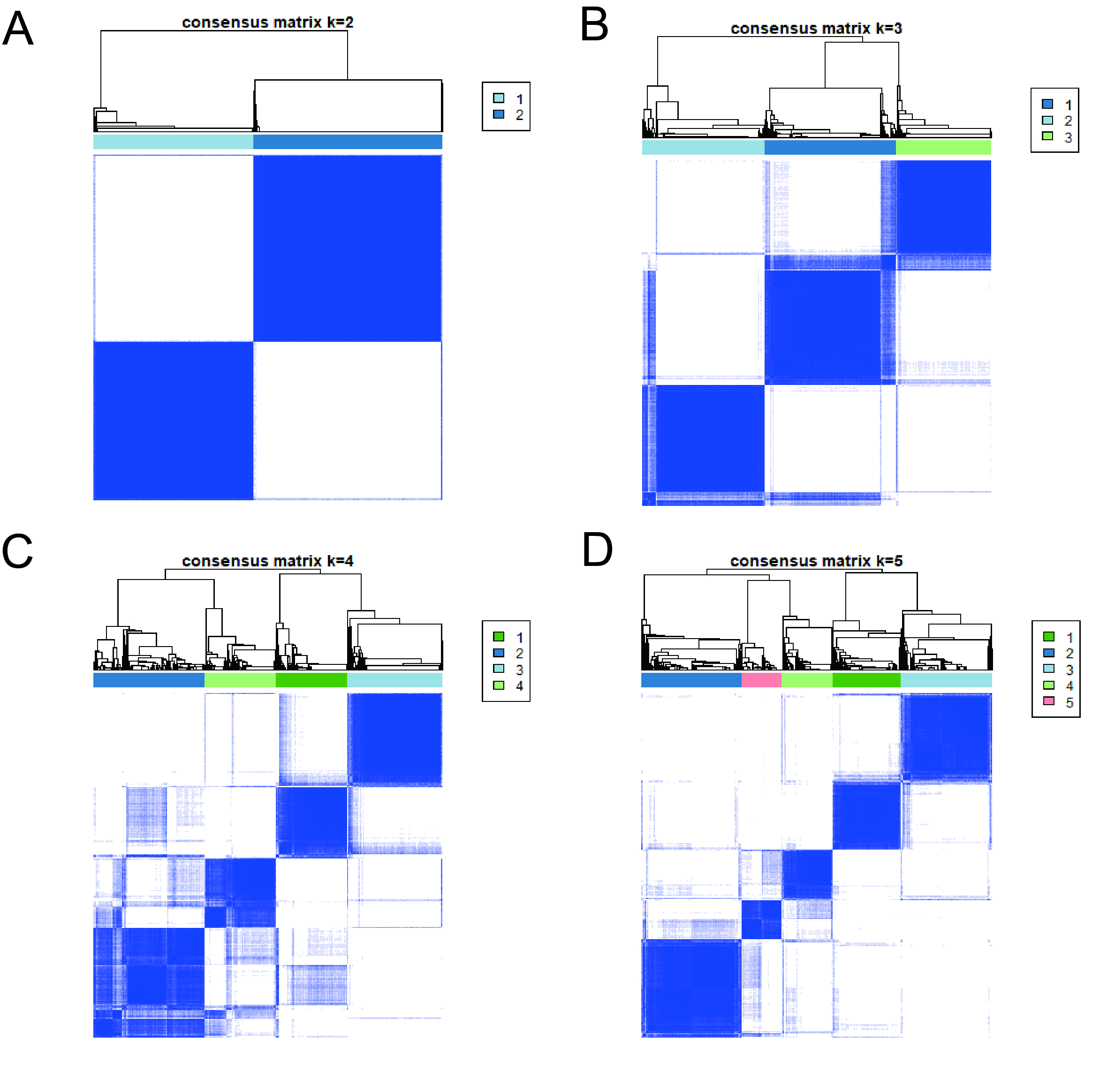

Supplement: Supplementary Figure 2 — (A-D) Consensus clustering of NSCLC patients with k = 2-5. [file Image_2.jpeg]

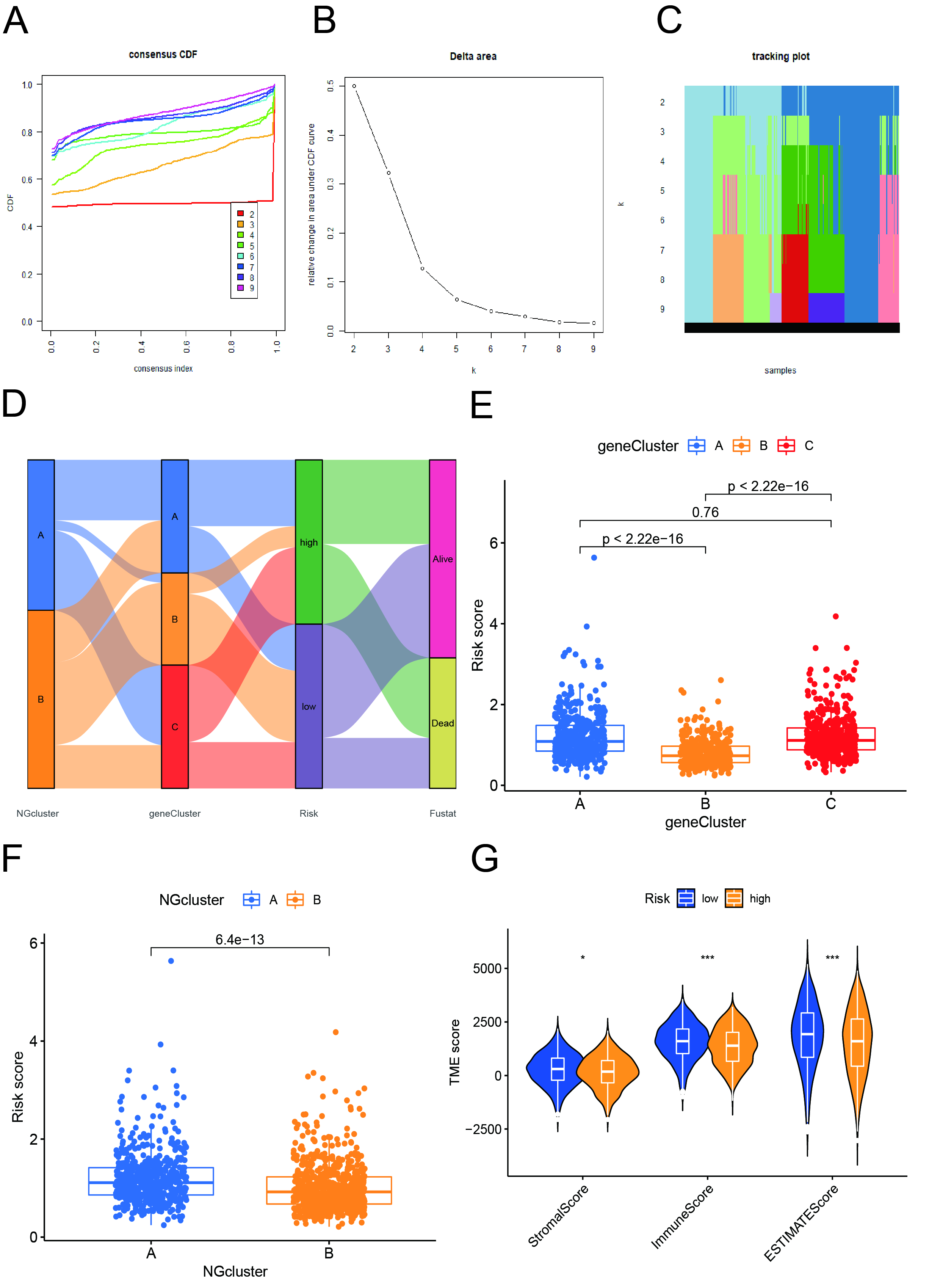

Supplement: Supplementary Figure 3 — (A) Consensus clustering CDF for k = 2-9. (B) CDF curve of consensus clustering. (C) Trace plots for k = 2 to 9. (D, E) Differences in risk scores between genomic subtypes. (F) Differences in risk scores between necroptosis subtypes. (G) Relationship between the two risk groups and TME scores. [file Image_3.jpeg]

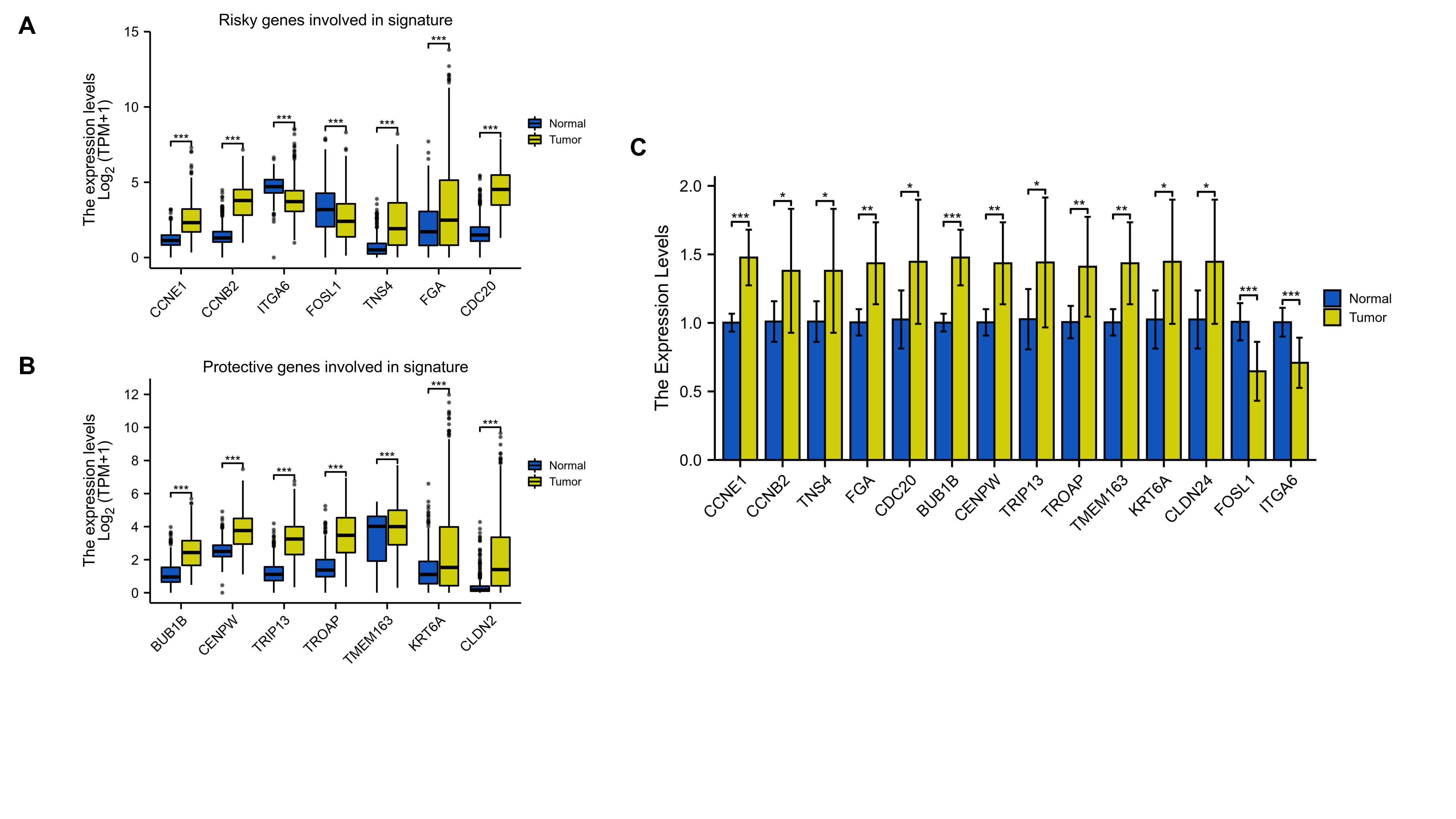

Supplement: Supplementary Figure 4 — Validation of signature-related genes expression. (A) Expression of 13 genes in clinical samples from TCGA and GTEx database. (B) Expression of 13 genes in clinical samples from our hospital. [file Image_4.tiff]
