# Supplementary material for: Identification of molecular subtypes, risk signature, and immune landscape mediated by necroptosis-related genes in non-small cell lung cancer
Source: Front Oncol. 2022 Jul 28;12:955186. doi: 10.3389/fonc.2022.955186 (PMC9367639; doi:10.3389/fonc.2022.955186)
Supplement: Supplementary file 8 [file Table_4.docx]

| **Table-S4: Data set information included in this study for necroptosis classification** | | | | | |
| --- | --- | --- | --- | --- | --- |
| **Accession number** | **Number of NSCLC patients** | **Number of normal patients** | **Number of other patients** | **Platform** | **Platform** |
| TCGA | 1037 | 108 | 0 | / | Illumina RNAseq |
| GEO: GSE50081 | 129 | 0 | 52 | GPL570 | Affymetrix Human Genome U133 Plus 2.0 Array |
| GEO: GSE37745 | 196 | 0 | 0 | GPL570 | Affymetrix Human Genome U133 Plus 2.0 Array |
| GEO: GSE31210 | 226 | 0 | 0 | GPL570 | Affymetrix Human Genome U133 Plus 2.0 Array |
| GEO: GSE68465 | 443 | 19 | 0 | GPL96 | Affymetrix Human Genome U133A Array |
