# Supplementary material for: Identification of molecular subtypes, risk signature, and immune landscape mediated by necroptosis-related genes in non-small cell lung cancer
Source: Front Oncol. 2022 Jul 28;12:955186. doi: 10.3389/fonc.2022.955186 (PMC9367639; doi:10.3389/fonc.2022.955186)
Supplement: Supplementary file 9 [file DataSheet_1.zip › raw data and R code for edior/procedure file/Data sheets 17-18 url.docx]

Nutstore

Data sheet 17

<https://www.jianguoyun.com/p/Da3VQU0Q6fuxChi6l7QE>

Data sheet 18

<https://www.jianguoyun.com/p/DayJ5jwQ6fuxChi9l7QE>
