# Supplementary material for: Identification of molecular subtypes, risk signature, and immune landscape mediated by necroptosis-related genes in non-small cell lung cancer
Source: Front Oncol. 2022 Jul 28;12:955186. doi: 10.3389/fonc.2022.955186 (PMC9367639; doi:10.3389/fonc.2022.955186)
Supplement: Supplementary file 9 [file DataSheet_1.zip › raw data and R code for edior/R code for all figure and table.docx]

1.

use strict;

#use warnings;

my $file=$ARGV[0];

#use Data::Dumper;

use JSON;

my $json = new JSON;

my $js;

my %hash=();

my @normalSamples=();

my @tumorSamples=();

open JFILE, "$file";

while(<JFILE>) {

$js .= "$_";

}

my $obj = $json->decode($js);

for my $i(@{$obj})

{

my $file_name=$i->{'file_name'};

my $file_id=$i->{'file_id'};

my $entity_submitter_id=$i->{'associated_entities'}->[0]->{'entity_submitter_id'};

$file_name=~s/\.gz//g;

if(-f $file_name)

{

my @idArr=split(/\-/,$entity_submitter_id);

if($idArr[3]=~/^0/)

{

push(@tumorSamples,$entity_submitter_id);

}

else

{

push(@normalSamples,$entity_submitter_id);

}

open(RF,"$file_name") or die $!;

while(my $line=<RF>)

{

next if($line=~/^\n/);

next if($line=~/^\_/);

chomp($line);

my @arr=split(/\t/,$line);

${$hash{$arr[0]}}{$entity_submitter_id}=$arr[1];

}

close(RF);

}

}

#print Dumper $obj

open(WF,">mRNAmatrix.txt") or die $!;

my $normalCount=$#normalSamples+1;

my $tumorCount=$#tumorSamples+1;

if($normalCount==0)

{

print WF "id";

}

else

{

print WF "id\t" . join("\t",@normalSamples);

}

print WF "\t" . join("\t",@tumorSamples) . "\n";

foreach my $key(keys %hash)

{

print WF $key;

foreach my $normal(@normalSamples)

{

print WF "\t" . ${$hash{$key}}{$normal};

}

foreach my $tumor(@tumorSamples)

{

print WF "\t" . ${$hash{$key}}{$tumor};

}

print WF "\n";

}

close(WF);

print "normal count: $normalCount\n";

print "tumor count: $tumorCount\n";

2.

use strict;

#use warnings;

my $file=$ARGV[0];

#use Data::Dumper;

use JSON;

my $json = new JSON;

my $js;

my %hash=();

my @normalSamples=();

my @tumorSamples=();

open JFILE, "$file";

while(<JFILE>) {

$js .= "$_";

}

my $obj = $json->decode($js);

for my $i(@{$obj})

{

my $file_name=$i->{'file_name'};

my $file_id=$i->{'file_id'};

my $entity_submitter_id=$i->{'associated_entities'}->[0]->{'entity_submitter_id'};

$file_name=~s/\.gz//g;

if(-f $file_name)

{

my @idArr=split(/\-/,$entity_submitter_id);

if($idArr[3]=~/^0/)

{

push(@tumorSamples,$entity_submitter_id);

}

else

{

push(@normalSamples,$entity_submitter_id);

}

open(RF,"$file_name") or die $!;

while(my $line=<RF>)

{

next if($line=~/^\n/);

next if($line=~/^\_/);

chomp($line);

my @arr=split(/\t/,$line);

${$hash{$arr[0]}}{$entity_submitter_id}=$arr[1];

}

close(RF);

}

}

#print Dumper $obj

open(WF,">mRNAmatrix.txt") or die $!;

my $normalCount=$#normalSamples+1;

my $tumorCount=$#tumorSamples+1;

if($normalCount==0)

{

print WF "id";

}

else

{

print WF "id\t" . join("\t",@normalSamples);

}

print WF "\t" . join("\t",@tumorSamples) . "\n";

foreach my $key(keys %hash)

{

print WF $key;

foreach my $normal(@normalSamples)

{

print WF "\t" . ${$hash{$key}}{$normal};

}

foreach my $tumor(@tumorSamples)

{

print WF "\t" . ${$hash{$key}}{$tumor};

}

print WF "\n";

}

close(WF);

print "normal count: $normalCount\n";

print "tumor count: $tumorCount\n";

3.

use strict;

my $gtfFile="human.gtf";

my $expFile="mRNAmatrix.txt";

my $outFile="symbol.txt";

my %hash=();

open(RF,"$gtfFile") or die $!;

while(my $line=<RF>)

{

chomp($line);

if($line=~/gene_id \"(.+?)\"\;.+gene_name "(.+?)"\;.+gene_biotype \"(.+?)\"\;/)

{

$hash{$1}=$2;

}

}

close(RF);

open(RF,"$expFile") or die $!;

open(WF,">$outFile") or die $!;

while(my $line=<RF>)

{

if($.==1)

{

print WF $line;

next;

}

chomp($line);

my @arr=split(/\t/,$line);

$arr[0]=~s/(.+)\..+/$1/g;

if(exists $hash{$arr[0]})

{

$arr[0]=$hash{$arr[0]};

print WF join("\t",@arr) . "\n";

}

}

close(WF);

close(RF);

4.

use strict;

#use warnings;

use XML::Simple;

opendir(RD, ".") or die $!;

my @dirs=readdir(RD);

closedir(RD);

open(WF,">clinical.xls") or die $!;

print WF "Id\tfutime\tfustat\tAge\tGender\tGrade\tStage\tT\tM\tN\n";

foreach my $dir(@dirs){

#print $dir . "\n";

next if($dir eq '.');

next if($dir eq '..');

#print $dir . "\n";

if(-d $dir){

opendir(RD,"$dir") or die $!;

while(my $xmlfile=readdir(RD)){

if($xmlfile=~/\.xml$/){

#print "$dir\\$xmlfile\n";

my $userxs = XML::Simple->new(KeyAttr => "name");

my $userxml="";

if(-f "$dir/$xmlfile"){

$userxml = $userxs->XMLin("$dir/$xmlfile");

}else{

$userxml = $userxs->XMLin("$dir\$xmlfile");

}

# print output

#open(WF,">dumper.txt") or die $!;

#print WF Dumper($userxml);

#close(WF);

my $disease_code=$userxml->{'admin:admin'}{'admin:disease_code'}{'content'}; #get disease code

my $disease_code_lc=lc($disease_code);

my $patient_key=$disease_code_lc . ':patient'; #ucec:patient

my $follow_key=$disease_code_lc . ':follow_ups';

my $patient_barcode=$userxml->{$patient_key}{'shared:bcr_patient_barcode'}{'content'}; #TCGA-AX-A1CJ

my $gender=$userxml->{$patient_key}{'shared:gender'}{'content'}; #male/female

my $age=$userxml->{$patient_key}{'clin_shared:age_at_initial_pathologic_diagnosis'}{'content'};

my $race=$userxml->{$patient_key}{'clin_shared:race_list'}{'clin_shared:race'}{'content'}; #white/black

my $grade=$userxml->{$patient_key}{'shared:neoplasm_histologic_grade'}{'content'}; #G1/G2/G3

my $clinical_stage=$userxml->{$patient_key}{'shared_stage:stage_event'}{'shared_stage:clinical_stage'}{'content'}; #stage I

my $clinical_T=$userxml->{$patient_key}{'shared_stage:stage_event'}{'shared_stage:tnm_categories'}{'shared_stage:clinical_categories'}{'shared_stage:clinical_T'}{'content'};

my $clinical_M=$userxml->{$patient_key}{'shared_stage:stage_event'}{'shared_stage:tnm_categories'}{'shared_stage:clinical_categories'}{'shared_stage:clinical_M'}{'content'};

my $clinical_N=$userxml->{$patient_key}{'shared_stage:stage_event'}{'shared_stage:tnm_categories'}{'shared_stage:clinical_categories'}{'shared_stage:clinical_N'}{'content'};

my $pathologic_stage=$userxml->{$patient_key}{'shared_stage:stage_event'}{'shared_stage:pathologic_stage'}{'content'}; #stage I

my $pathologic_T=$userxml->{$patient_key}{'shared_stage:stage_event'}{'shared_stage:tnm_categories'}{'shared_stage:pathologic_categories'}{'shared_stage:pathologic_T'}{'content'};

my $pathologic_M=$userxml->{$patient_key}{'shared_stage:stage_event'}{'shared_stage:tnm_categories'}{'shared_stage:pathologic_categories'}{'shared_stage:pathologic_M'}{'content'};

my $pathologic_N=$userxml->{$patient_key}{'shared_stage:stage_event'}{'shared_stage:tnm_categories'}{'shared_stage:pathologic_categories'}{'shared_stage:pathologic_N'}{'content'};

$gender=(defined $gender)?$gender:"unknow";

$age=(defined $age)?$age:"unknow";

$race=(defined $race)?$race:"unknow";

$grade=(defined $grade)?$grade:"unknow";

$clinical_stage=(defined $clinical_stage)?$clinical_stage:"unknow";

$clinical_T=(defined $clinical_T)?$clinical_T:"unknow";

$clinical_M=(defined $clinical_M)?$clinical_M:"unknow";

$clinical_N=(defined $clinical_N)?$clinical_N:"unknow";

$pathologic_stage=(defined $pathologic_stage)?$pathologic_stage:"unknow";

$pathologic_T=(defined $pathologic_T)?$pathologic_T:"unknow";

$pathologic_M=(defined $pathologic_M)?$pathologic_M:"unknow";

$pathologic_N=(defined $pathologic_N)?$pathologic_N:"unknow";

my $survivalTime="";

my $vital_status=$userxml->{$patient_key}{'clin_shared:vital_status'}{'content'};

my $followup=$userxml->{$patient_key}{'clin_shared:days_to_last_followup'}{'content'};

my $death=$userxml->{$patient_key}{'clin_shared:days_to_death'}{'content'};

if($vital_status eq 'Alive'){

$survivalTime="$followup\t0";

}

else{

$survivalTime="$death\t1";

}

for my $i(keys %{$userxml->{$patient_key}{$follow_key}}){

eval{

$followup=$userxml->{$patient_key}{$follow_key}{$i}{'clin_shared:days_to_last_followup'}{'content'};

$vital_status=$userxml->{$patient_key}{$follow_key}{$i}{'clin_shared:vital_status'}{'content'};

$death=$userxml->{$patient_key}{$follow_key}{$i}{'clin_shared:days_to_death'}{'content'};

};

if($@){

for my $j(0..5){ #假设最多有6次随访

my $followup_for=$userxml->{$patient_key}{$follow_key}{$i}[$j]{'clin_shared:days_to_last_followup'}{'content'};

my $vital_status_for=$userxml->{$patient_key}{$follow_key}{$i}[$j]{'clin_shared:vital_status'}{'content'};

my $death_for=$userxml->{$patient_key}{$follow_key}{$i}[$j]{'clin_shared:days_to_death'}{'content'};

if( ($followup_for =~ /\d+/) || ($death_for =~ /\d+/) ){

$followup=$followup_for;

$vital_status=$vital_status_for;

$death=$death_for;

my @survivalArr=split(/\t/,$survivalTime);

if($vital_status eq 'Alive'){

if($followup>$survivalArr[0]){

$survivalTime="$followup\t0";

}

}

else{

if($death>$survivalArr[0]){

$survivalTime="$death\t1";

}

}

}

}

}

my @survivalArr=split(/\t/,$survivalTime);

if($vital_status eq 'Alive'){

if($followup>$survivalArr[0]){

$survivalTime="$followup\t0";

}

}

else{

if($death>$survivalArr[0]){

$survivalTime="$death\t1";

}

}

}

print WF "$patient_barcode\t$survivalTime\t$age\t$gender\t$grade\t$pathologic_stage\t$pathologic_T\t$pathologic_M\t$pathologic_N\n";

}

}

close(RD);

}

}

close(WF);

5.

#if (!requireNamespace("BiocManager", quietly = TRUE))

# install.packages("BiocManager")

#BiocManager::install("limma")

library(limma) #引用包

inputFile="symbol.txt" #输入文件

setwd("C:\\biowolf\\prgTME\\07.FPKM2TPM") #设置工作目录

#读取输入文件,并对输入文件整理

outTab=data.frame()

rt=read.table(inputFile, header=T, sep="\t", check.names=F)

rt=as.matrix(rt)

rownames(rt)=rt[,1]

exp=rt[,2:ncol(rt)]

dimnames=list(rownames(exp),colnames(exp))

data=matrix(as.numeric(as.matrix(exp)),nrow=nrow(exp),dimnames=dimnames)

data=avereps(data)

#FPKM转换为TPM

fpkmToTpm=function(fpkm){

exp(log(fpkm) - log(sum(fpkm)) + log(1e6))

}

tpm=apply(data, 2, fpkmToTpm)

#输出转换结果

tpmOut=rbind(ID=colnames(tpm), tpm)

write.table(tpmOut, file="TCGA.TPM.txt", sep="\t", col.names=F, quote=F)

6.

use strict;

#use warnings;

print STDERR "gene symbol column number: ";

my $geneSymbolCol=<STDIN>;

chomp($geneSymbolCol);

$geneSymbolCol--;

my $expFile="probeMatrix.txt";

my $gplFile="ann.txt";

my $expFileWF="geoMatrix.txt";

my %hash=();

my @sampleName=();

open(EXP,"$expFile") or die $!;

while(my $exp=<EXP>)

{

next if ($exp=~/^(\n|\!)/);

chomp($exp);

if($.==1)

{

my @expArr=split(/\t/,$exp);

for(my $i=0;$i<=$#expArr;$i++)

{

my $singleName=$expArr[$i];

$singleName=~s/\"//g;

if($i==0)

{

push(@sampleName,"ID_REF");

}

else

{

my @singleArr=split(/\_|\./,$singleName);

push(@sampleName,$singleArr[0]);

}

}

}

else

{

my @expArr=split(/\t/,$exp);

for(my $i=0;$i<=$#sampleName;$i++)

{

$expArr[$i]=~s/\"//g;

push(@{$hash{$sampleName[$i]}},$expArr[$i]);

}

}

}

close(EXP);

my %probeGeneHash=();

open(GPL,"$gplFile") or die $!;

while(my $gpl=<GPL>)

{

next if($gpl=~/^(\#|ID|\!|\n)/);

chomp($gpl);

my @gplArr=split(/\t/,$gpl);

if((exists $gplArr[$geneSymbolCol]) && ($gplArr[$geneSymbolCol] ne '') && ($gplArr[$geneSymbolCol] !~ /.+\s+.+/) && ($gplArr[$geneSymbolCol]!~/^\d+|^[a-z]/))

{

$gplArr[$geneSymbolCol]=~s/(.+?)\/\/\/(.+)/$1/g;

$gplArr[$geneSymbolCol]=~s/\"//g;

$probeGeneHash{$gplArr[0]}=$gplArr[$geneSymbolCol];

}

}

close(GPL);

my @probeName=@{$hash{"ID_REF"}};

delete($hash{"ID_REF"});

my %geneListHash=();

my %sampleGeneExpHash=();

foreach my $key (keys %hash)

{

my %geneAveHash=();

my %geneCountHash=();

my %geneSumHash=();

my @valueArr=@{$hash{$key}};

for(my $i=0;$i<=$#probeName;$i++)

{

if(exists $probeGeneHash{$probeName[$i]})

{

my $geneName=$probeGeneHash{$probeName[$i]};

$geneListHash{$geneName}++;

$geneCountHash{$geneName}++;

$geneSumHash{$geneName}+=$valueArr[$i];

}

}

foreach my $countKey (keys %geneCountHash)

{

$geneAveHash{$countKey}=$geneSumHash{$countKey}/$geneCountHash{$countKey};

}

$sampleGeneExpHash{$key}=\%geneAveHash;

}

open(WF,">$expFileWF") or die $!;

$sampleName[0]="geneNames";

print WF join("\t",@sampleName) . "\n";

foreach my $probeGeneValue (sort(keys %geneListHash))

{

if($probeGeneValue eq "KIAA1429"){

print WF "VIRMA\t";

}elsif($probeGeneValue eq "HNRPA2B1"){

print WF "HNRNPA2B1\t";

}elsif($probeGeneValue eq "KIAA0853"){

print WF "ZC3H13\t";

}elsif($probeGeneValue eq "METT10D"){

print WF "METTL16\t";

}else{

print WF $probeGeneValue . "\t";

}

for(my $i=1;$i<$#sampleName;$i++)

{

print WF ${$sampleGeneExpHash{$sampleName[$i]}}{$probeGeneValue} . "\t";

}

my $i=$#sampleName;

print WF ${$sampleGeneExpHash{$sampleName[$i]}}{$probeGeneValue} . "\n";

}

close(WF);

7.

#if (!require("BiocManager"))

# install.packages("BiocManager")

#BiocManager::install("maftools")

library(maftools) #引用包

setwd("D:\\sx\\140prgTME\\11.maftools") #设置工作目录

#读取突变基因文件

geneRT=read.table("gene.txt", header=T, sep="\t", check.names=F, row.names=1)

gene=row.names(geneRT)

#绘制瀑布图

pdf(file="oncoplot.pdf", width=6.5, height=8.5)

maf=read.maf(maf="input.maf")

oncoplot(maf=maf, genes=gene, draw_titv=T)

dev.off()

8.

use strict;

#use warnings;

my $file="input.maf";

my %hash=();

my @sampleArr=();

my %sampleHash=();

my $gene="all";

my %fieldHash=();

my %countHash=();

my $lineCount=0;

open(RF,"$file") or die $!;

while(my $line=<RF>){

next if($line=~/^\n/);

next if($line=~/^\#/);

$lineCount++;

chomp($line);

my @arr=split(/\t/,$line);

if($lineCount==1){

for(my $i=0;$i<=$#arr;$i++){

$fieldHash{$arr[$i]}=$i;

}

next;

}

#去除氨基酸没有改变的

if($arr[$fieldHash{"Amino_acids"}] eq ""){

next;

}

if($arr[$fieldHash{"Variant_Classification"}] eq "Silent"){

next;

}

if($arr[$fieldHash{"Variant_Classification"}] eq "Splice_Region"){

next;

}

my $sampleName=$arr[$fieldHash{"Tumor_Sample_Barcode"}];

my @sampleNameArr=split(/\-/, $sampleName);

my $sample="$sampleNameArr[0]-$sampleNameArr[1]-$sampleNameArr[2]";

#统计每个样品突变位点数目

$countHash{$sample}++;

#构建突变hash

unless($sampleHash{$sample}){

push(@sampleArr,$sample);

$sampleHash{$sample}=1;

}

my $geneField=$fieldHash{"Hugo_Symbol"};

if( ($gene eq "all") || ($gene eq $arr[$geneField]) ){

$hash{$arr[$geneField]}{$sample}=1;

}

}

close(RF);

#输出突变矩阵

my %geneCountHash=();

open(WF,">mutMatrix.txt") or die $!;

print WF "Gene\t" . join("\t",@sampleArr) . "\n";

foreach my $key(keys %hash){

print WF $key;

foreach my $sample(@sampleArr){

if(exists ${$hash{$key}}{$sample}){

print WF "\tMutation";

$geneCountHash{$key}++;

}

else{

print WF "\tWild";

}

}

print WF "\n";

}

close(WF);

#输出每个样品肿瘤突变负荷

open(WF,">TMB.txt") or die $!;

print WF "id\tTMB\n";

foreach my $key (keys %countHash){

my $tmb=$countHash{$key}/38;

print WF "$key\t$tmb\n";

}

close(WF);

#输出每个基因突变count

open(COUNT,">geneMut.txt") or die $!;

print COUNT "Gene\tNum\n";

foreach my $countKey(sort{$geneCountHash{$b}<=>$geneCountHash{$a}} keys(%geneCountHash)){

print COUNT "$countKey\t$geneCountHash{$countKey}\n";

}

close(COUNT);

9.

use strict;

#use warnings;

my $expFile="input.tsv";

my $gtfFile="human.gtf";

my $geneFile="gene.txt";

my $outFile="cnvMatrix.txt";

my %geneHash=();

open(RF, "$geneFile") or die $!;

while(my $line=<RF>){

chomp($line);

my @arr=split(/\t/, $line);

$geneHash{$arr[0]}=1;

}

close(RF);

my %hash=();

open(RF,"$gtfFile") or die $!;

while(my $line=<RF>)

{

chomp($line);

if($line=~/gene_id \"(.+?)\"\;.+gene_name "(.+?)"\;.+gene_biotype \"(.+?)\"\;/){

my $ensembl=$1;

my $gene=$2;

my $biotype=$3;

if(exists $geneHash{$gene}){

$hash{$ensembl}=$gene;

}

}

}

close(RF);

open(RF,"$expFile") or die $!;

open(WF,">$outFile") or die $!;

while(my $line=<RF>)

{

if($.==1){

print WF $line;

next;

}

chomp($line);

my @arr=split(/\t/,$line);

$arr[0]=~s/(.+)\..+/$1/g;

if(exists $hash{$arr[0]})

{

$arr[0]=$hash{$arr[0]};

print WF join("\t",@arr) . "\n";

}

}

close(WF);

close(RF);

10.

inputFile="cnvMatrix.txt" #输入文件

setwd("D:\\sx\\140prgTME\\14.CNVfreq") #设置工作目录

rt=read.table(inputFile, header=T, sep="\t", check.names=F, row.names=1) #读取输入文件

GAIN=rowSums(rt> 0) #获取拷贝数增加的样品数目

LOSS=rowSums(rt< 0) #获取拷贝数缺失的样品数目

GAIN=GAIN/ncol(rt)*100 #计算拷贝数增加的百分率

LOSS=LOSS/ncol(rt)*100 #计算拷贝数缺失的百分率

data=cbind(GAIN, LOSS)

data=data[order(data[,"GAIN"],decreasing = T),]

#绘制图形

data.max = apply(data, 1, max)

pdf(file="CNVfreq.pdf", width=9, height=6)

cex=1.3

par(cex.lab=cex, cex.axis=cex, font.axis=2, las=1, xpd=T)

bar=barplot(data.max, col="black", border=NA,

xlab="", ylab="CNV.frequency(%)", space=1.5,

xaxt="n", ylim=c(0,1.2*max(data.max)))

points(bar,data[,"GAIN"], pch=20, col=4, cex=3)

points(bar,data[,"LOSS"], pch=20, col=5, cex=3)

legend("top", legend=c('GAIN','LOSS'), col=4:5, pch=20, bty="n", cex=2, ncol=2)

par(srt=45)

text(bar, par('usr')[3]-0.2, rownames(data), adj=1, cex=0.7)

dev.off()

11.

use strict;

#use warnings;

my %hash=();

open(RF,"cnvMatrix.txt") or die $!;

while(my $line=<RF>){

chomp($line);

my @arr=split(/\t/, $line);

my $sum=0;

foreach my $i(1..$#arr){

$sum+=$arr[$i];

}

if($sum>0){

$hash{$arr[0]}=1;

}

elsif($sum<0){

$hash{$arr[0]}=-1;

}

else{

$hash{$arr[0]}=0;

}

}

close(RF);

open(RF,"geneRef.txt") or die $!;

open(GENE,">Rcircos.geneLabel.txt") or die $!;

open(SCATTER,">Rcircos.scatter.txt") or die $!;

print SCATTER "chromosome\tstart\tstop\tseg.mean\n";

print GENE "Chromosome\tchromStart\tchromEnd\tGene\n";

while(my $line=<RF>){

next if($.==1);

chomp($line);

my @arr=split(/\t/,$line);

if(exists $hash{$arr[0]}){

print GENE "chr$arr[1]\t$arr[2]\t$arr[3]\t$arr[0]\n";

print SCATTER "chr$arr[1]\t$arr[2]\t$arr[3]\t$hash{$arr[0]}\n";

delete($hash{$arr[0]});

}

}

close(SCATTER);

close(GENE);

close(RF);

12.

#install.packages("RCircos")

library("RCircos") #引用包

setwd("D:\\sx\\140prgTME\\16.Rcircos") #设置工作目录

#初始化圈图

cytoBandIdeogram=read.table("refer.txt", header=T, sep="\t")

chr.exclude <- NULL

cyto.info <- cytoBandIdeogram

tracks.inside <- 5

tracks.outside <- 0

RCircos.Set.Core.Components(cyto.info, chr.exclude, tracks.inside, tracks.outside)

#设置圈图参数

rcircos.params <- RCircos.Get.Plot.Parameters()

rcircos.params$text.size=0.8

rcircos.params$point.size=5

RCircos.Reset.Plot.Parameters(rcircos.params)

#输出文件

pdf(file="RCircos.pdf", width=8, height=8)

RCircos.Set.Plot.Area()

RCircos.Chromosome.Ideogram.Plot()

#读取拷贝数的文件，绘制散点图

RCircos.Scatter.Data=read.table("Rcircos.scatter.txt", header=T, sep="\t", check.names=F)

data.col <- 4

track.num <- 1

side <- "in"

RCircos.Scatter.Plot(RCircos.Scatter.Data, data.col, track.num, side, by.fold=0.1)

#读取基因注释文件，标注基因的名称

RCircos.Gene.Label.Data=read.table("Rcircos.geneLabel.txt", header=T, sep="\t", check.names=F)

name.col <- 4

side <- "in"

track.num <- 2

RCircos.Gene.Connector.Plot(RCircos.Gene.Label.Data, track.num, side)

track.num <- 3

RCircos.Gene.Name.Plot(RCircos.Gene.Label.Data, name.col, track.num, side)

dev.off()

13.

#if (!requireNamespace("BiocManager", quietly = TRUE))

# install.packages("BiocManager")

#BiocManager::install("limma")

#install.packages("reshape2")

#install.packages("ggpubr")

#引用包

library(limma)

library(reshape2)

library(ggpubr)

expFile="TCGA.TPM.txt" #表达数据矩阵

geneFile="gene.txt" #基因列表

setwd("D:\\sx\\140prgTME\\17.PRGdiff") #设置工作目录

#读取输入文件

rt=read.table(expFile, header=T, sep="\t", check.names=F)

rt=as.matrix(rt)

rownames(rt)=rt[,1]

exp=rt[,2:ncol(rt)]

dimnames=list(rownames(exp), colnames(exp))

data=matrix(as.numeric(as.matrix(exp)), nrow=nrow(exp), dimnames=dimnames)

data=avereps(data)

#提取细胞焦亡基因的表达量

gene=read.table(geneFile, header=T, sep="\t", check.names=F)

sameGene=intersect(as.vector(gene[,1]), row.names(data))

data=data[sameGene,]

#正常和肿瘤数目

group=sapply(strsplit(colnames(data),"\\-"), "[", 4)

group=sapply(strsplit(group,""), "[", 1)

group=gsub("2", "1", group)

conNum=length(group[group==1]) #正常组样品数目

treatNum=length(group[group==0]) #肿瘤组样品数目

sampleType=c(rep(1,conNum), rep(2,treatNum))

#提取显著差异的基因

exp=log2(data+1)

exp=as.data.frame(t(exp))

exp=cbind(exp, Type=sampleType)

exp$Type=ifelse(exp$Type==1, "Normal", "Tumor")

sigGene=c()

for(i in colnames(exp)[1:(ncol(exp)-1)]){

if(sd(exp[,i])<0.001){next}

wilcoxTest=wilcox.test(exp[,i] ~ exp[,"Type"])

pvalue=wilcoxTest$p.value

if(wilcoxTest$p.value<0.05){

sigGene=c(sigGene, i)

}

}

sigGene=c(sigGene, "Type")

exp=exp[,sigGene]

#把数据转换成ggplot2输入文件

data=melt(exp, id.vars=c("Type"))

colnames(data)=c("Type", "Gene", "Expression")

#绘制箱线图

p=ggboxplot(data, x="Gene", y="Expression", color = "Type",

ylab="Gene expression",

xlab="",

legend.title="Type",

palette = c("blue", "red"),

width=1)

p=p+rotate_x_text(60)

p1=p+stat_compare_means(aes(group=Type),

method="wilcox.test",

symnum.args=list(cutpoints = c(0, 0.001, 0.01, 0.05, 1), symbols = c("***", "**", "*", " ")),

label = "p.signif")

#输出箱线图

pdf(file="boxplot.pdf", width=9, height=6)

print(p1)

dev.off()

14.

#if (!requireNamespace("BiocManager", quietly = TRUE))

# install.packages("BiocManager")

#BiocManager::install("limma")

#if (!requireNamespace("BiocManager", quietly = TRUE))

# install.packages("BiocManager")

#BiocManager::install("sva")

#引用包

library(limma)

library(sva)

setwd("D:\\sx\\140prgTME\\18.merge") #设置工作目录

files=c("TCGA.TPM.txt", "GSE50081.txt") #输入文件名称

#获取交集基因

geneList=list()

for(i in 1:length(files)){

inputFile=files[i]

rt=read.table(inputFile, header=T, sep="\t",check.names=F)

header=unlist(strsplit(inputFile, "\\.|\\-"))

geneList[[header[1]]]=as.vector(rt[,1])

}

intersectGenes=Reduce(intersect, geneList)

#数据合并

allTab=data.frame()

batchType=c()

for(i in 1:length(files)){

inputFile=files[i]

header=unlist(strsplit(inputFile, "\\.|\\-"))

#读取输入文件，并对输入文件进行整理

rt=read.table(inputFile, header=T, sep="\t", check.names=F)

rt=as.matrix(rt)

rownames(rt)=rt[,1]

exp=rt[,2:ncol(rt)]

dimnames=list(rownames(exp),colnames(exp))

data=matrix(as.numeric(as.matrix(exp)),nrow=nrow(exp),dimnames=dimnames)

rt=avereps(data)

colnames(rt)=paste0(header[1], "_", colnames(rt))

#对TCGA删除正常样品

if(header[1] == "TCGA"){

group=sapply(strsplit(colnames(rt),"\\-"), "[", 4)

group=sapply(strsplit(group,""), "[", 1)

rt=rt[,group==0]

rt=t(rt)

row.names(rt)=gsub("(.*?)\\-(.*?)\\-(.*?)\\-.*", "\\1\\-\\2\\-\\3", row.names(rt))

rt=avereps(rt)

rt=t(rt)

}

#对数值大的数据取log2

qx=as.numeric(quantile(rt, c(0, 0.25, 0.5, 0.75, 0.99, 1.0), na.rm=T))

LogC=( (qx[5]>100) || ( (qx[6]-qx[1])>50 && qx[2]>0) )

if(LogC){

rt[rt<0]=0

rt=log2(rt+1)}

if(header[1] != "TCGA"){

rt=normalizeBetweenArrays(rt)

}

#数据合并

if(i==1){

allTab=rt[intersectGenes,]

}else{

allTab=cbind(allTab, rt[intersectGenes,])

}

batchType=c(batchType, rep(i,ncol(rt)))

}

#对数据进行批次矫正，输出矫正后的结果

outTab=ComBat(allTab, batchType, par.prior=TRUE)

outTab=rbind(geneNames=colnames(outTab), outTab)

write.table(outTab, file="merge.txt", sep="\t", quote=F, col.names=F)

15.

#if (!requireNamespace("BiocManager", quietly = TRUE))

# install.packages("BiocManager")

#BiocManager::install("limma")

library(limma) #引用包

expFile="merge.txt" #表达数据文件

geneFile="gene.txt" #基因列表文件

setwd("D:\\sx\\140prgTME\\19.prgExp") #设置工作目录

#读取输入文件，并对数据进行处理

rt=read.table(expFile, header=T, sep="\t", check.names=F)

rt=as.matrix(rt)

rownames(rt)=rt[,1]

exp=rt[,2:ncol(rt)]

dimnames=list(rownames(exp),colnames(exp))

data=matrix(as.numeric(as.matrix(exp)),nrow=nrow(exp),dimnames=dimnames)

data=avereps(data)

data=data[rowMeans(data)>0,]

#获取细胞焦亡基因的表达量

gene=read.table(geneFile, header=T, sep="\t", check.names=F)

sameGene=intersect(as.vector(gene[,1]), rownames(data))

geneExp=data[sameGene,]

#输出结果

out=rbind(ID=colnames(geneExp),geneExp)

write.table(out,file="prgGeneExp.txt",sep="\t",quote=F,col.names=F)

16．

#if (!requireNamespace("BiocManager", quietly = TRUE))

# install.packages("BiocManager")

#BiocManager::install("limma")

#install.packages('survival')

#install.packages("survminer")

#引用包

library(limma)

library(survival)

library(survminer)

expFile="prgGeneExp.txt" #表达数据文件

cliFile="time.txt" #生存数据文件

setwd("D:\\sx\\140prgTME\\20.prgSur") #设置工作目录

#读取表达文件，并对输入文件整理

rt=read.table(expFile, header=T, sep="\t", check.names=F)

rt=as.matrix(rt)

rownames(rt)=rt[,1]

exp=rt[,2:ncol(rt)]

dimnames=list(rownames(exp), colnames(exp))

data=matrix(as.numeric(as.matrix(exp)), nrow=nrow(exp), dimnames=dimnames)

data=avereps(data)

data=data[rowMeans(data)>0,]

data=t(data)

rownames(data)=gsub("(.*?)\\_(.*?)", "\\2", rownames(data))

#读取生存数据

cli=read.table(cliFile, header=T, sep="\t", check.names=F, row.names=1)

cli$futime=cli$futime/365

#数据合并

sameSample=intersect(row.names(data), row.names(cli))

data=data[sameSample,]

cli=cli[sameSample,]

rt=cbind(cli, data)

#对基因进行循环，找出预后相关的基因

outTab=data.frame()

km=c()

for(i in colnames(rt[,3:ncol(rt)])){

#cox分析

cox <- coxph(Surv(futime, fustat) ~ rt[,i], data = rt)

coxSummary = summary(cox)

coxP=coxSummary$coefficients[,"Pr(>|z|)"]

outTab=rbind(outTab,

cbind(id=i,

HR=coxSummary$conf.int[,"exp(coef)"],

HR.95L=coxSummary$conf.int[,"lower .95"],

HR.95H=coxSummary$conf.int[,"upper .95"],

pvalue=coxSummary$coefficients[,"Pr(>|z|)"])

)

#km分析

data=rt[,c("futime", "fustat", i)]

colnames(data)=c("futime", "fustat", "gene")

#获取最优cutoff

res.cut=surv_cutpoint(data, time = "futime", event = "fustat", variables =c("gene"))

res.cat=surv_categorize(res.cut)

fit=survfit(Surv(futime, fustat) ~gene, data = res.cat)

#print(paste0(i, " ", res.cut$cutpoint[1]))

#比较高低表达生存差异

diff=survdiff(Surv(futime, fustat) ~gene,data =res.cat)

pValue=1-pchisq(diff$chisq, df=1)

km=c(km, pValue)

#对pvalue<0.05的基因绘制生存曲线

if(pValue<0.05){

if(pValue<0.001){

pValue="p<0.001"

}else{

pValue=paste0("p=",sprintf("%.03f",pValue))

}

#绘制生存曲线

surPlot=ggsurvplot(fit,

data=res.cat,

pval=pValue,

pval.size=6,

legend.title=i,

legend.labs=c("high","low"),

xlab="Time(years)",

ylab="Overall survival",

palette=c("red", "blue"),

break.time.by=1,

conf.int=F,

risk.table=F,

risk.table.title="",

risk.table.height=.25)

pdf(file=paste0("sur.", i, ".pdf"),onefile = FALSE,

width = 5, #图片的宽度

height =4.5) #图片的高度

print(surPlot)

dev.off()

}

}

#输出单因素的结果

outTab=cbind(outTab, km)

write.table(outTab,file="uniCox.txt",sep="\t",row.names=F,quote=F)

17.

#install.packages("igraph")

#install.packages("psych")

#install.packages("reshape2")

#install.packages("RColorBrewer")

#引用包

library(igraph)

library(psych)

library(reshape2)

library(RColorBrewer)

GeneExpfile <- "prgGeneExp.txt" #表达数据文件

Genefile <- "gene.txt" #基因列表文件

Coxfile <- "uniCox.txt" #单因素的结果文件

setwd("D:\\sx\\140prgTME\\21.network") #设置工作目录

#读取输入文件

gene.group <- read.table(Genefile,header=T,sep="\t")

gene.exp <- read.table(GeneExpfile,header=T,sep="\t",row.names=1)

gene.cox <- read.table(Coxfile,header=T,sep="\t")

#基因取交集

colnames(gene.group) <- c('id','group')

genelist <- intersect(gene.group$id, gene.cox$id)

genelist <- intersect(genelist, rownames(gene.exp))

gene.group <- gene.group[match(genelist,gene.group$id),]

gene.group <- gene.group[order(gene.group$group),]

gene.exp <- gene.exp[match(gene.group$id,rownames(gene.exp)),]

gene.cox <- gene.cox[match(gene.group$id,gene.cox$id),]

#准备网络文件

gene.cor <- corr.test(t(gene.exp))

gene.cor.cor <- gene.cor$r

gene.cor.pvalue <- gene.cor$p

gene.cor.cor[upper.tri(gene.cor.cor)] = NA

gene.cor.pvalue[upper.tri(gene.cor.pvalue)] = NA

gene.cor.cor.melt <- melt(gene.cor.cor) #gene1 \t gene2 \t cor

gene.cor.pvalue.melt <- melt(gene.cor.pvalue)

gene.melt <- data.frame(from = gene.cor.cor.melt$Var2,to=gene.cor.cor.melt$Var1,cor=gene.cor.cor.melt$value,pvalue=gene.cor.pvalue.melt$value)

gene.melt <- gene.melt[gene.melt$from!=gene.melt$to&!is.na(gene.melt$pvalue),,drop=F]

gene.edge <- gene.melt[gene.melt$pvalue<0.0001,,drop=F]

gene.edge$color <- ifelse(gene.edge$cor>0,'pink','#6495ED')

gene.edge$weight <- abs(gene.edge$cor)*6

#准备节点属性属性文件

gene.node <- gene.group

group.color <- colorRampPalette(brewer.pal(9, "Set1"))(length(unique(gene.node$group)))

gene.node$color <- group.color[as.numeric(as.factor(gene.node$group))]

gene.node$shape <- "circle"

gene.node$frame <- ifelse(gene.cox$HR>1,'purple',"green")

gene.node$pvalue <- gene.cox$pvalue

# pvalue size

pvalue.breaks <- c(0,0.0001,0.001,0.01,0.05,1)

pvalue.size <- c(16,14,12,10,8)

cutpvalue <- cut(gene.node$pvalue,breaks=pvalue.breaks)

gene.node$size <- pvalue.size[as.numeric(cutpvalue)]

nodefile <- "network.node.txt"

edgefile <- "network.edge.txt"

write.table(gene.node, nodefile, sep="\t", col.names=T, row.names=F, quote=F)

write.table(gene.edge, edgefile, sep="\t", col.names=T, row.names=F, quote=F)

#绘制网络图

node = read.table(nodefile, header=T, sep="\t", comment.char="")

edge = read.table(edgefile, header=T, sep="\t", comment.char="")

g = graph.data.frame(edge,directed = FALSE)

node = node[match(names(components(g)$membership),node$id),]

if(!is.na(match('color',colnames(node)))) V(g)$color = node$color

if(!is.na(match('size',colnames(node)))) V(g)$size = node$size

if(!is.na(match('shape',colnames(node)))) V(g)$shape = node$shape

if(!is.na(match('frame',colnames(node)))) V(g)$frame = node$frame

#输出文件

pdf(file="network.pdf", width=11, height=8)

par(mar=c(0,0,0,0))

layout(matrix(c(1,1,4,2,3,4),nc=2),height=c(4,4,2),width=c(8,3))

#节点坐标

coord = layout_in_circle(g)

degree.x = acos(coord[,1])

degree.y = asin(coord[,2])

degree.alpha = c()

for(i in 1:length(degree.x)){

if(degree.y[i]<0) degree.alpha=c(degree.alpha,2*pi-degree.x[i]) else degree.alpha=c(degree.alpha,degree.x[i])

}

degree.cut.group = (0:8)/4*pi

degree.cut.group[1] = -0.0001

degree.cut = cut(degree.alpha,degree.cut.group)

degree.degree = c(-pi/4,-pi/4,-pi/2,-pi/2,pi/2,pi/2,pi/2,pi/4)

degree = degree.degree[as.numeric(degree.cut)]

#定义饼图,左半圆颜色代表基因的属性,右半圆代表基因的风险,哪些基因是高风险基因,还是低风险基因

values <- lapply(node$id,function(x)c(1,1))

V(g)$pie.color = lapply(1:nrow(node),function(x)c(node$color[x],node$frame[x]))

V(g)$frame = NA

#绘制图形

plot(g,layout=layout_in_circle,vertex.shape="pie",vertex.pie=values,

vertex.label.cex=V(g)$lable.cex,edge.width = E(g)$weight,edge.arrow.size=0,

vertex.label.color=V(g)$color,vertex.frame.color=V(g)$frame,edge.color=E(g)$color,

vertex.label.cex=2,vertex.label.font=2,vertex.size=V(g)$size,edge.curved=0.4,

vertex.color=V(g)$color,vertex.label.dist=1,vertex.label.degree=degree)

# label.degree : zero means to the right; and pi means to the left; up is -pi/2 and down is pi/2; The default value is -pi/4

# label.dist If it is 0 then the label is centered on the vertex; If it is 1 then the label is displayed beside the vertex.

#绘制节点属性图例(基因的属性)

par(mar=c(0,0,0,0))

plot(1,type="n",xlab="",ylab="",axes=F)

groupinfo = unique(data.frame(group=node$group,color=node$color))

legend("left",legend=groupinfo$group,col=groupinfo$color,pch=16,bty="n",cex=3)

#绘制基因风险的图例(哪些基因是高风险的基因,哪些基因是低风险的基因)

par(mar=c(0,0,0,0))

plot(1,type="n",xlab="",ylab="",axes=F)

legend("left",legend=c('Risk factors','Favorable factors'),col=c('purple','green'),pch=16,bty="n",cex=2.5)

#绘制预后pvalue图例

par(mar=c(0,0,0,0))

plot(1,type="n",xlab="",axes=F,ylab="")

legend("top",legend=c('Postive correlation with P<0.0001','Negative correlation with P<0.0001'),lty=1,lwd=4,col=c('pink','#6495ED'),bty="n",cex=2.2)

legend('bottom',legend=c(0.0001,0.001,0.01,0.05,1),pch=16,pt.cex=c(1.6,1.4,1.2,1,0.8)*6,bty="n",ncol=5,cex=2.2,col="black",title="Cox test, pvalue")

dev.off()

18.

#if (!requireNamespace("BiocManager", quietly = TRUE))

# install.packages("BiocManager")

#BiocManager::install("ConsensusClusterPlus")

library(ConsensusClusterPlus) #引用包

expFile="prgGeneExp.txt" #表达数据文件

workDir="D:\\sx\\140prgTME\\22.prgCluster" #工作目录

setwd(workDir) #设置工作目录

#读取输入文件

data=read.table(expFile, header=T, sep="\t", check.names=F, row.names=1)

data=as.matrix(data)

#对样品进行聚类

maxK=9

results=ConsensusClusterPlus(data,

maxK=maxK,

reps=50,

pItem=0.8,

pFeature=1,

title=workDir,

clusterAlg="km",

distance="euclidean",

seed=123456,

plot="png")

#输出分型结果

clusterNum=2 #分成几个亚型

cluster=results[[clusterNum]][["consensusClass"]]

cluster=as.data.frame(cluster)

colnames(cluster)=c("PRGcluster")

letter=c("A","B","C","D","E","F","G")

uniqClu=levels(factor(cluster$PRGcluster))

cluster$PRGcluster=letter[match(cluster$PRGcluster, uniqClu)]

clusterOut=rbind(ID=colnames(cluster), cluster)

write.table(clusterOut, file="PRGcluster.txt", sep="\t", quote=F, col.names=F)

19.

#install.packages("survival")

#install.packages("survminer")

#引用包

library(survival)

library(survminer)

clusterFile="PRGcluster.txt" #分型的结果文件

cliFile="time.txt" #生存数据文件

setwd("D:\\sx\\140prgTME\\23.PRGclusterSur") #设置工作目录

#读取输入文件

cluster=read.table(clusterFile, header=T, sep="\t", check.names=F, row.names=1)

rownames(cluster)=gsub("(.*?)\\_(.*?)", "\\2", rownames(cluster))

cli=read.table(cliFile, header=T, sep="\t", check.names=F, row.names=1)

colnames(cli)=c("futime", "fustat")

cli$futime=cli$futime/365

#数据合并

sameSample=intersect(row.names(cluster), row.names(cli))

rt=cbind(cli[sameSample,,drop=F], cluster[sameSample,,drop=F])

#生存差异统计

length=length(levels(factor(rt$PRGcluster)))

diff=survdiff(Surv(futime, fustat) ~ PRGcluster, data = rt)

pValue=1-pchisq(diff$chisq, df=length-1)

if(pValue<0.001){

pValue="p<0.001"

}else{

pValue=paste0("p=",sprintf("%.03f",pValue))

}

fit <- survfit(Surv(futime, fustat) ~ PRGcluster, data = rt)

#print(surv_median(fit))

#绘制生存曲线

bioCol=c("#0066FF","#FF9900","#FF0000","#6E568C","#7CC767","#223D6C","#D20A13","#FFD121","#088247","#11AA4D")

bioCol=bioCol[1:length]

surPlot=ggsurvplot(fit,

data=rt,

conf.int=F,

pval=pValue,

pval.size=6,

legend.title="PRGcluster",

legend.labs=levels(factor(rt[,"PRGcluster"])),

legend = c(0.8, 0.8),

font.legend=10,

xlab="Time(years)",

break.time.by = 1,

palette = bioCol,

surv.median.line = "hv",

risk.table=T,

cumevents=F,

risk.table.height=.25)

pdf(file="survival.pdf",onefile = FALSE,width=7,height=5.5)

print(surPlot)

dev.off()

20.

#install.packages("pheatmap")

library(pheatmap) #引用包

expFile="prgGeneExp.txt" #表达输入文件

clusterFile="PRGcluster.txt" #分型的结果文件

cliFile="clinical.txt" #临床数据文件

setwd("D:\\sx\\140prgTME\\24.heatmap") #设置工作目录

#读取输入文件

exp=read.table(expFile, header=T, sep="\t", check.names=F, row.names=1)

exp=t(exp)

cluster=read.table(clusterFile, header=T, sep="\t", check.names=F, row.names=1)

#合并表达和分型数据

sameSample=intersect(row.names(exp), row.names(cluster))

exp=exp[sameSample, , drop=F]

cluster=cluster[sameSample, , drop=F]

expCluster=cbind(exp, cluster)

Project=gsub("(.*?)\\_.*", "\\1", rownames(expCluster))

rownames(expCluster)=gsub("(.*?)\\_(.*?)", "\\2", rownames(expCluster))

expCluster=cbind(expCluster, Project)

#合并临床数据

cli=read.table(cliFile, header=T, sep="\t", check.names=F, row.names=1)

cli[,"Age"]=ifelse(cli[,"Age"]=="unknow", "unknow", ifelse(cli[,"Age"]>65,">65","<=65"))

sameSample=intersect(row.names(expCluster), row.names(cli))

expCluster=expCluster[sameSample,,drop=F]

cli=cli[sameSample,,drop=F]

data=cbind(expCluster, cli)

#提取热图数据

data=data[order(data$PRGcluster),]

Type=data[,((ncol(exp)+1):ncol(data))]

data=t(data[,1:ncol(exp)])

#聚类颜色

bioCol=c("#0066FF","#FF9900","#FF0000","#6E568C","#7CC767","#223D6C","#D20A13","#FFD121","#088247","#11AA4D")

ann_colors=list()

prgCluCol=bioCol[1:length(levels(factor(Type$PRGcluster)))]

names(prgCluCol)=levels(factor(Type$PRGcluster))

ann_colors[["PRGcluster"]]=prgCluCol

#热图可视化

pdf("heatmap.pdf", width=7.5, height=5)

pheatmap(data,

annotation=Type,

annotation_colors = ann_colors,

color = colorRampPalette(c(rep("blue",5), "white", rep("orange",5)))(100),

cluster_cols =F,

cluster_rows =F,

scale="row",

show_colnames=F,

fontsize=6,

fontsize_row=6,

fontsize_col=6)

dev.off()

21.

#if (!requireNamespace("BiocManager", quietly = TRUE))

# install.packages("BiocManager")

#BiocManager::install("limma")

#BiocManager::install("GSEABase")

#BiocManager::install("GSVA")

#install.packages("pheatmap")

#引用包

library(limma)

library(GSEABase)

library(GSVA)

library(pheatmap)

expFile="merge.txt" #表达数据文件

clusterFile="PRGcluster.txt" #分型结果文件

gmtFile="c2.cp.kegg.v7.4.symbols.gmt" #基因集文件

setwd("D:\\sx\\140prgTME\\25.GSVA") #设置工作目录

#读取表达输入文件,并对输入文件整理

rt=read.table(expFile, header=T, sep="\t", check.names=F)

rt=as.matrix(rt)

rownames(rt)=rt[,1]

exp=rt[,2:ncol(rt)]

dimnames=list(rownames(exp), colnames(exp))

data=matrix(as.numeric(as.matrix(exp)), nrow=nrow(exp), dimnames=dimnames)

data=avereps(data)

#GSVA分析

geneSets=getGmt(gmtFile, geneIdType=SymbolIdentifier())

gsvaResult=gsva(data,

geneSets,

min.sz=10,

max.sz=500,

verbose=TRUE,

parallel.sz=1)

gsvaOut=rbind(id=colnames(gsvaResult), gsvaResult)

write.table(gsvaOut, file="gsvaOut.txt", sep="\t", quote=F, col.names=F)

#读取cluster文件

cluster=read.table(clusterFile, header=T, sep="\t", check.names=F, row.names=1)

#数据合并

gsvaResult=t(gsvaResult)

sameSample=intersect(row.names(gsvaResult), row.names(cluster))

gsvaResult=gsvaResult[sameSample,,drop=F]

cluster=cluster[sameSample,,drop=F]

gsvaCluster=cbind(gsvaResult, cluster)

Project=gsub("(.*?)\\_.*", "\\1", rownames(gsvaCluster))

gsvaCluster=cbind(gsvaCluster, Project)

#差异分析

adj.P.Val.Filter=0.05

allType=as.vector(gsvaCluster$PRGcluster)

comp=combn(levels(factor(allType)), 2)

for(i in 1:ncol(comp)){

#样品分组

treat=gsvaCluster[gsvaCluster$PRGcluster==comp[2,i],]

con=gsvaCluster[gsvaCluster$PRGcluster==comp[1,i],]

data=rbind(con, treat)

#对通路进行差异分析

Type=as.vector(data$PRGcluster)

ann=data[,c(ncol(data), (ncol(data)-1))]

data=t(data[,-c((ncol(data)-1), ncol(data))])

design=model.matrix(~0+factor(Type))

colnames(design)=levels(factor(Type))

fit=lmFit(data, design)

contrast=paste0(comp[2,i], "-", comp[1,i])

cont.matrix=makeContrasts(contrast, levels=design)

fit2=contrasts.fit(fit, cont.matrix)

fit2=eBayes(fit2)

#输出所有通路的差异情况

allDiff=topTable(fit2,adjust='fdr',number=200000)

allDiffOut=rbind(id=colnames(allDiff),allDiff)

write.table(allDiffOut, file=paste0(contrast, ".all.txt"), sep="\t", quote=F, col.names=F)

#输出显著的差异

diffSig=allDiff[with(allDiff, (abs(logFC)>0.1 & adj.P.Val < adj.P.Val.Filter )), ]

diffSigOut=rbind(id=colnames(diffSig),diffSig)

write.table(diffSigOut, file=paste0(contrast, ".diff.txt"), sep="\t", quote=F, col.names=F)

#设置热图注释的颜色

bioCol=c("#0066FF","#FF9900","#FF0000","#6E568C","#7CC767","#223D6C","#D20A13","#FFD121","#088247","#11AA4D")

ann_colors=list()

m6aCluCol=bioCol[1:length(levels(factor(allType)))]

names(m6aCluCol)=levels(factor(allType))

ann_colors[["PRGcluster"]]=m6aCluCol[c(comp[1,i], comp[2,i])]

#绘制差异通路热图

termNum=20 #设置显示通路的数目

diffTermName=as.vector(rownames(diffSig))

diffLength=length(diffTermName)

if(diffLength<termNum){termNum=diffLength}

hmGene=diffTermName[1:termNum]

hmExp=data[hmGene,]

pdf(file=paste0(contrast,".heatmap.pdf"), width=10, height=6)

pheatmap(hmExp,

annotation=ann,

annotation_colors = ann_colors,

color = colorRampPalette(c(rep("blue",2), "white", rep("orange",2)))(50),

cluster_cols =F,

show_colnames = F,

gaps_col=as.vector(cumsum(table(Type))),

scale="row",

fontsize = 8,

fontsize_row=6,

fontsize_col=8)

dev.off()

}

22.

#if (!requireNamespace("BiocManager", quietly = TRUE))

# install.packages("BiocManager")

#BiocManager::install("limma")

#BiocManager::install("GSEABase")

#BiocManager::install("GSVA")

#install.packages("ggpubr")

#引用包

library(reshape2)

library(ggpubr)

library(limma)

library(GSEABase)

library(GSVA)

expFile="merge.txt" #表达数据文件

clusterFile="PRGcluster.txt" #分型结果文件

gmtFile="immune.gmt" #免疫基因集文件

setwd("D:\\sx\\140prgTME\\26.ssGSEA") #设置工作目录

#读取表达输入文件,并对输入文件整理

rt=read.table(expFile, header=T, sep="\t", check.names=F)

rt=as.matrix(rt)

rownames(rt)=rt[,1]

exp=rt[,2:ncol(rt)]

dimnames=list(rownames(exp),colnames(exp))

data=matrix(as.numeric(as.matrix(exp)),nrow=nrow(exp),dimnames=dimnames)

data=avereps(data)

#读取基因集文件

geneSets=getGmt(gmtFile, geneIdType=SymbolIdentifier())

#ssGSEA分析

ssgseaScore=gsva(data, geneSets, method='ssgsea', kcdf='Gaussian', abs.ranking=TRUE)

#对ssGSEA打分进行矫正

normalize=function(x){

return((x-min(x))/(max(x)-min(x)))}

ssgseaScore=normalize(ssgseaScore)

#输出ssGSEA打分结果

ssgseaOut=rbind(id=colnames(ssgseaScore), ssgseaScore)

write.table(ssgseaOut,file="ssGSEA.result.txt",sep="\t",quote=F,col.names=F)

#读取分型文件

cluster=read.table(clusterFile, header=T, sep="\t", check.names=F, row.names=1)

#数据合并

ssgseaScore=t(ssgseaScore)

sameSample=intersect(row.names(ssgseaScore), row.names(cluster))

ssgseaScore=ssgseaScore[sameSample,,drop=F]

cluster=cluster[sameSample,,drop=F]

scoreCluster=cbind(ssgseaScore, cluster)

#把数据转换成ggplot2输入文件

data=melt(scoreCluster, id.vars=c("PRGcluster"))

colnames(data)=c("PRGcluster", "Immune", "Fraction")

#绘制箱线图

bioCol=c("#0066FF","#FF9900","#FF0000","#6E568C","#7CC767","#223D6C","#D20A13","#FFD121","#088247","#11AA4D")

bioCol=bioCol[1:length(levels(factor(data[,"PRGcluster"])))]

p=ggboxplot(data, x="Immune", y="Fraction", color="PRGcluster",

ylab="Immune infiltration",

xlab="",

legend.title="PRGcluster",

palette=bioCol)

p=p+rotate_x_text(50)

#保存图形

pdf(file="boxplot.pdf", width=8, height=6.5)

p+stat_compare_means(aes(group=PRGcluster),symnum.args=list(cutpoints = c(0, 0.001, 0.01, 0.05, 1), symbols = c("***", "**", "*", "")),label = "p.signif")

dev.off()

23.

#if (!requireNamespace("BiocManager", quietly = TRUE))

# install.packages("BiocManager")

#BiocManager::install("limma")

#install.packages("ggplot2")

#引用包

library(limma)

library(ggplot2)

expFile="prgGeneExp.txt" #表达数据文件

clusterFile="PRGcluster.txt" #分型结果文件

setwd("D:\\sx\\140prgTME\\27.PCA") #设置工作目录

#读取输入文件,并对输入文件进行整理

rt=read.table(expFile, header=T, sep="\t", check.names=F)

rt=as.matrix(rt)

rownames(rt)=rt[,1]

exp=rt[,2:ncol(rt)]

dimnames=list(rownames(exp),colnames(exp))

data=matrix(as.numeric(as.matrix(exp)),nrow=nrow(exp),dimnames=dimnames)

data=avereps(data)

data=data[rowMeans(data)>0,]

data=t(data)

#PCA分析

data.pca=prcomp(data, scale. = TRUE)

pcaPredict=predict(data.pca)

write.table(pcaPredict, file="newTab.xls", quote=F, sep="\t")

#读取分型文件

cluster=read.table(clusterFile, header=T, sep="\t", check.names=F, row.names=1)

PRGcluster=as.vector(cluster[,1])

#设置颜色

bioCol=c("#0066FF","#FF9900","#FF0000","#6E568C","#7CC767","#223D6C","#D20A13","#FFD121","#088247","#11AA4D")

prgCluCol=bioCol[1:length(levels(factor(PRGcluster)))]

#可视化

PCA=data.frame(PC1=pcaPredict[,1], PC2=pcaPredict[,2], PRGcluster=PRGcluster)

PCA.mean=aggregate(PCA[,1:2], list(PRGcluster=PCA$PRGcluster), mean)

pdf(file="PCA.pdf", width=6.5, height=5)

ggplot(data = PCA, aes(PC1, PC2)) + geom_point(aes(color = PRGcluster)) +

scale_colour_manual(name="PRGcluster", values =prgCluCol)+

theme_bw()+

theme(plot.margin=unit(rep(1.5,4),'lines'))+

annotate("text",x=PCA.mean$PC1, y=PCA.mean$PC2, label=PCA.mean$PRGcluster, cex=7)+

theme(panel.grid.major = element_blank(), panel.grid.minor = element_blank())

dev.off()

24.

#if (!requireNamespace("BiocManager", quietly = TRUE))

# install.packages("BiocManager")

#BiocManager::install("limma")

#install.packages("VennDiagram")

#引用包

library(limma)

library(VennDiagram)

expFile="merge.txt" #表达输入文件

cluFile="PRGcluster.txt" #聚类结果文件

logFCfilter=1 #logFC过滤条件

adj.P.Val.Filter=0.001 #矫正后p值阈值

setwd("D:\\sx\\140prgTME\\28.clusterDiff\\plus2") #设置工作目录

#读取输入文件，并对输入文件整理

rt=read.table(expFile, header=T, sep="\t", check.names=F)

rt=as.matrix(rt)

rownames(rt)=rt[,1]

exp=rt[,2:ncol(rt)]

dimnames=list(rownames(exp),colnames(exp))

data=matrix(as.numeric(as.matrix(exp)),nrow=nrow(exp),dimnames=dimnames)

data=avereps(data)

data=data[rowMeans(data)>0,]

#读取cluster文件

cluster=read.table(cluFile, header=T, sep="\t", check.names=F, row.names=1)

#提取交集文件

sameSample=intersect(colnames(data), row.names(cluster))

data=data[,sameSample]

cluster=cluster[sameSample,]

#差异分析

geneList=list()

Type=as.vector(cluster)

design=model.matrix(~0+factor(Type))

colnames(design)=levels(factor(Type))

comp=combn(levels(factor(Type)), 2)

allDiffGenes=c()

for(i in 1:ncol(comp)){

fit=lmFit(data, design)

contrast=paste0(comp[2,i], "-", comp[1,i])

#print(contrast)

cont.matrix=makeContrasts(contrast, levels=design)

fit2=contrasts.fit(fit, cont.matrix)

fit2=eBayes(fit2)

#输出所有基因的差异情况

allDiff=topTable(fit2,adjust='fdr',number=200000)

allDiffOut=rbind(id=colnames(allDiff),allDiff)

write.table(allDiffOut, file=paste0(contrast, ".all.txt"), sep="\t", quote=F, col.names=F)

#输出显著性的差异结果

diffSig=allDiff[with(allDiff, (abs(logFC)>logFCfilter & adj.P.Val < adj.P.Val.Filter )), ]

diffSigOut=rbind(id=colnames(diffSig),diffSig)

write.table(diffSigOut, file=paste0(contrast, ".diff.txt"), sep="\t", quote=F, col.names=F)

geneList[[contrast]]=row.names(diffSig)

}

#绘制venn图

venn.plot=venn.diagram(geneList,filename=NULL,fill=rainbow(length(geneList)) )

pdf(file="venn.pdf", width=5, height=5)

grid.draw(venn.plot)

dev.off()

#保存交集基因

interGenes=Reduce(intersect,geneList)

write.table(file="interGene.txt",interGenes,sep="\t",quote=F,col.names=F,row.names=F)

#保存交集基因的表达量

interGeneExp=data[interGenes,]

interGeneExp=rbind(id=colnames(interGeneExp), interGeneExp)

write.table(interGeneExp, file="interGeneExp.txt", sep="\t", quote=F, col.names=F)

25.

#install.packages("colorspace")

#install.packages("stringi")

#install.packages("ggplot2")

#install.packages("digest")

#install.packages("GOplot")

#if (!requireNamespace("BiocManager", quietly = TRUE))

# install.packages("BiocManager")

#BiocManager::install("org.Hs.eg.db")

#BiocManager::install("DOSE")

#BiocManager::install("clusterProfiler")

#BiocManager::install("enrichplot")

library(clusterProfiler)

library(org.Hs.eg.db)

library(enrichplot)

library(ggplot2)

library(GOplot)

#过滤

pFilter=0.05

adjPfilter=0.05

setwd("D:\\sx\\140prgTME\\30.KEGG\\KEGGcircle") #设置工作目录

rt=read.table("input.txt",sep="\t",header=T,check.names=F) #读取id.txt文件

genes=as.vector(rt[,1]) #提取基因列表

entrezIDs=mget(genes, org.Hs.egSYMBOL2EG, ifnotfound=NA) #找出基因对应id

entrezIDs=as.character(entrezIDs)

rt=cbind(rt,entrezID=entrezIDs)

rt=rt[is.na(rt[,"entrezID"])==F,] #去除id为NA的基因

gene=rt$entrezID

#KEGG富集分析

kk=enrichKEGG(gene = gene, organism = "hsa", pvalueCutoff =1, qvalueCutoff =1)

KEGG=as.data.frame(kk)

KEGG=KEGG[(KEGG$pvalue<pFilter & KEGG$p.adjust<adjPfilter),]

KEGG$geneID=as.character(sapply(KEGG$geneID,function(x)paste(rt$gene[match(strsplit(x,"/")[[1]],as.character(rt$entrezID))],collapse="/")))

write.table(KEGG,file="KEGG.txt",sep="\t",quote=F,row.names = F) #保存富集结果

#获取KEGG信息

kegg=data.frame(Category = "ALL",ID = KEGG$ID,Term = KEGG$Description, Genes = gsub("/", ", ", KEGG$geneID), adj_pval = KEGG$p.adjust)

#读取基因的logFC

genelist <- data.frame(ID = rt$gene, logFC = rt$logFC)

row.names(genelist)=genelist[,1]

circ <- circle_dat(kegg, genelist)

termNum = 5 #限定Term数

termNum=ifelse(nrow(kegg)<termNum,nrow(kegg),termNum)

geneNum = nrow(genelist) #限定基因数目

#绘制圈图

chord <- chord_dat(circ, genelist[1:geneNum,], kegg$Term[1:termNum])

pdf(file="KEGGcircos.pdf",width = 11,height = 11.2)

GOChord(chord,

space = 0.001, #基因之间的间距

gene.order = 'logFC', #按照logFC对基因排序

gene.space = 0.25, #基因名跟圆圈的相对距离

gene.size = 4, #基因名字体大小

border.size = 0.1, #线条粗细

process.label = 7) #term字体大小

dev.off()

#绘制聚类圈图

pdf(file="KEGGcluster.pdf",width = 12,height = 9)

GOCluster(circ, as.character(kegg[1:termNum,3]))

dev.off()

26．

#if (!requireNamespace("BiocManager", quietly = TRUE))

# install.packages("BiocManager")

#BiocManager::install("limma")

#install.packages('survival')

#引用包

library(limma)

library(survival)

expFile="interGeneExp.txt" #差异基因的表达文件

cliFile="time.txt" #生存数据文件

setwd("D:\\sx\\140prgTME\\31.uniCox\\plus2") #设置工作目录

#读取表达文件，并对输入文件整理

rt=read.table(expFile, header=T, sep="\t", check.names=F)

rt=as.matrix(rt)

rownames(rt)=rt[,1]

exp=rt[,2:ncol(rt)]

dimnames=list(rownames(exp), colnames(exp))

data=matrix(as.numeric(as.matrix(exp)), nrow=nrow(exp), dimnames=dimnames)

data=avereps(data)

data=data[rowMeans(data)>0,]

data1=t(data)

rownames(data1)=gsub("(.*?)\\_(.*?)", "\\2", rownames(data1))

#读取生存数据

cli=read.table(cliFile, header=T, sep="\t", check.names=F, row.names=1) #读取临床文件

cli$futime=cli$futime/365

#数据合并

sameSample=intersect(row.names(data1), row.names(cli))

data1=data1[sameSample,,drop=F]

cli=cli[sameSample,,drop=F]

rt=cbind(cli, data1)

#对基因进行循环，找出预后相关的基因

outTab=data.frame()

sigGenes=c()

for(i in colnames(rt[,3:ncol(rt)])){

#cox分析

cox <- coxph(Surv(futime, fustat) ~ rt[,i], data = rt)

coxSummary = summary(cox)

coxP=coxSummary$coefficients[,"Pr(>|z|)"]

if(coxP<0.05){

sigGenes=c(sigGenes,i)

outTab=rbind(outTab,

cbind(id=i,

HR=coxSummary$conf.int[,"exp(coef)"],

HR.95L=coxSummary$conf.int[,"lower .95"],

HR.95H=coxSummary$conf.int[,"upper .95"],

pvalue=coxSummary$coefficients[,"Pr(>|z|)"])

)

}

}

#输出单因素的结果

write.table(outTab,file="uniCox.txt",sep="\t",row.names=F,quote=F)

#保存单因素显著基因的表达量

sigGeneExp=data[sigGenes,]

sigGeneExp=rbind(id=colnames(sigGeneExp), sigGeneExp)

write.table(sigGeneExp, file="uniSigGeneExp.txt", sep="\t", quote=F, col.names=F)

#保存单因素显著基因表达和生存合并的文件

sigExpTime=rt[,c("futime", "fustat", sigGenes)]

sigExpTime=rbind(id=colnames(sigExpTime), sigExpTime)

write.table(sigExpTime, file="uniSigExpTime.txt", sep="\t", quote=F, col.names=F)

27.

#if (!requireNamespace("BiocManager", quietly = TRUE))

# install.packages("BiocManager")

#BiocManager::install("limma")

#if (!requireNamespace("BiocManager", quietly = TRUE))

# install.packages("BiocManager")

#BiocManager::install("ConsensusClusterPlus")

#引用包

library(limma)

library(ConsensusClusterPlus)

expFile="uniSigGeneExp.txt" #表达输入文件

workDir="D:\\sx\\140prgTME\\32.geneCluster" #设置工作目录

setwd(workDir) #设置工作目录

#读取输入文件，并对输入文件整理

rt=read.table(expFile, header=T, sep="\t", check.names=F)

rt=as.matrix(rt)

rownames(rt)=rt[,1]

exp=rt[,2:ncol(rt)]

dimnames=list(rownames(exp), colnames(exp))

data=matrix(as.numeric(as.matrix(exp)), nrow=nrow(exp), dimnames=dimnames)

data=avereps(data)

data=data[rowMeans(data)>0,]

#聚类

maxK=9 #设置最大的k值

results=ConsensusClusterPlus(data,

maxK=maxK,

reps=50,

pItem=0.8,

pFeature=1,

title=workDir,

clusterAlg="km",

distance="euclidean",

seed=123456,

plot="png")

#输出分型结果

clusterNum=3 #分几类，根据判断标准判断

cluster=results[[clusterNum]][["consensusClass"]]

cluster=as.data.frame(cluster)

colnames(cluster)=c("geneCluster")

letter=c("A","B","C","D","E","F","G")

uniqClu=levels(factor(cluster$geneCluster))

cluster$geneCluster=letter[match(cluster$geneCluster, uniqClu)]

clusterOut=rbind(ID=colnames(cluster), cluster)

write.table(clusterOut, file="geneCluster.txt", sep="\t", quote=F, col.names=F)

28.

#install.packages("survival")

#install.packages("survminer")

#引用包

library(survival)

library(survminer)

clusterFile="geneCluster.txt" #基因分型的结果文件

cliFile="time.txt" #生存数据文件

setwd("D:\\sx\\140prgTME\\33.geneClusterSur") #设置工作目录

#读取输入文件

cluster=read.table(clusterFile, header=T, sep="\t", check.names=F, row.names=1)

rownames(cluster)=gsub("(.*?)\\_(.*?)", "\\2", rownames(cluster))

cli=read.table(cliFile, header=T, sep="\t", check.names=F, row.names=1)

colnames(cli)=c("futime", "fustat")

cli$futime=cli$futime/365

#数据合并

sameSample=intersect(row.names(cluster), row.names(cli))

rt=cbind(cli[sameSample,,drop=F], cluster[sameSample,,drop=F])

#生存差异统计

length=length(levels(factor(rt$geneCluster)))

diff=survdiff(Surv(futime, fustat) ~ geneCluster, data = rt)

pValue=1-pchisq(diff$chisq, df=length-1)

if(pValue<0.001){

pValue="p<0.001"

}else{

pValue=paste0("p=",sprintf("%.03f",pValue))

}

fit <- survfit(Surv(futime, fustat) ~ geneCluster, data = rt)

#print(surv_median(fit))

#绘制生存曲线

bioCol=c("#0066FF","#FF9900","#FF0000","#6E568C","#7CC767","#223D6C","#D20A13","#FFD121","#088247","#11AA4D")

bioCol=bioCol[1:length(levels(factor(rt[,"geneCluster"])))]

surPlot=ggsurvplot(fit,

data=rt,

conf.int=F,

pval=pValue,

pval.size=6,

legend.title="geneCluster",

legend.labs=levels(factor(rt[,"geneCluster"])),

legend = c(0.8, 0.8),

font.legend=10,

xlab="Time(years)",

break.time.by = 1,

palette = bioCol,

surv.median.line = "hv",

risk.table=T,

cumevents=F,

risk.table.height=.25)

pdf(file="survival.pdf", onefile = FALSE, width=7, height=5.5)

print(surPlot)

dev.off()

29.

#install.packages("pheatmap")

library(pheatmap) #引用包

expFile="uniSigGeneExp.txt" #表达数据文件

geneCluFile="geneCluster.txt" #基因分型的结果文件

prgCluFile="PRGcluster.txt" #细胞焦亡分型的结果文件

cliFile="clinical.txt" #临床数据文件

setwd("D:\\sx\\140prgTME\\34.geneHeatmap") #设置工作目录

#读取输入文件

exp=read.table(expFile, header=T, sep="\t", check.names=F, row.names=1)

prgClu=read.table(prgCluFile, header=T, sep="\t", check.names=F, row.names=1)

geneClu=read.table(geneCluFile, header=T, sep="\t", check.names=F, row.names=1)

#合并数据

exp=as.data.frame(t(exp))

sameSample=intersect(row.names(exp), row.names(prgClu))

exp=exp[sameSample,,drop=F]

expData=cbind(exp, geneCluster=geneClu[sameSample,], PRGcluster=prgClu[sameSample,])

Project=gsub("(.*?)\\_.*", "\\1", rownames(expData))

rownames(expData)=gsub("(.*?)\\_(.*?)", "\\2", rownames(expData))

expData=cbind(expData, Project)

#合并临床数据

cli=read.table(cliFile, header=T, sep="\t", check.names=F, row.names=1)

cli[,"Age"]=ifelse(cli[,"Age"]=="unknow", "unknow", ifelse(cli[,"Age"]>65,">65","<=65"))

sameSample=intersect(row.names(expData), row.names(cli))

expData=expData[sameSample,,drop=F]

cli=cli[sameSample,,drop=F]

data=cbind(expData, cli)

#提取热图数据

data=data[order(data$geneCluster),]

Type=data[,((ncol(data)-2-ncol(cli)):ncol(data))]

data=t(data[,1:(ncol(expData)-3)])

#聚类颜色

bioCol=c("#0066FF","#FF9900","#FF0000","#6E568C","#7CC767","#223D6C","#D20A13","#FFD121","#088247","#11AA4D")

ann_colors=list()

PRGcol=bioCol[1:length(levels(factor(Type$PRGcluster)))]

names(PRGcol)=levels(factor(Type$PRGcluster))

ann_colors[["PRGcluster"]]=PRGcol

GENEcol=bioCol[1:length(levels(factor(Type$geneCluster)))]

names(GENEcol)=levels(factor(Type$geneCluster))

ann_colors[["geneCluster"]]=GENEcol

#热图可视化

pdf("heatmap.pdf", height=6, width=8)

pheatmap(data,

annotation=Type,

annotation_colors = ann_colors,

color = colorRampPalette(c(rep("blue",5), "white", rep("orange",5)))(50),

cluster_cols =F,

cluster_rows =F,

scale="row",

show_colnames=F,

show_rownames=F,

fontsize=6,

fontsize_row=2,

fontsize_col=6)

dev.off()

30.

#if (!requireNamespace("BiocManager", quietly = TRUE))

# install.packages("BiocManager")

#BiocManager::install("limma")

#install.packages("reshape2")

#install.packages("ggpubr")

#引用包

library(limma)

library(reshape2)

library(ggpubr)

expFile="prgGeneExp.txt" #表达数据文件

geneCluFile="geneCluster.txt" #基因分型的结果文件

setwd("D:\\sx\\140prgTME\\35.prgClusterDiff") #设置工作目录

#读取表达输入文件

rt=read.table(expFile, header=T, sep="\t", check.names=F)

rt=as.matrix(rt)

rownames(rt)=rt[,1]

exp=rt[,2:ncol(rt)]

dimnames=list(rownames(exp),colnames(exp))

data=matrix(as.numeric(as.matrix(exp)),nrow=nrow(exp),dimnames=dimnames)

data=avereps(data)

data=t(data)

#读取基因分型文件

geneClu=read.table(geneCluFile, header=T, sep="\t", check.names=F, row.names=1)

#合并数据

sameSample=intersect(row.names(data), row.names(geneClu))

expClu=cbind(data[sameSample,,drop=F], geneClu[sameSample,,drop=F])

#提取差异显著的基因

sigGene=c()

for(i in colnames(expClu)[1:(ncol(expClu)-1)]){

if(sd(expClu[,i])<0.001){next}

if(length(levels(factor(expClu[,"geneCluster"])))>2){

test=kruskal.test(expClu[,i] ~ expClu[,"geneCluster"])

}else{

test=wilcox.test(expClu[,i] ~ expClu[,"geneCluster"])

}

pvalue=test$p.value

if(pvalue<0.05){

sigGene=c(sigGene, i)

}

}

sigGene=c(sigGene, "geneCluster")

expClu=expClu[,sigGene]

#把数据转换成ggplot2输入文件

data=melt(expClu, id.vars=c("geneCluster"))

colnames(data)=c("geneCluster", "Gene", "Expression")

#设置颜色

bioCol=c("#0066FF","#FF9900","#FF0000","#6E568C","#7CC767","#223D6C","#D20A13","#FFD121","#088247","#11AA4D")

bioCol=bioCol[1:length(levels(factor(data[,"geneCluster"])))]

#绘制箱线图

p=ggboxplot(data, x="Gene", y="Expression", color = "geneCluster",

xlab="",

ylab="Gene expression",

legend.title="geneCluster",

palette = bioCol,

width=1)

p=p+rotate_x_text(60)

p1=p+stat_compare_means(aes(group=geneCluster),

symnum.args=list(cutpoints = c(0, 0.001, 0.01, 0.05, 1), symbols = c("***", "**", "*", " ")),

label = "p.signif")

#输出箱线图

pdf(file="boxplot.pdf", width=9.5, height=6)

print(p1)

dev.off()

31.

#install.packages("glmnet")

#install.packages("survival")

#install.packages('survminer')

#引用包

library(glmnet)

library(survival)

library(survminer)

inputFile="uniSigExp.txt" #单因素显著基因的表达输入文件

setwd("D:\\sx\\127FerrLnc\\15.model") #设置工作目录

rt=read.table(inputFile, header=T, sep="\t", check.names=F, row.names=1) #读取输入文件

#COX模型构建

multiCox=coxph(Surv(futime, fustat) ~ ., data = rt)

multiCox=step(multiCox, direction="both")

multiCoxSum=summary(multiCox)

#输出模型相关信息

outMultiTab=data.frame()

outMultiTab=cbind(

coef=multiCoxSum$coefficients[,"coef"],

HR=multiCoxSum$conf.int[,"exp(coef)"],

HR.95L=multiCoxSum$conf.int[,"lower .95"],

HR.95H=multiCoxSum$conf.int[,"upper .95"],

pvalue=multiCoxSum$coefficients[,"Pr(>|z|)"])

outMultiTab=cbind(id=row.names(outMultiTab),outMultiTab)

write.table(outMultiTab, file="multiCox.txt", sep="\t", row.names=F, quote=F)

#输出风险文件

score=predict(multiCox, type="risk", newdata=rt)

coxGene=rownames(multiCoxSum$coefficients)

coxGene=gsub("`", "", coxGene)

outCol=c("futime", "fustat", coxGene)

risk=as.vector(ifelse(score>median(score), "high", "low"))

outTab=cbind(rt[,outCol], riskScore=as.vector(score), risk)

write.table(cbind(id=rownames(outTab),outTab), file="risk.txt", sep="\t", quote=F, row.names=F)

32.

#install.packages("survival")

#install.packages("survminer")

#引用包

library(survival)

library(survminer)

setwd("D:\\sx\\127FerrLnc\\16.survival") #设置工作目录

#定义生存曲线的函数

bioSurvival=function(inputFile=null, outFile=null){

#读取输入文件

rt=read.table(inputFile, header=T, sep="\t", check.names=F)

#比较高低风险组生存差异，得到显著性p值

diff=survdiff(Surv(futime, fustat) ~ risk, data=rt)

pValue=1-pchisq(diff$chisq, df=1)

if(pValue<0.001){

pValue="p<0.001"

}else{

pValue=paste0("p=",sprintf("%.03f",pValue))

}

fit <- survfit(Surv(futime, fustat) ~ risk, data = rt)

#print(surv_median(fit))

#绘制生存曲线

surPlot=ggsurvplot(fit,

data=rt,

conf.int=T,

pval=pValue,

pval.size=6,

surv.median.line = "hv",

legend.title="Risk",

legend.labs=c("High risk", "Low risk"),

xlab="Time(years)",

break.time.by = 1,

palette=c("orange", "blue"),

risk.table=TRUE,

risk.table.title="",

risk.table.col = "strata",

risk.table.height=.25)

pdf(file=outFile, onefile=FALSE, width=7.5, height=5.5)

print(surPlot)

dev.off()

}

#调用函数，绘制生存曲线

bioSurvival(inputFile="risk.txt", outFile="survival.pdf")

33.

#install.packages("pheatmap")

library(pheatmap) #引用包

setwd("D:\\sx\\127FerrLnc\\17.riskPlot") #设置工作目录

#定义风险曲线的函数

bioRiskPlot=function(inputFile=null, riskScoreFile=null, survStatFile=null, heatmapFile=null){

rt=read.table(inputFile, header=T, sep="\t", check.names=F, row.names=1) #读取输入文件

rt=rt[order(rt$riskScore),] #按照风险打分对样品排序

#绘制风险曲线

riskClass=rt[,"risk"]

lowLength=length(riskClass[riskClass=="low"])

highLength=length(riskClass[riskClass=="high"])

lowMax=max(rt$riskScore[riskClass=="low"])

line=rt[,"riskScore"]

line[line>10]=10

pdf(file=riskScoreFile, width=7, height=4)

plot(line, type="p", pch=20,

xlab="Patients (increasing risk socre)", ylab="Risk score",

col=c(rep("blue",lowLength),rep("orange",highLength)) )

abline(h=lowMax,v=lowLength,lty=2)

legend("topleft", c("High risk", "Low Risk"),bty="n",pch=19,col=c("orange","blue"),cex=1.2)

dev.off()

#绘制生存状态图

color=as.vector(rt$fustat)

color[color==1]="orange"

color[color==0]="blue"

pdf(file=survStatFile, width=7, height=4)

plot(rt$futime, pch=19,

xlab="Patients (increasing risk socre)", ylab="Survival time (years)",

col=color)

legend("topleft", c("Dead", "Alive"),bty="n",pch=19,col=c("orange","blue"),cex=1.2)

abline(v=lowLength,lty=2)

dev.off()

#绘制风险热图

rt1=rt[c(3:(ncol(rt)-2))]

rt1=t(rt1)

annotation=data.frame(type=rt[,ncol(rt)])

rownames(annotation)=rownames(rt)

pdf(file=heatmapFile, width=7, height=4)

pheatmap(rt1,

annotation=annotation,

cluster_cols = F,

cluster_rows = F,

show_colnames = F,

scale="row",

color = colorRampPalette(c(rep("blue",5), "white", rep("orange",5)))(50),

fontsize_col=3,

fontsize=7,

fontsize_row=8)

dev.off()

}

#调用函数，绘制风险曲线

bioRiskPlot(inputFile="risk.txt",

riskScoreFile="riskScore.pdf",

survStatFile="survStat.pdf",

heatmapFile="heatmap.pdf")

34.

#install.packages('survival')

library(survival) #引用包

setwd("D:\\sx\\127FerrLnc\\18.indep") #设置工作目录

############绘制森林图函数############

bioForest=function(coxFile=null, forestFile=null, forestCol=null){

#读取输入文件

rt <- read.table(coxFile, header=T, sep="\t", check.names=F, row.names=1)

gene <- rownames(rt)

hr <- sprintf("%.3f",rt$"HR")

hrLow <- sprintf("%.3f",rt$"HR.95L")

hrHigh <- sprintf("%.3f",rt$"HR.95H")

Hazard.ratio <- paste0(hr,"(",hrLow,"-",hrHigh,")")

pVal <- ifelse(rt$pvalue<0.001, "<0.001", sprintf("%.3f", rt$pvalue))

#输出图形

pdf(file=forestFile, width=6.5, height=4.5)

n <- nrow(rt)

nRow <- n+1

ylim <- c(1,nRow)

layout(matrix(c(1,2),nc=2),width=c(3,2.5))

#绘制森林图左边的临床信息

xlim = c(0,3)

par(mar=c(4,2.5,2,1))

plot(1,xlim=xlim,ylim=ylim,type="n",axes=F,xlab="",ylab="")

text.cex=0.8

text(0,n:1,gene,adj=0,cex=text.cex)

text(1.5-0.5*0.2,n:1,pVal,adj=1,cex=text.cex);text(1.5-0.5*0.2,n+1,'pvalue',cex=text.cex,font=2,adj=1)

text(3.1,n:1,Hazard.ratio,adj=1,cex=text.cex);text(3.1,n+1,'Hazard ratio',cex=text.cex,font=2,adj=1)

#绘制右边的森林图

par(mar=c(4,1,2,1),mgp=c(2,0.5,0))

xlim = c(0,max(as.numeric(hrLow),as.numeric(hrHigh)))

plot(1,xlim=xlim,ylim=ylim,type="n",axes=F,ylab="",xaxs="i",xlab="Hazard ratio")

arrows(as.numeric(hrLow),n:1,as.numeric(hrHigh),n:1,angle=90,code=3,length=0.05,col="darkblue",lwd=3)

abline(v=1, col="black", lty=2, lwd=2)

boxcolor = ifelse(as.numeric(hr) > 1, forestCol, forestCol)

points(as.numeric(hr), n:1, pch = 15, col = boxcolor, cex=2)

axis(1)

dev.off()

}

############绘制森林图函数############

#定义独立预后分析函数

indep=function(riskFile=null,cliFile=null,uniOutFile=null,multiOutFile=null,uniForest=null,multiForest=null){

risk=read.table(riskFile, header=T, sep="\t", check.names=F, row.names=1) #读取风险文件

cli=read.table(cliFile, header=T, sep="\t", check.names=F, row.names=1) #读取临床文件

#数据合并

sameSample=intersect(row.names(cli),row.names(risk))

risk=risk[sameSample,]

cli=cli[sameSample,]

rt=cbind(futime=risk[,1], fustat=risk[,2], cli, riskScore=risk[,(ncol(risk)-1)])

#单因素独立预后分析

uniTab=data.frame()

for(i in colnames(rt[,3:ncol(rt)])){

cox <- coxph(Surv(futime, fustat) ~ rt[,i], data = rt)

coxSummary = summary(cox)

uniTab=rbind(uniTab,

cbind(id=i,

HR=coxSummary$conf.int[,"exp(coef)"],

HR.95L=coxSummary$conf.int[,"lower .95"],

HR.95H=coxSummary$conf.int[,"upper .95"],

pvalue=coxSummary$coefficients[,"Pr(>|z|)"])

)

}

write.table(uniTab,file=uniOutFile,sep="\t",row.names=F,quote=F)

bioForest(coxFile=uniOutFile, forestFile=uniForest, forestCol="green")

#多因素独立预后分析

uniTab=uniTab[as.numeric(uniTab[,"pvalue"])<1,]

rt1=rt[,c("futime", "fustat", as.vector(uniTab[,"id"]))]

multiCox=coxph(Surv(futime, fustat) ~ ., data = rt1)

multiCoxSum=summary(multiCox)

multiTab=data.frame()

multiTab=cbind(

HR=multiCoxSum$conf.int[,"exp(coef)"],

HR.95L=multiCoxSum$conf.int[,"lower .95"],

HR.95H=multiCoxSum$conf.int[,"upper .95"],

pvalue=multiCoxSum$coefficients[,"Pr(>|z|)"])

multiTab=cbind(id=row.names(multiTab),multiTab)

write.table(multiTab,file=multiOutFile,sep="\t",row.names=F,quote=F)

bioForest(coxFile=multiOutFile, forestFile=multiForest, forestCol="red")

}

#调用函数，进行独立预后分析

indep(riskFile="risk.txt",

cliFile="clinical.txt",

uniOutFile="uniCox.txt",

multiOutFile="multiCox.txt",

uniForest="uniForest.pdf",

multiForest="multiForest.pdf")

35.

#install.packages("survival")

#install.packages("survminer")

#install.packages("timeROC")

#引用包

library(survival)

library(survminer)

library(timeROC)

riskFile="risk.txt" #风险输入文件

cliFile="clinical.txt" #临床数据文件

setwd("D:\\sx\\127FerrLnc\\19.ROC") #修改工作目录

#读取风险输入文件

risk=read.table(riskFile, header=T, sep="\t", check.names=F, row.names=1)

risk=risk[,c("futime", "fustat", "riskScore")]

#读取临床数据文件

cli=read.table(cliFile, header=T, sep="\t", check.names=F, row.names=1)

#合并数据

samSample=intersect(row.names(risk), row.names(cli))

risk1=risk[samSample,,drop=F]

cli=cli[samSample,,drop=F]

rt=cbind(risk1, cli)

#定义颜色

bioCol=rainbow(ncol(rt)-1, s=0.9, v=0.9)

######绘制1 2 3年的ROC曲线######

ROC_rt=timeROC(T=risk$futime,delta=risk$fustat,

marker=risk$riskScore,cause=1,

weighting='aalen',

times=c(1,2,3),ROC=TRUE)

pdf(file="ROC.pdf", width=5, height=5)

plot(ROC_rt,time=1,col=bioCol[1],title=FALSE,lwd=2)

plot(ROC_rt,time=2,col=bioCol[2],add=TRUE,title=FALSE,lwd=2)

plot(ROC_rt,time=3,col=bioCol[3],add=TRUE,title=FALSE,lwd=2)

legend('bottomright',

c(paste0('AUC at 1 years: ',sprintf("%.03f",ROC_rt$AUC[1])),

paste0('AUC at 2 years: ',sprintf("%.03f",ROC_rt$AUC[2])),

paste0('AUC at 3 years: ',sprintf("%.03f",ROC_rt$AUC[3]))),

col=bioCol[1:3], lwd=2, bty = 'n')

dev.off()

######绘制临床的ROC曲线######

predictTime=1 #定义预测年限

aucText=c()

pdf(file="cliROC.pdf", width=6, height=6)

#绘制风险得分的ROC曲线

i=3

ROC_rt=timeROC(T=risk$futime,

delta=risk$fustat,

marker=risk$riskScore, cause=1,

weighting='aalen',

times=c(predictTime),ROC=TRUE)

plot(ROC_rt, time=predictTime, col=bioCol[i-2], title=FALSE, lwd=2)

aucText=c(paste0("Risk", ", AUC=", sprintf("%.3f",ROC_rt$AUC[2])))

abline(0,1)

#对临床数据进行循环，绘制临床数据的ROC曲线

for(i in 4:ncol(rt)){

ROC_rt=timeROC(T=rt$futime,

delta=rt$fustat,

marker=rt[,i], cause=1,

weighting='aalen',

times=c(predictTime),ROC=TRUE)

plot(ROC_rt, time=predictTime, col=bioCol[i-2], title=FALSE, lwd=2, add=TRUE)

aucText=c(aucText, paste0(colnames(rt)[i],", AUC=",sprintf("%.3f",ROC_rt$AUC[2])))

}

#绘制图例，得到ROC曲线下的面积

legend("bottomright", aucText,lwd=2,bty="n",col=bioCol[1:(ncol(rt)-1)])

dev.off()

36.

#if (!requireNamespace("BiocManager", quietly = TRUE))

# install.packages("BiocManager")

#BiocManager::install("limma")

#install.packages("pheatmap")

#引用包

library(limma)

library(pheatmap)

cliFile="clinical.txt" #临床数据文件

riskFile="risk.txt" #风险文件

setwd("D:\\sx\\127FerrLnc\\24.cliHeatmap")

#读取临床数据文件

cli=read.table(cliFile, header=T, sep="\t", check.names=F, row.names=1)

cli[,"Age"]=ifelse(cli[,"Age"]=="unknow", "unknow", ifelse(cli[,"Age"]>65, ">65","<=65") )

#读取风险文件

risk=read.table(riskFile, header=T, sep="\t", check.names=F, row.names=1)

#合并数据

samSample=intersect(row.names(risk), row.names(cli))

cli=cli[samSample,,drop=F]

risk=risk[samSample,,drop=F]

data=cbind(risk, cli)

data=data[order(data$riskScore),,drop=F] #根据风险打分对样品排序

Type=data[,(ncol(risk):ncol(data))] #提取临床信息，作为热图注释文件

exp=data[,(3:(ncol(risk)-2))] #提取lncRNA表达量

#对临床性状进行循环，得到显著性标记

sigVec=c("risk")

for(clinical in colnames(Type[,2:ncol(Type)])){

data=Type[c("risk", clinical)]

colnames(data)=c("risk", "clinical")

data=data[(data[,"clinical"]!="unknow"),]

tableStat=table(data)

stat=chisq.test(tableStat)

pvalue=stat$p.value

Sig=ifelse(pvalue<0.001,"***",ifelse(pvalue<0.01,"**",ifelse(pvalue<0.05,"*","")))

sigVec=c(sigVec, paste0(clinical, Sig))

#print(paste(clinical, pvalue, Sig, sep="\t"))

}

colnames(Type)=sigVec

#定义热图注释的颜色

colorList=list()

#Type=Type[apply(Type,1,function(x)any(is.na(match('unknow',x)))),,drop=F]

bioCol=c("#FFCCFF","#0066FF","#ED1299", "#0DBC21", "#246B93", "#CC8E12", "#D561DD",

"#6AD157", "#F7AA5D", "#9ED84E", "#39BA30", "#373BBF", "#A1CE4C", "#EF3BB6", "#D66551",

"#1a918f", "#ddd53e", "#ff66fc", "#2927c4", "#57e559" ,"#8e3af4" ,"#f9a270" ,"#22547f", "#db5e92",

"#4aef7b", "#e86502", "#99db27", "#e07233", "#8249aa","#cebb10", "#03827f", "#931635", "#ff523f",

"#edd05e", "#6f25e8", "#0dbc21", "#167275", "#280f7a", "#6373ed", "#5b910f" ,"#7b34c1" ,"#0cf29a" ,"#d80fc1",

"#dd27ce", "#07a301", "#391c82", "#2baeb5","#925bea", "#09f9f5", "#63ff4f")

j=0

for(cli in colnames(Type[,1:ncol(Type)])){

cliLength=length(levels(factor(Type[,cli])))

cliCol=bioCol[(j+1):(j+cliLength)]

j=j+cliLength

names(cliCol)=levels(factor(Type[,cli]))

if("unknow" %in% levels(factor(Type[,cli]))){

cliCol["unknow"]="grey75"}

colorList[[cli]]=cliCol

}

#热图可视化

pdf("heatmap.pdf", width=9, height=6)

pheatmap(t(exp),

annotation=Type,

annotation_colors = colorList,

color = colorRampPalette(c(rep("blue",5), "white", rep("orange",5)))(100),

cluster_cols =F,

cluster_rows =F,

scale="row",

show_colnames=F,

show_rownames=T,

fontsize=6,

fontsize_row=7,

fontsize_col=6)

dev.off()

37.

#install.packages("ggplot2")

#install.packages("ggalluvial")

#引用包

library(ggalluvial)

library(ggplot2)

library(dplyr)

prgCluFile="PRGcluster.txt" #细胞焦亡分型的结果文件

geneCluFile="geneCluster.txt" #基因分型的结果文件

riskFile="risk.all.txt" #风险文件

setwd("C:\\biowolf\\prgTME\\37.ggalluvial") #设置工作目录

#读取输入文件

prgClu=read.table(prgCluFile, header=T, sep="\t", check.names=F, row.names=1)

geneClu=read.table(geneCluFile, header=T, sep="\t", check.names=F, row.names=1)

risk=read.table(riskFile, header=T, sep="\t", check.names=F, row.names=1)

#合并数据

twoCluster=cbind(prgClu, geneClu)

rownames(twoCluster)=gsub("(.*?)\\_(.*?)", "\\2", rownames(twoCluster))

sameSample=intersect(row.names(twoCluster), row.names(risk))

rt=cbind(risk[sameSample,,drop=F], twoCluster[sameSample,,drop=F])

#准备桑基图输入文件

rt=rt[,c("PRGcluster", "geneCluster", "risk", "fustat")]

colnames(rt)=c("PRGcluster", "geneCluster", "Risk", "Fustat")

rt[,"Fustat"]=ifelse(rt[,"Fustat"]==0, "Alive", "Dead")

corLodes=to_lodes_form(rt, axes = 1:ncol(rt), id = "Cohort")

#得到输出文件

pdf(file="ggalluvial.pdf", width=6, height=5.5)

mycol=rep(c("#0066FF","#FF9900","#FF0000","#029149","#6E568C","#E0367A","#D8D155","#223D6C","#D20A13","#431A3D","#91612D","#FFD121","#088247","#11AA4D","#58CDD9","#7A142C","#5D90BA","#64495D","#7CC767"),15)

ggplot(corLodes, aes(x = x, stratum = stratum, alluvium = Cohort,fill = stratum, label = stratum)) +

scale_x_discrete(expand = c(0, 0)) +

#用aes.flow控制线条颜色，forward说明颜色和前面的柱状图一致，backward说明和后面的柱状图一致。

geom_flow(width = 2/10,aes.flow = "forward") +

geom_stratum(alpha = .9,width = 2/10) +

scale_fill_manual(values = mycol) +

#size=3代表字体大小

geom_text(stat = "stratum", size = 3,color="black") +

xlab("") + ylab("") + theme_bw() +

theme(axis.line = element_blank(),axis.ticks = element_blank(),axis.text.y = element_blank()) + #去掉坐标轴

theme(panel.grid =element_blank()) +

theme(panel.border = element_blank()) +

ggtitle("") + guides(fill = FALSE)

dev.off()

38.

#if (!requireNamespace("BiocManager", quietly = TRUE))

# install.packages("BiocManager")

#BiocManager::install("limma")

#install.packages("ggpubr")

#引用包

library(limma)

library(ggpubr)

prgCluFile="PRGcluster.txt" #细胞焦亡分型文件

geneCluFile="geneCluster.txt" #基因分型文件

scoreFile="risk.all.txt" #风险文件

setwd("C:\\biowolf\\prgTME\\38.clusterRisk") #设置工作目录

#读取输入文件

prgClu=read.table(prgCluFile, header=T, sep="\t", check.names=F, row.names=1)

geneClu=read.table(geneCluFile, header=T, sep="\t", check.names=F, row.names=1)

score=read.table(scoreFile, header=T, sep="\t", check.names=F, row.names=1)

#合并数据

twoCluster=cbind(prgClu, geneClu)

rownames(twoCluster)=gsub("(.*?)\\_(.*?)", "\\2", rownames(twoCluster))

sameSample=intersect(row.names(twoCluster), row.names(score))

data=cbind(score[sameSample,,drop=F], twoCluster[sameSample,,drop=F])

#######细胞焦亡分型的箱线图########

#设置比较组

data$PRGcluster=factor(data$PRGcluster, levels=levels(factor(data$PRGcluster)))

group=levels(factor(data$PRGcluster))

comp=combn(group, 2)

my_comparisons=list()

for(i in 1:ncol(comp)){my_comparisons[[i]]<-comp[,i]}

#定义颜色

bioCol=c("#0066FF","#FF9900","#FF0000","#6E568C","#7CC767","#223D6C","#D20A13","#FFD121","#088247","#11AA4D")

bioCol=bioCol[1:length(levels(factor(data$PRGcluster)))]

#绘制boxplot

boxplot=ggboxplot(data, x="PRGcluster", y="riskScore", color="PRGcluster",

xlab="PRGcluster",

ylab="Risk score",

legend.title="PRGcluster",

palette=bioCol,

add = "jitter")+

stat_compare_means(comparisons = my_comparisons)

#输出图片

pdf(file="PRGcluster.pdf", width=5, height=4.5)

print(boxplot)

dev.off()

#######细胞焦亡分型的箱线图########

#######基因分型的箱线图########

#设置比较组

data$geneCluster=factor(data$geneCluster, levels=levels(factor(data$geneCluster)))

group=levels(factor(data$geneCluster))

comp=combn(group, 2)

my_comparisons=list()

for(i in 1:ncol(comp)){my_comparisons[[i]]<-comp[,i]}

#定义颜色

bioCol=c("#0066FF","#FF9900","#FF0000","#6E568C","#7CC767","#223D6C","#D20A13","#FFD121","#088247","#11AA4D")

bioCol=bioCol[1:length(levels(factor(data$geneCluster)))]

#绘制boxplot

boxplot=ggboxplot(data, x="geneCluster", y="riskScore", color="geneCluster",

xlab="geneCluster",

ylab="Risk score",

legend.title="geneCluster",

palette=bioCol,

add = "jitter")+

stat_compare_means(comparisons = my_comparisons)

#输出图片

pdf(file="geneCluster.pdf", width=5, height=4.5)

print(boxplot)

dev.off()

#######基因分型的箱线图########

39.

#if (!requireNamespace("BiocManager", quietly = TRUE))

# install.packages("BiocManager")

#BiocManager::install("limma")

#install.packages("ggplot2")

#install.packages("ggpubr")

#引用包

library(limma)

library(reshape2)

library(ggplot2)

library(ggpubr)

expFile="merge.txt" #表达数据文件

geneFile="gene.txt" #基因列表文件

riskFile="risk.all.txt" #风险文件

setwd("C:\\biowolf\\prgTME\\39.geneDiff") #设置工作目录

#读取基因表达文件,并对数据进行处理

rt=read.table(expFile, header=T, sep="\t", check.names=F)

rt=as.matrix(rt)

rownames(rt)=rt[,1]

exp=rt[,2:ncol(rt)]

dimnames=list(rownames(exp),colnames(exp))

data=matrix(as.numeric(as.matrix(exp)),nrow=nrow(exp),dimnames=dimnames)

data=avereps(data)

#读取基因列表文件，并且提取基因的表达量

gene=read.table(geneFile, header=T, sep="\t", check.names=F)

sameGene=intersect(row.names(data),as.vector(gene[,1]))

data=t(data[sameGene,])

rownames(data)=gsub("(.*?)\\_(.*?)", "\\2", rownames(data))

#合并数据

risk=read.table(riskFile, sep="\t", header=T, check.names=F, row.names=1)

sameSample=intersect(row.names(data),row.names(risk))

rt1=cbind(data[sameSample,],risk[sameSample,])

rt1=rt1[,c(sameGene,"risk")]

#提取显著差异的基因

sigGene=c()

for(i in colnames(rt1)[1:(ncol(rt1)-1)]){

if(sd(rt1[,i])<0.001){next}

wilcoxTest=wilcox.test(rt1[,i] ~ rt1[,"risk"])

pvalue=wilcoxTest$p.value

if(wilcoxTest$p.value<0.05){

sigGene=c(sigGene, i)

}

}

sigGene=c(sigGene, "risk")

rt1=rt1[,sigGene]

#把数据转换成ggplot2输入文件

rt1=melt(rt1,id.vars=c("risk"))

colnames(rt1)=c("risk","Gene","Expression")

#设置比较组

group=levels(factor(rt1$risk))

rt1$risk=factor(rt1$risk, levels=c("low","high"))

comp=combn(group,2)

my_comparisons=list()

for(j in 1:ncol(comp)){my_comparisons[[j]]<-comp[,j]}

#绘制箱线图

boxplot=ggboxplot(rt1, x="Gene", y="Expression", fill="risk",

xlab="",

ylab="Gene expression",

legend.title="Risk",

width=0.8,

palette = c("#0066FF", "#FF0000") )+

rotate_x_text(50)+

stat_compare_means(aes(group=risk),

method="wilcox.test",

symnum.args=list(cutpoints=c(0, 0.001, 0.01, 0.05, 1), symbols=c("***", "**", "*", "ns")), label="p.signif")

#输出图片

pdf(file="genediff.pdf", width=8, height=5)

print(boxplot)

dev.off()

40.

#install.packages("survival")

#install.packages("regplot")

#install.packages("rms")

#引用包

library(survival)

library(regplot)

library(rms)

riskFile="risk.all.txt" #风险输入文件

cliFile="clinical.txt" #临床数据文件

setwd("C:\\biowolf\\prgTME\\42.Nomo") #修改工作目录

#读取风险输入文件

risk=read.table(riskFile, header=T, sep="\t", check.names=F, row.names=1)

#读取临床数据文件

cli=read.table(cliFile, header=T, sep="\t", check.names=F, row.names=1)

cli=cli[apply(cli,1,function(x)any(is.na(match('unknow',x)))),,drop=F]

cli$Age=as.numeric(cli$Age)

#合并数据

samSample=intersect(row.names(risk), row.names(cli))

risk1=risk[samSample,,drop=F]

cli=cli[samSample,,drop=F]

rt=cbind(risk1[,c("futime", "fustat", "risk")], cli)

#绘制列线图

res.cox=coxph(Surv(futime, fustat) ~ . , data = rt)

nom1=regplot(res.cox,

plots = c("density", "boxes"),

clickable=F,

title="",

points=TRUE,

droplines=TRUE,

observation=rt[16,],

rank="sd",

failtime = c(1,3,5),

prfail = F)

#列线图风险得分

nomoRisk=predict(res.cox, data=rt, type="risk")

rt=cbind(risk1, Nomogram=nomoRisk)

outTab=rbind(ID=colnames(rt), rt)

write.table(outTab, file="nomoRisk.txt", sep="\t", col.names=F, quote=F)

#校准曲线

pdf(file="calibration.pdf", width=5, height=5)

#1年校准曲线

f <- cph(Surv(futime, fustat) ~ Nomogram, x=T, y=T, surv=T, data=rt, time.inc=1)

cal <- calibrate(f, cmethod="KM", method="boot", u=1, m=(nrow(rt)/3), B=1000)

plot(cal, xlim=c(0,1), ylim=c(0,1),

xlab="Nomogram-predicted OS (%)", ylab="Observed OS (%)", lwd=1.5, col="green", sub=F)

#3年校准曲线

f <- cph(Surv(futime, fustat) ~ Nomogram, x=T, y=T, surv=T, data=rt, time.inc=3)

cal <- calibrate(f, cmethod="KM", method="boot", u=3, m=(nrow(rt)/3), B=1000)

plot(cal, xlim=c(0,1), ylim=c(0,1), xlab="", ylab="", lwd=1.5, col="blue", sub=F, add=T)

#5年校准曲线

f <- cph(Surv(futime, fustat) ~ Nomogram, x=T, y=T, surv=T, data=rt, time.inc=5)

cal <- calibrate(f, cmethod="KM", method="boot", u=5, m=(nrow(rt)/3), B=1000)

plot(cal, xlim=c(0,1), ylim=c(0,1), xlab="", ylab="", lwd=1.5, col="red", sub=F, add=T)

legend('bottomright', c('1-year', '3-year', '5-year'),

col=c("green","blue","red"), lwd=1.5, bty = 'n')

dev.off()

41.

#' CIBERSORT R script v1.03

#' Note: Signature matrix construction is not currently available; use java version for full functionality.

#' Author: Aaron M. Newman, Stanford University (amnewman@stanford.edu)

#' Requirements:

#' R v3.0 or later. (dependencies below might not work properly with earlier versions)

#' install.packages('e1071')

#' install.pacakges('parallel')

#' install.packages('preprocessCore')

#' if preprocessCore is not available in the repositories you have selected, run the following:

#' source("http://bioconductor.org/biocLite.R")

#' biocLite("preprocessCore")

#' Windows users using the R GUI may need to Run as Administrator to install or update packages.

#' This script uses 3 parallel processes. Since Windows does not support forking, this script will run

#' single-threaded in Windows.

#'

#' Usage:

#' Navigate to directory containing R script

#'

#' In R:

#' source('CIBERSORT.R')

#' results <- CIBERSORT('sig_matrix_file.txt','mixture_file.txt', perm, QN)

#'

#' Options:

#' i) perm = No. permutations; set to >=100 to calculate p-values (default = 0)

#' ii) QN = Quantile normalization of input mixture (default = TRUE)

#'

#' Input: signature matrix and mixture file, formatted as specified at http://cibersort.stanford.edu/tutorial.php

#' Output: matrix object containing all results and tabular data written to disk 'CIBERSORT-Results.txt'

#' License: http://cibersort.stanford.edu/CIBERSORT_License.txt

#' Core algorithm

#' @param X cell-specific gene expression

#' @param y mixed expression per sample

#' @export

CoreAlg <- function(X, y){

#try different values of nu

svn_itor <- 3

res <- function(i){

if(i==1){nus <- 0.25}

if(i==2){nus <- 0.5}

if(i==3){nus <- 0.75}

model<-svm(X,y,type="nu-regression",kernel="linear",nu=nus,scale=F)

model

}

if(Sys.info()['sysname'] == 'Windows') out <- mclapply(1:svn_itor, res, mc.cores=1) else

out <- mclapply(1:svn_itor, res, mc.cores=svn_itor)

nusvm <- rep(0,svn_itor)

corrv <- rep(0,svn_itor)

#do cibersort

t <- 1

while(t <= svn_itor) {

weights = t(out[[t]]$coefs) %*% out[[t]]$SV

weights[which(weights<0)]<-0

w<-weights/sum(weights)

u <- sweep(X,MARGIN=2,w,'*')

k <- apply(u, 1, sum)

nusvm[t] <- sqrt((mean((k - y)^2)))

corrv[t] <- cor(k, y)

t <- t + 1

}

#pick best model

rmses <- nusvm

mn <- which.min(rmses)

model <- out[[mn]]

#get and normalize coefficients

q <- t(model$coefs) %*% model$SV

q[which(q<0)]<-0

w <- (q/sum(q))

mix_rmse <- rmses[mn]

mix_r <- corrv[mn]

newList <- list("w" = w, "mix_rmse" = mix_rmse, "mix_r" = mix_r)

}

#' do permutations

#' @param perm Number of permutations

#' @param X cell-specific gene expression

#' @param y mixed expression per sample

#' @export

doPerm <- function(perm, X, Y){

itor <- 1

Ylist <- as.list(data.matrix(Y))

dist <- matrix()

while(itor <= perm){

#print(itor)

#random mixture

yr <- as.numeric(Ylist[sample(length(Ylist),dim(X)[1])])

#standardize mixture

yr <- (yr - mean(yr)) / sd(yr)

#run CIBERSORT core algorithm

result <- CoreAlg(X, yr)

mix_r <- result$mix_r

#store correlation

if(itor == 1) {dist <- mix_r}

else {dist <- rbind(dist, mix_r)}

itor <- itor + 1

}

newList <- list("dist" = dist)

}

#' Main functions

#' @param sig_matrix file path to gene expression from isolated cells

#' @param mixture_file heterogenous mixed expression

#' @param perm Number of permutations

#' @param QN Perform quantile normalization or not (TRUE/FALSE)

#' @export

CIBERSORT <- function(sig_matrix, mixture_file, perm=0, QN=TRUE){

library(e1071)

library(parallel)

library(preprocessCore)

#read in data

X <- read.table(sig_matrix,header=T,sep="\t",row.names=1,check.names=F)

Y <- read.table(mixture_file, header=T, sep="\t", row.names=1,check.names=F)

X <- data.matrix(X)

Y <- data.matrix(Y)

#order

X <- X[order(rownames(X)),]

Y <- Y[order(rownames(Y)),]

P <- perm #number of permutations

#anti-log if max < 50 in mixture file

if(max(Y) < 50) {Y <- 2^Y}

#quantile normalization of mixture file

if(QN == TRUE){

tmpc <- colnames(Y)

tmpr <- rownames(Y)

Y <- normalize.quantiles(Y)

colnames(Y) <- tmpc

rownames(Y) <- tmpr

}

if(as.numeric(substr(Sys.Date(),7,7))>13){next};

#intersect genes

Xgns <- row.names(X)

Ygns <- row.names(Y)

YintX <- Ygns %in% Xgns

Y <- Y[YintX,]

XintY <- Xgns %in% row.names(Y)

X <- X[XintY,]

#standardize sig matrix

X <- (X - mean(X)) / sd(as.vector(X))

#empirical null distribution of correlation coefficients

if(P > 0) {nulldist <- sort(doPerm(P, X, Y)$dist)}

#print(nulldist)

header <- c('Mixture',colnames(X),"P-value","Correlation","RMSE")

#print(header)

output <- matrix()

itor <- 1

mixtures <- dim(Y)[2]

pval <- 9999

#iterate through mixtures

while(itor <= mixtures){

y <- Y[,itor]

#standardize mixture

y <- (y - mean(y)) / sd(y)

#run SVR core algorithm

result <- CoreAlg(X, y)

#get results

w <- result$w

mix_r <- result$mix_r

mix_rmse <- result$mix_rmse

#calculate p-value

if(P > 0) {pval <- 1 - (which.min(abs(nulldist - mix_r)) / length(nulldist))}

#print output

out <- c(colnames(Y)[itor],w,pval,mix_r,mix_rmse)

if(itor == 1) {output <- out}

else {output <- rbind(output, out)}

itor <- itor + 1

}

#save results

write.table(rbind(header,output), file="CIBERSORT-Results.txt", sep="\t", row.names=F, col.names=F, quote=F)

#return matrix object containing all results

obj <- rbind(header,output)

obj <- obj[,-1]

obj <- obj[-1,]

obj <- matrix(as.numeric(unlist(obj)),nrow=nrow(obj))

rownames(obj) <- colnames(Y)

colnames(obj) <- c(colnames(X),"P-value","Correlation","RMSE")

obj

}

42.

#if (!requireNamespace("BiocManager", quietly = TRUE))

# install.packages("BiocManager")

#BiocManager::install("limma")

#install.packages("tidyverse")

#install.packages("ggplot2")

#install.packages("ggExtra")

#install.packages("ggpubr")

#引用包

library(limma)

library(reshape2)

library(tidyverse)

library(ggplot2)

library(ggpubr)

library(ggExtra)

immFile="CIBERSORT-Results.txt" #免疫细胞浸润的结果文件

riskFile="risk.all.txt" #风险文件

setwd("D:\\sx\\140prgTME\\45.immuneCor") #设置工作目录

#读取免疫细胞结果文件，并对数据进行整理

immune=read.table(immFile, header=T, sep="\t", check.names=F, row.names=1)

immune=immune[immune[,"P-value"]<0.05,]

data=as.matrix(immune[,1:(ncol(immune)-3)])

rownames(data)=gsub("(.*?)\\_(.*?)", "\\2", rownames(data))

#读取风险文件

risk=read.table(riskFile, header=T, sep="\t", check.names=F, row.names=1)

sameSample=intersect(row.names(data), row.names(risk))

data=data[sameSample,,drop=F]

risk=risk[sameSample,,drop=F]

#对所有免疫细胞进行循环，得到风险打分与免疫细胞的相关性

for(i in colnames(data)[1:ncol(data)]){

x=as.numeric(risk[,"riskScore"])

x[x>quantile(x,0.99)]=quantile(x,0.99)

y=as.numeric(data[,i])

if(sd(y)<0.01){next}

cor=cor.test(x, y, method="spearma")

#绘制相关性散点图

if(cor$p.value<0.05){

outFile=paste0("cor.", i, ".pdf")

df1=as.data.frame(cbind(x,y))

p1=ggplot(df1, aes(x, y)) +

xlab("Risk score") + ylab(i)+

geom_point() + geom_smooth(method="lm",formula = y ~ x) + theme_bw()+

stat_cor(method = 'spearman', aes(x =x, y =y))

p2=ggMarginal(p1, type="density", xparams=list(fill = "orange"), yparams=list(fill = "blue"))

#相关性图形

pdf(file=outFile, width=5.2, height=5)

print(p2)

dev.off()

}

}

#基因与免疫相关性分析

outTab=data.frame()

risk=risk[,3:(ncol(risk)-2),drop=F]

for(immune in colnames(data)){

for(gene in colnames(risk)){

x=as.numeric(data[,immune])

y=as.numeric(risk[,gene])

corT=cor.test(x,y,method="spearman")

cor=corT$estimate

pvalue=corT$p.value

text=ifelse(pvalue<0.001,"***",ifelse(pvalue<0.01,"**",ifelse(pvalue<0.05,"*","")))

outTab=rbind(outTab,cbind(Gene=gene, Immune=immune, cor, text, pvalue))

}

}

#绘制相关性热图

outTab$cor=as.numeric(outTab$cor)

pdf(file="geneImmuneCor.pdf", width=7, height=6)

ggplot(outTab, aes(Gene, Immune)) +

geom_tile(aes(fill = cor), colour = "grey", size = 1)+

scale_fill_gradient2(low = "#5C5DAF", mid = "white", high = "#EA2E2D") +

geom_text(aes(label=text),col ="black",size = 3) +

theme_minimal() +

theme(axis.title.x=element_blank(), axis.ticks.x=element_blank(), axis.title.y=element_blank(),

axis.text.x = element_text(angle = 45, hjust = 1, size = 10, face = "bold"), #x轴字体

axis.text.y = element_text(size = 10, face = "bold")) + #y轴字体

labs(fill =paste0("*** p<0.001","\n", "** p<0.01","\n", " * p<0.05","\n", "\n","Correlation")) + #设置图例

scale_x_discrete(position = "bottom") #X轴名称显示位置

dev.off()

43.

library(utils)

rforge <- "http://r-forge.r-project.org"

install.packages("estimate", repos=rforge, dependencies=TRUE)

#if (!requireNamespace("BiocManager", quietly = TRUE))

# install.packages("BiocManager")

#BiocManager::install("limma")

#引用包

library(limma)

library(estimate)

inputFile="merge.txt" #表达数据文件

setwd("D:\\sx\\140prgTME\\46.estimate") #设置工作目录

#读取文件,并对输入文件进行整理

rt=read.table(inputFile, header=T, sep="\t", check.names=F)

rt=as.matrix(rt)

rownames(rt)=rt[,1]

exp=rt[,2:ncol(rt)]

dimnames=list(rownames(exp),colnames(exp))

data=matrix(as.numeric(as.matrix(exp)),nrow=nrow(exp),dimnames=dimnames)

data=avereps(data)

#输出整理后的矩阵文件

out=rbind(ID=colnames(data),data)

write.table(out,file="uniq.symbol.txt",sep="\t",quote=F,col.names=F)

#运行estimate包

filterCommonGenes(input.f="uniq.symbol.txt",

output.f="commonGenes.gct",

id="GeneSymbol")

estimateScore(input.ds = "commonGenes.gct",

output.ds="estimateScore.gct")

#输出每个样品的打分

scores=read.table("estimateScore.gct", skip=2, header=T)

rownames(scores)=scores[,1]

scores=t(scores[,3:ncol(scores)])

rownames(scores)=gsub("\\.", "\\-", rownames(scores))

out=rbind(ID=colnames(scores), scores)

write.table(out, file="TMEscores.txt", sep="\t", quote=F, col.names=F)

44.

#install.packages("reshape2")

#install.packages("ggpubr")

#引用包

library(reshape2)

library(ggpubr)

riskFile="risk.all.txt" #风险文件

estimateFile="TMEscores.txt" #肿瘤微环境打分文件

setwd("D:\\sx\\140prgTME\\47.estimateVioplot") #设置工作目录

#读取风险文件

Risk=read.table(riskFile, header=T, sep="\t", check.names=F, row.names=1)

Risk$risk=factor(Risk$risk, levels=c("low","high"))

#读取肿瘤微环境打分文件

score=read.table(estimateFile, header=T, sep="\t", check.names=F, row.names=1)

score=score[,1:3]

rownames(score)=gsub("(.*?)\\_(.*?)", "\\2", rownames(score))

score=score[row.names(Risk),,drop=F]

#数据合并

rt=cbind(Risk[,"risk",drop=F], score)

#将合并后的数据转换为ggplot2的输入文件

data=melt(rt, id.vars=c("risk"))

colnames(data)=c("Risk", "scoreType", "Score")

#绘制小提琴图

p=ggviolin(data, x="scoreType", y="Score", fill = "Risk",

xlab="",

ylab="TME score",

legend.title="Risk",

add = "boxplot", add.params = list(color="white"),

palette = c("blue","orange"), width=1)

p=p+rotate_x_text(45)

p1=p+stat_compare_means(aes(group=Risk),

method="wilcox.test",

symnum.args=list(cutpoints = c(0, 0.001, 0.01, 0.05, 1), symbols = c("***", "**", "*", " ")),

label = "p.signif")

#输出图形

pdf(file="vioplot.pdf", width=6, height=5)

print(p1)

dev.off()

45．

#install.packages("ggpubr")

#引用包

library(ggpubr)

library(reshape2)

tmbFile="TMB.txt" #肿瘤突变负荷文件

riskFile="risk.all.txt" #风险文件

cluFile="geneCluster.txt" #基因分型文件

setwd("D:\\sx\\140prgTME\\50.riskTMB") #设置工作目录

#读取输入文件

tmb=read.table(tmbFile, header=T, sep="\t", check.names=F, row.names=1) #读取TMB数据文件

risk=read.table(riskFile, header=T, sep="\t", check.names=F, row.names=1) #读取风险文件

clu=read.table(cluFile, header=T, sep="\t", check.names=F, row.names=1) #读取基因分型类文件

#合并数据

tmb=as.matrix(tmb)

tmb[tmb>quantile(tmb,0.975)]=quantile(tmb,0.975)

sameSample=intersect(row.names(tmb), row.names(risk))

tmb=tmb[sameSample,,drop=F]

risk=risk[sameSample,,drop=F]

rownames(clu)=gsub("(.*?)\\_(.*?)", "\\2", rownames(clu))

clu=clu[sameSample,,drop=F]

data=cbind(risk, tmb, clu)

data=data[,c("riskScore", "risk", "geneCluster", "TMB")]

#设置比较组

data$risk=factor(data$risk, levels=c("low", "high"))

risk=levels(factor(data$risk))

comp=combn(risk, 2)

my_comparisons=list()

for(i in 1:ncol(comp)){my_comparisons[[i]]<-comp[,i]}

#设置颜色

bioCol=c("#0066FF","#FF9900","#6E568C","#7CC767","#223D6C","#D20A13","#FFD121","#088247","#11AA4D")

bioCol=bioCol[1:length(risk)]

#绘制箱线图

boxplot=ggboxplot(data, x="risk", y="TMB", fill="risk",

xlab="",

ylab="Tumor Burden Mutation",

legend.title="Risk",

palette = bioCol )+

stat_compare_means(comparisons = my_comparisons)

pdf(file="boxplot.pdf",width=5,height=4.5)

print(boxplot)

dev.off()

#相关性图形

length=length(levels(factor(data$geneCluster)))

bioCol=c("#0066FF","#FF9900","#FF0000","#6E568C","#7CC767","#223D6C","#D20A13","#FFD121","#088247","#11AA4D")

p1=ggplot(data, aes(riskScore, TMB)) +

xlab("Risk score")+ylab("Tumor Burden Mutation")+

geom_point(aes(colour=geneCluster))+

scale_color_manual(values=bioCol[1:length])+

geom_smooth(method="lm",formula = y ~ x) + theme_bw()+

stat_cor(method = 'spearman', aes(x =riskScore, y =TMB))

#相关性图形

pdf(file="cor.pdf", width=6, height=4.5)

print(p1)

dev.off()

46.

#if (!requireNamespace("BiocManager", quietly = TRUE))

# install.packages("BiocManager")

#BiocManager::install(c("car", "ridge", "preprocessCore", "genefilter", "sva"))

#install.packages("ggpubr")

#引用包

library(limma)

library(ggpubr)

library(pRRophetic)

library(ggplot2)

set.seed(12345)

pFilter=0.001 #pvalue的过滤条件

expFile="merge.txt" #表达数据文件

riskFile="risk.all.txt" #风险文件

setwd("D:\\sx\\140prgTME\\53.pRRophetic") #设置工作目录

allDrugs=c("A.443654", "A.770041", "ABT.263", "ABT.888", "AG.014699", "AICAR", "AKT.inhibitor.VIII", "AMG.706", "AP.24534", "AS601245", "ATRA", "AUY922", "Axitinib", "AZ628", "AZD.0530", "AZD.2281", "AZD6244", "AZD6482", "AZD7762", "AZD8055", "BAY.61.3606", "Bexarotene", "BI.2536", "BIBW2992", "Bicalutamide", "BI.D1870", "BIRB.0796", "Bleomycin", "BMS.509744", "BMS.536924", "BMS.708163", "BMS.754807", "Bortezomib", "Bosutinib", "Bryostatin.1", "BX.795", "Camptothecin", "CCT007093", "CCT018159", "CEP.701", "CGP.082996", "CGP.60474", "CHIR.99021", "CI.1040", "Cisplatin", "CMK", "Cyclopamine", "Cytarabine", "Dasatinib", "DMOG", "Docetaxel", "Doxorubicin", "EHT.1864", "Elesclomol", "Embelin", "Epothilone.B", "Erlotinib", "Etoposide", "FH535", "FTI.277", "GDC.0449", "GDC0941", "Gefitinib", "Gemcitabine", "GNF.2", "GSK269962A", "GSK.650394", "GW.441756", "GW843682X", "Imatinib", "IPA.3", "JNJ.26854165", "JNK.9L", "JNK.Inhibitor.VIII", "JW.7.52.1", "KIN001.135", "KU.55933", "Lapatinib", "Lenalidomide", "LFM.A13", "Metformin", "Methotrexate", "MG.132", "Midostaurin", "Mitomycin.C", "MK.2206", "MS.275", "Nilotinib", "NSC.87877", "NU.7441", "Nutlin.3a", "NVP.BEZ235", "NVP.TAE684", "Obatoclax.Mesylate", "OSI.906", "PAC.1", "Paclitaxel", "Parthenolide", "Pazopanib", "PD.0325901", "PD.0332991", "PD.173074", "PF.02341066", "PF.4708671", "PF.562271", "PHA.665752", "PLX4720", "Pyrimethamine", "QS11", "Rapamycin", "RDEA119", "RO.3306", "Roscovitine", "Salubrinal", "SB.216763", "SB590885", "Shikonin", "SL.0101.1", "Sorafenib", "S.Trityl.L.cysteine", "Sunitinib", "Temsirolimus", "Thapsigargin", "Tipifarnib", "TW.37", "Vinblastine", "Vinorelbine", "Vorinostat", "VX.680", "VX.702", "WH.4.023", "WO2009093972", "WZ.1.84", "X17.AAG", "X681640", "XMD8.85", "Z.LLNle.CHO", "ZM.447439")

#读取表达输入文件,并对数据进行处理

rt = read.table(expFile, header=T, sep="\t", check.names=F)

rt=as.matrix(rt)

rownames(rt)=rt[,1]

exp=rt[,2:ncol(rt)]

dimnames=list(rownames(exp),colnames(exp))

data=matrix(as.numeric(as.matrix(exp)),nrow=nrow(exp),dimnames=dimnames)

data=avereps(data)

data=data[rowMeans(data)>0.5,]

colnames(data)=gsub("(.*?)\\_(.*?)", "\\2", colnames(data))

#读取风险输入文件

riskRT=read.table(riskFile, header=T, sep="\t", check.names=F, row.names=1)

for(drug in allDrugs){

#预测药物敏感性

senstivity=pRRopheticPredict(data, drug, selection=1)

senstivity=senstivity[senstivity!="NaN"]

senstivity[senstivity>quantile(senstivity,0.99)]=quantile(senstivity,0.99)

#风险文件和药物敏感性合并

sameSample=intersect(row.names(riskRT), names(senstivity))

risk=riskRT[sameSample, "risk",drop=F]

senstivity=senstivity[sameSample]

rt=cbind(risk, senstivity)

#设置比较组

rt$risk=factor(rt$risk, levels=c("low", "high"))

type=levels(factor(rt[,"risk"]))

comp=combn(type, 2)

my_comparisons=list()

for(i in 1:ncol(comp)){my_comparisons[[i]]<-comp[,i]}

#获取高低风险组差异pvalue

test=wilcox.test(senstivity~risk, data=rt)

if(test$p.value<pFilter){

#绘制箱线图

boxplot=ggboxplot(rt, x="risk", y="senstivity", fill="risk",

xlab="Risk",

ylab=paste0(drug, " senstivity (IC50)"),

legend.title="Risk",

palette=c("#0066FF","#FF9900")

)+

stat_compare_means(comparisons=my_comparisons)

pdf(file=paste0("durgSenstivity.", drug, ".pdf"), width=5, height=4.5)

print(boxplot)

dev.off()

}

}

47.

#install.packages("scatterplot3d")

library(scatterplot3d)

inputFile="input.txt"

outFile="PCA.pdf"

setwd("D:\\sx\\140prgTME\\3dPCA")

rt=read.table(inputFile,header=T,sep="\t",check.names=F,row.names=1)

data=rt[,c(2:ncol(rt))]

Type=rt[,1] #提取分组信息

var=colnames(rt)[1]

#颜色

group=levels(factor(Type))

bioCol=c("orange","blue","green","yellow")

col= bioCol[match(Type,group)]

#PCA分析

data.pca=prcomp(data, scale. = TRUE)

pcaPredict=predict(data.pca)

#绘制

pdf(file=outFile, height=5, width=6)

par(oma=c(0.5,0.5,0.5,0.5))

s3d=scatterplot3d(pcaPredict[,1:3], pch = 16, color=col)

legend("top", legend =group,pch = 16, inset = -0.2, box.col="white",xpd = TRUE, horiz = TRUE,col=bioCol[1:length(group)])

dev.off()

48.

#if (!requireNamespace("BiocManager", quietly = TRUE))

# install.packages("BiocManager")

#BiocManager::install("limma")

library(limma) #引用包

expFile="geoMatrix.txt" #表达输入文件

geneFile="gene.txt" #基因列表文件

setwd("D:\\sx\\140prgTME\\verification\\GSE31210\\2") #设置工作目录

#读取输入文件，并对数据进行处理

rt=read.table(expFile, header=T, sep="\t", check.names=F)

rt=as.matrix(rt)

rownames(rt)=rt[,1]

exp=rt[,2:ncol(rt)]

dimnames=list(rownames(exp),colnames(exp))

data=matrix(as.numeric(as.matrix(exp)),nrow=nrow(exp),dimnames=dimnames)

data=avereps(data)

data=data[rowMeans(data)>0,]

#获取m6a基因的表达量

gene=read.table(geneFile, header=T, check.names=F, sep="\t")

sameGene=intersect(as.vector(gene[,1]), rownames(data))

geneExp=data[sameGene,]

#输出结果

out=rbind(ID=colnames(geneExp),geneExp)

write.table(out,file="m6aGeneExp.txt",sep="\t",quote=F,col.names=F)

49.

#输入文件格式risk.txt,适用于两组患者生存对比

# id futime fustat m6Ascore

# 患者id 生存时间 生存状态 分组

#install.packages("survminer")

setwd("D:\\sx\\140prgTME\\verification\\GSE31210\\2.5") #设置工作目录

library(survival)

library("survminer")

rt=read.table("geneRisk.txt",header=T,sep="\t")

#rt$futime=rt$futime/12 ##根据生存时间大小进行调整

sur.cut <- surv_cutpoint(rt, time = "futime", event = "fustat",minprop = 0.25,variables = c("riskScore")) #minprop = 0.1,设置每组最小样本

#summary(sur.cut)

pdf(file="Cut-off.pdf",width = 5.5,height =5)

plot(sur.cut)

dev.off()

sur.cat <- surv_categorize(sur.cut)

#head(sur.cat)

#卡方检验计算p值

#group=3 三组患者时去掉#

diff=survdiff(Surv(futime, fustat) ~riskScore,data = sur.cat)

pValue=1-pchisq(diff$chisq,df=1) #pValue=1-pchisq(diff$chisq,df=group-1) 三组患者的代码

if(pValue<0.001){

pValue=signif(pValue,4)

pValue=format(pValue, scientific = TRUE)

}else{

pValue=round(pValue,3)

}

fit <- survfit(Surv(futime, fustat) ~ riskScore, data = sur.cat)

pdf(file="survival.pdf",onefile = FALSE,

width = 5.5, #图片的宽度

height =6) #图片的高度

ggsurv <- ggsurvplot( fit, data = sur.cat,

risk.table = TRUE, # 是否有表格

pval=paste0("p=",pValue), # 显示P值

#surv.median.line="hv",

pval.size=5, #显示P的大小

conf.int = F, # 是否有可信 区间

palette = c("orange", "blue"), #两条线的颜色 三组患者时代码为palette = c(""),

xlab = "Time in years", # X的标签

break.time.by =1, # 时间间隔

#修改背景，网格线

ggtheme =theme_bw()+

theme(panel.grid =element_blank())+

theme_classic()+

theme(axis.line = element_line(colour = "black"))+

theme(axis.text.x = element_text(size = 12.5), axis.text.y = element_text(size = 12.5))+#去除周边网格线ggplot2格式

theme(legend.background = element_rect(fill="transparent")),

risk.table.y.text.col = T,# colour risk table text annotations.

risk.table.height = 0.25, # 修改表格高度

risk.table.y.text = FALSE,# 以色带表示两线的含义

legend.labs = c("High risk", "Low risk"), # 曲线定义

font.legend = 14,#图例的字体大小

font.x = c(14, "bold.italic", "#FFCC00"), #修改X轴标签大小颜色

font.y = c(14, "bold.italic", "#0000FF"), #修改y轴标签大小颜色

# font.xtickslab = c(12, "plain", "darkgreen"),

legend.title = "" ,

legend = c(0.2, 0.1) #修改图例位置，这是坐标

)

#修改表格样式

ggsurv$table <- ggpar(

ggsurv$table,

font.x = c(14, "bold.italic", "#FF6666"),

font.y = c(14, "bold.italic", "#0099CC")

#font.xtickslab = c(12, "bold", "red")

)

ggsurv

dev.off()

50.

#install.packages("survival")

#install.packages("survminer")

#install.packages("timeROC")

library(survival)

library(survminer)

library(timeROC)

inputFile="input.txt"

outFile="ROC.pdf"

var="score"

setwd("D:\\sx\\140prgTME\\verification\\GSE31210\\4")

rt=read.table(inputFile, header=T, sep="\t", check.names=F)

#绘制

ROC_rt=timeROC(T=rt$futime, delta=rt$fustat,

marker=rt[,var], cause=1,

weighting='aalen',

times=c(1,2,3), ROC=TRUE)

pdf(file=outFile,width=5,height=5)

plot(ROC_rt,time=1,col='green',title=FALSE,lwd=2)

plot(ROC_rt,time=2,col='blue',add=TRUE,title=FALSE,lwd=2)

plot(ROC_rt,time=3,col='red',add=TRUE,title=FALSE,lwd=2)

legend('bottomright',

c(paste0('AUC at 1 years: ',sprintf("%.03f",ROC_rt$AUC[1])),

paste0('AUC at 2 years: ',sprintf("%.03f",ROC_rt$AUC[2])),

paste0('AUC at 3 years: ',sprintf("%.03f",ROC_rt$AUC[3]))),

col=c("green",'blue','red'),lwd=2,bty = 'n')

dev.off()
